# Design and synthesis of novel ureido and thioureido conjugated hydrazone derivatives with potent anticancer activity

**DOI:** 10.1186/s13065-022-00873-3

**Published:** 2022-11-01

**Authors:** Nasrin Nassiri Koopaei, Mehrasa Shademani, Nasrin Shirzad Yazdi, Raheleh Tahmasvand, Mina Dehbid, Mansur Nassiri Koopaei, Homa Azizian, Zahra Mousavi, Ali Almasirad, Mona Salimi

**Affiliations:** 1 Department of Medicinal Chemistry, Faculty of Pharmacy, Tehran Medical Sciences, Islamic Azad University, P.O. Box 1941933111, Tehran, Iran; 2 Department of Physiology and Pharmacology, Pasteur Institute of Iran, P.O. Box 1316943551, Tehran, Iran; 3 Department of Pharmacology & Toxicology, Faculty of Pharmacy, Tehran Medical Sciences, Islamic Azad University, Tehran, Iran; 4 Department of Clinical Pharmacy, School of Pharmacy, Shiraz University of Medical Sciences, Shiraz, Iran; 5 Department of Biology, Faculty of Science, University of Guilan, Rasht, Iran; 6 Department of Medicinal Chemistry, Faculty of Pharmacy and Drug Design & Development Research Center, The Institute of Pharmaceutical Sciences (TIPS), Tehran University of Medical Sciences, Tehran, Iran; 7 Department of Medicinal Chemistry, School of Pharmacy-International Campus, Iran University of Medical Science, Tehran, Iran

**Keywords:** Ureides, Anticancer, Apoptosis, Hydrazone, Colon cancer, Hepatocellular carcinoma

## Abstract

**Background:**

Compounds possessing urea/thiourea moiety have a wide range of biological properties including anticancer activity. On the other hand, taking advantage of the low toxicity and structural diversity of hydrazone derivatives, they are presently being considered for designing chemical compounds with hydrazone moiety in the field of cancer treatment. With this in mind, a series of novel ureido/thioureido derivatives possessing a hydrazone moiety bearing *nitro* and *chloro* substituents (**4a**–**4i**) have been designed, synthesized, characterized and evaluated for their in vitro cytotoxic effect on HT-29 human colon carcinoma and HepG2 hepatocarcinoma cell lines.

**Results:**

Two compounds (**4c** and **4e**) having the chloro phenylurea group hybridized with phenyl hydrazone bearing *nitro* or *chloro* moieties demonstrated potent anticancer effect with the IC_50_ values between 2.2 and 4.8 µM at 72 h. The mechanism of action of compound **4c** was revealed in hepatocellular carcinoma cells as an inducer of apoptosis in a caspase-independent pathway.

**Conclusion:**

Taken together, the current work presented compound **4c** as a potential lead compound in developing future hepatocellular carcinoma chemotherapy drugs.

**Methods:**

The compounds were synthesized and then characterized by physical and spectral data (FT-IR, ^1^H-NMR, ^13^C-NMR, Mass). The anticancer activity was assessed using MTT assay, flowcytometry, annexin-V, DAPI staining and Western blot analysis.

**Graphical Abstract:**

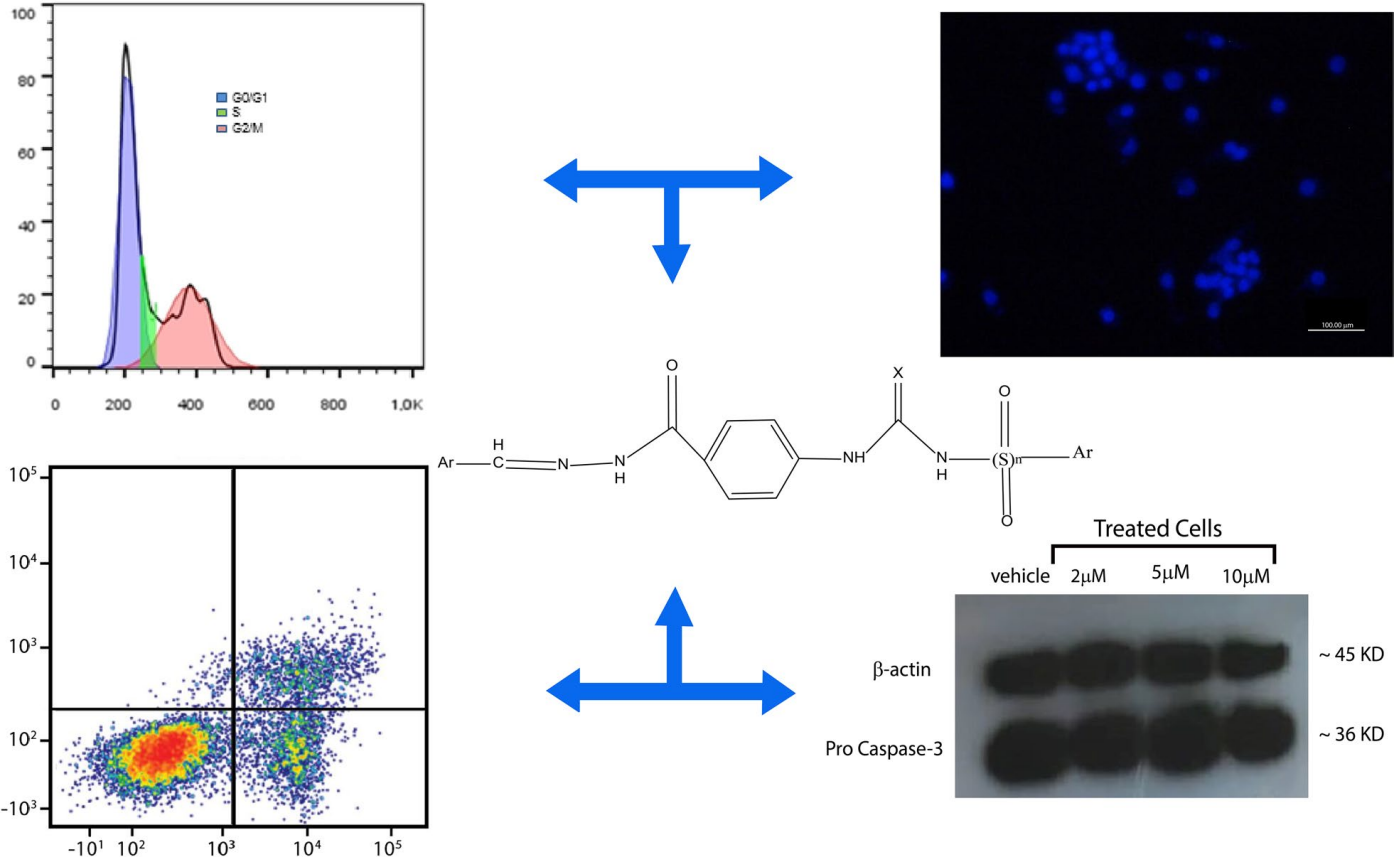

**Supplementary Information:**

The online version contains supplementary material available at 10.1186/s13065-022-00873-3.

## Introduction

Despite advances in developing new cancer treatments, cancer has still remained the major health issue worldwide due to numerous factors [1–3]. Since traditional chemotherapy drugs have undesirable side effects, exploring a new generation of anticancer agents with apoptosis inducing mechanism of action and more safety seems to be of challenge [4]. To this end, investigators have directed a considerable interest towards multiple targeted agents, which afford effective and specific chemotherapeutics with low toxicity.

Among the different types of cancer, here, we focused on colon and hepatocellular carcinoma as the two complicated forms of cancer. Colon cancer is diagnosed as the second and third most common cancer in women and men, respectively, accounting for 10% of all annually diagnosed cancers and cancer-related deaths worldwide [5]. The prevalence of colon cancer carries an economic burden to healthcare systems, and there are still questions about how best to treat colon cancer, despite advances in treatment options for this type of cancer [6]. Notably, liver metastasis is the biggest issue in patients suffering from advance colorectal cancer stage [7]. On the other hand, hepatocellular carcinoma (HCC) accounts for approximately more than 80% of cases of liver cancer. World Health Organization (WHO) regards HCC as the second leading cause of cancer deaths [8, 9]. Indeed, HCC is one of the most prominent challenges in terms of treatment in the clinical area, owing to the different molecular pathways involved in its development [10]. Hence, discovering novel agents to influence these two types of cancers is of great interest.

Among the most versatile bioactive molecules, compounds possessing urea/thiourea moiety represent a wide range of biological properties including anticancer activity [11–17]. The anticancer effect reported for these structures is associated with their ability to block several tyrosine kinase receptors involved in proliferation and angiogenesis [18–21]. In this context, a number of urea derivatives comprise an important class of promising chemotherapeutic drugs [12–17]. Interestingly, the potentiality of diaryl urea and thiourea derivatives has been pointed out by numerous publications owing to their unique binding mode and kinase inhibition profile [18–21]. Sorafenib, a multi-targeted diaryl urea small molecule acting as a kinase inhibitor has been approved by FDA and launched for treatment of patients with renal and hepatocellular carcinomas [22, 23]. Moreover, ureides structures display an enzymatic hydrolysis resistance. These evidences make in favor of inserting the urea as a core pharmacophore in novel efficient anticancer agents [12–15, 24]. On the other hand, taking advantage of their low toxicity and structural diversity, hydrazone derivatives are presently being considered for designing chemical compounds with hydrazone moiety in the field of cancer treatment [25]. The hydrazide-hydrazones show their anti-cancer activity through different mechanisms including the induction of apoptosis, prevention of microtubule polymerization, inhibition of cyclin-dependent kinases, blockage of histone deacetylases and phosphatidylinositol 3-kinases [25–36]. These findings prompted us to go further with our preceding studies to determine the potential anti-proliferative activity of the two moieties.

**Scheme 1:**
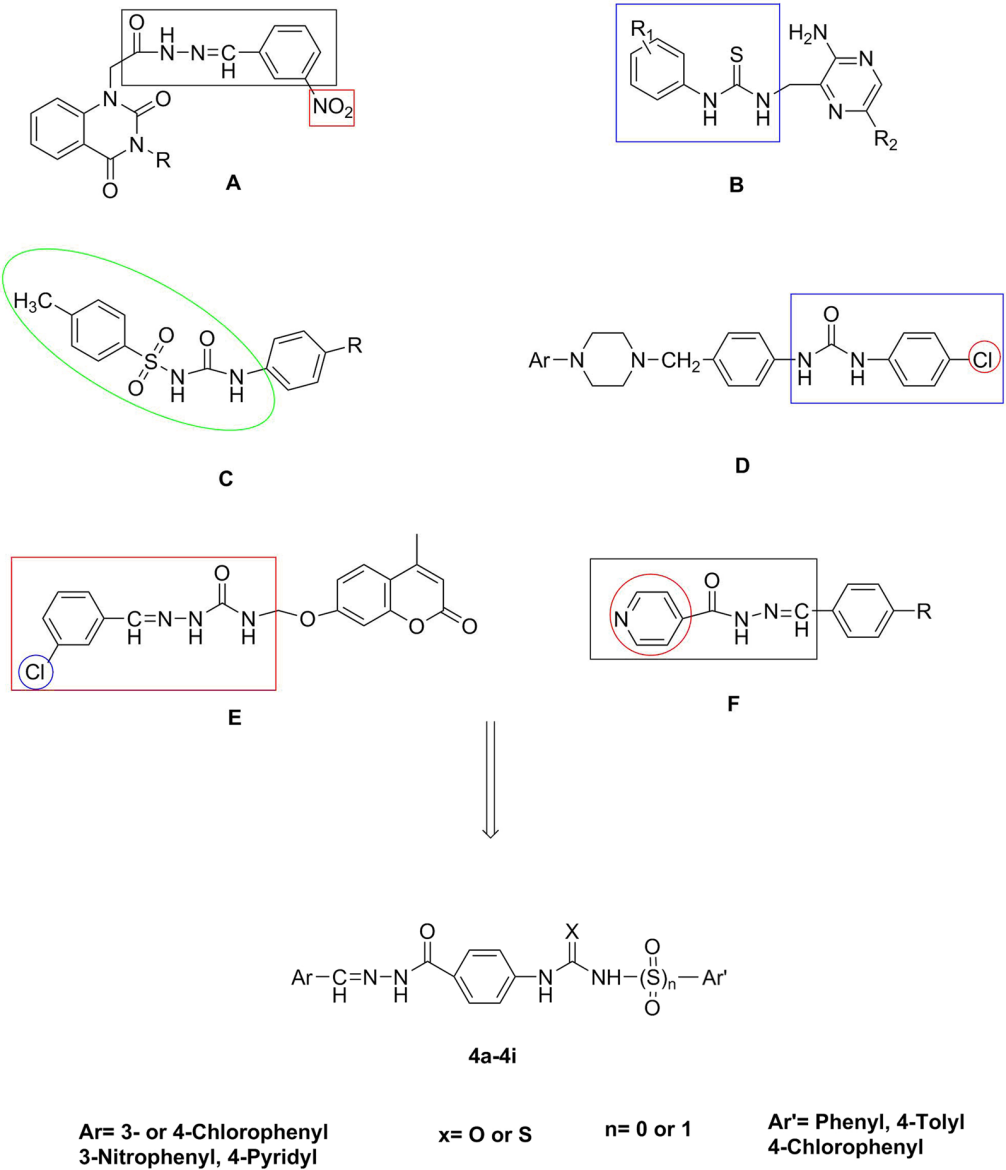
Design of target compounds **4a**–**4i**

Molecular hybridization is one of the rational drug design methods that covalently associates two or more drug pharmacophores into a single compound and is considered as an efficient approach to design highly active and novel entities. Herein, we used this technique to design a few compounds based upon the principle of conjugating the two pharmacophores (Scheme 1); i.e. ureides and hydrazones, with the assumption that the hybrids possess a greater potential of anticancer activity, act through multiple mechanisms of action and bind with a higher affinity to a target receptor. As shown in Scheme 1, various chemical compounds with anticancer properties were selected according to the literature with their important pharmacophores specified in the colored boxes [31, 32, 37–41]. In this structure design, we also changed the substituents as an ordinary tool in drug design in medicinal chemistry to find the most powerful compounds with the ability to inhibit human cancer cell growth. Moreover, the *meta* or *para* position of the substituted phenyl as well as 4-pyridyl or 4-methylphenyl sulfonyl moieties were selected based on previous literature reporting them as effective fragments [42–46] (Scheme 1). In this work, we report on the synthesis of compounds bearing hydrazone and urea moieties, namely compounds **4a–4i** (Table 1), and their in vitro anticancer activity against human cancer cell lines.

**Table 1:** The structure of compounds **4a**–**4i**


Compound	Ar	Ar′	x	MW	Formula
**4a**			O	392.5	C_21_H_17_ClN _4_O_2_
**4b**			S	408.5	C_21_H_17_ClN_4_OS
**4c**			O	427	C_21_H_16_Cl_2_N_4_O_2_
**4d**			O	470.5	C_22_H_19_ClN_4_O_4_S
**4e**			O	437.5	C_21_H_16_ Cl N_5_O_4_
**4f**			O	403	C_21_H_17_N_5_O_4_
**4 g**			O	481	C_22_H_19_N_5_O_6_S
**4 h**			O	427	C_21_H_16_Cl_2_N_4_O_2_
**4i**			O	437	C_21_H_19_N_5_O_4_S

## Results

### Chemistry

**Scheme 2:**
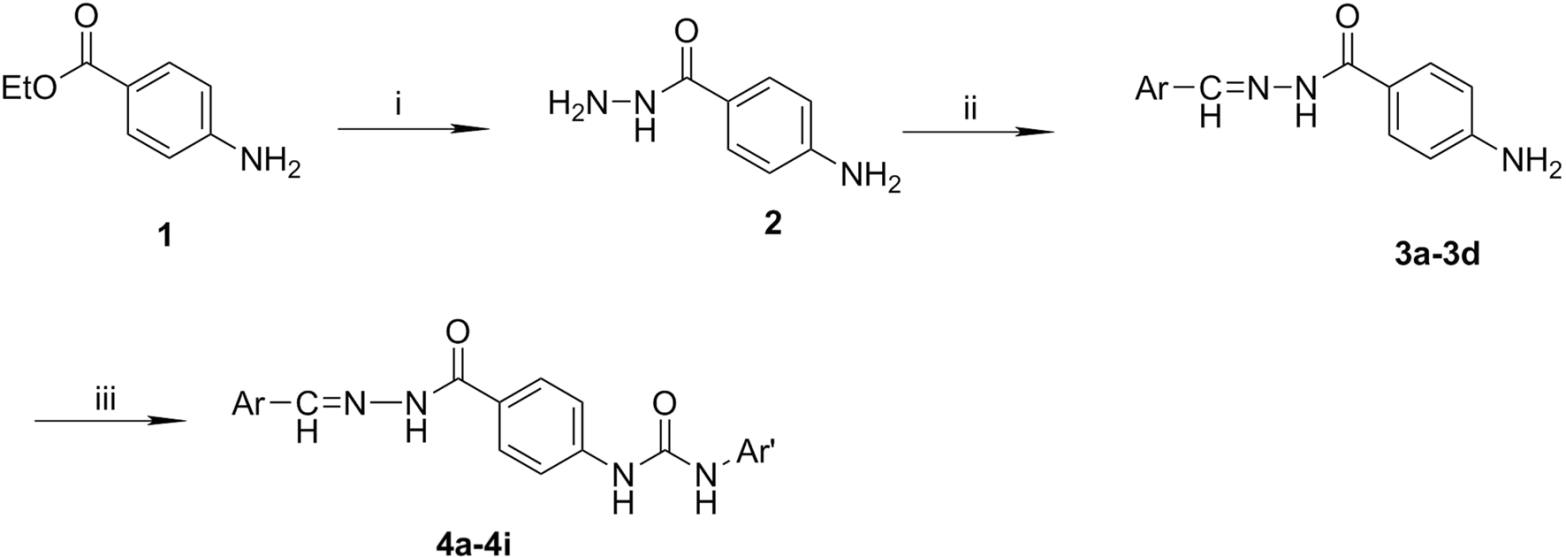
Synthesis of compounds **4a**–**4i**. Reagents and conditions: **i** NH2NH2.H2O, Absolute EtOH, r.t., 96 h **ii** Ar-CHO, HCl, EtOH, r.t., 30 min; **iii** Ar′–N=C=X or Ar′–SO2–N=C=O, Acetone/DMF, reflux, 1 h

A diverse array of derivatives (**4a–4i**) were synthesized according to Scheme 2 and characterized by physical and spectral data (FT-IR, ^1^H-NMR, ^13^C-NMR, and Mass spectra) (Additional file 1). The synthesis of some related biologically active compounds has been reported, previously [47].

### Anti-proliferative activity of compounds 4a–4i

**Fig. 1:**
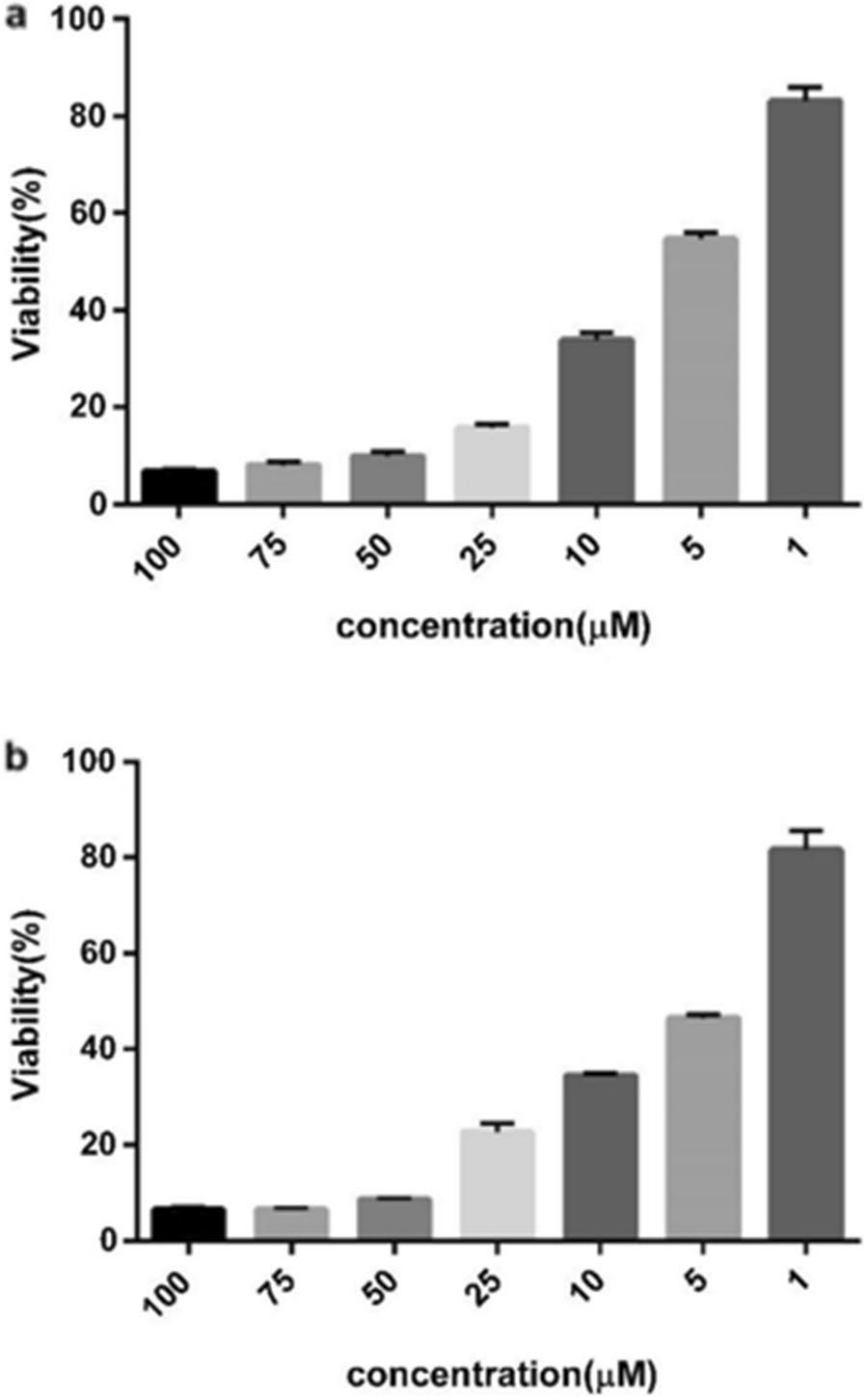
Percentage of viability for compounds **a 4c** and **b 4e** on NIH3T3 cells at 72 h. Values are presented as mean ± SEM of three independent experiments, performed in triplicate

We initially synthesized several urea and thiourea derivatives combined with phenyl hydrazones (compounds **4a–4i**) and assessed their cytotoxicity against two human cancer cell lines, namely HT-29 colon adenocarcinoma and HepG2 hepatocarcinoma cells, using an MTT-based assay (Additional file 2: Table S1). Compounds inducing a cytotoxicity of more than 60% at 72 h were further selected to quantify their ability to inhibit cell growth by determining their IC_50_ values against the tested cells after 24, 48 and 72 h of incubation. As shown in Tables 2 and 3, compound **4c** was more effective towards HepG2 than HT-29 cell lines with an IC_50_ value of 2.22 µM, whereas compound **4e** with an IC_50_ value of 3.4 µM revealed a greater potential for HT-29 cell growth inhibition at 72 h. The IC_50_ value of Doxorubicin was calculated as positive control at 72 h and reported in the Additional file 2: Table S2. The control cell line NIH3T3 exposed to the two compounds was viable on day three of incubation period (Fig. 1). Selectivity index (SI) manifested a selection of the compounds activity between normal fibroblastic and cancer cells. Following a close inspection of the IC_50_ values, further experiments were performed on HT-29 and HepG2 cell lines.

**Table 2:** IC_50_ values for cytotoxic activity of compound **4c** towards cancer cells at 72 h

Cell lines	Time (h)		
	24 h	48 h	72 h
HepG2	5.30 ± 1.11	5.34 ± 1.08	2.22 ± 1.16
HT-29	4.69 ± 1.23	4.69 ± 1.23	3.72 ± 1.08

Values were determined at least three independent experiments each performed in triplicate and expressed as mean ± SEM

**Table 3:** IC_50_ values for cytotoxic activity of compound **4e** towards cancer cells at 72 h

Cell lines	Time (h)		
	24 h	48 h	72 h
HepG2	12.14 ± 1.14	10.01 ± 1.14	4.82 ± 1.08
HT-29	3.72 ± 1.17	3.63 ± 1.14	3.40 ± 1.10

Values were determined at least three independent experiments each performed in triplicate and expressed as mean ± SEM

### Apoptosis induction

**Fig. 2:**
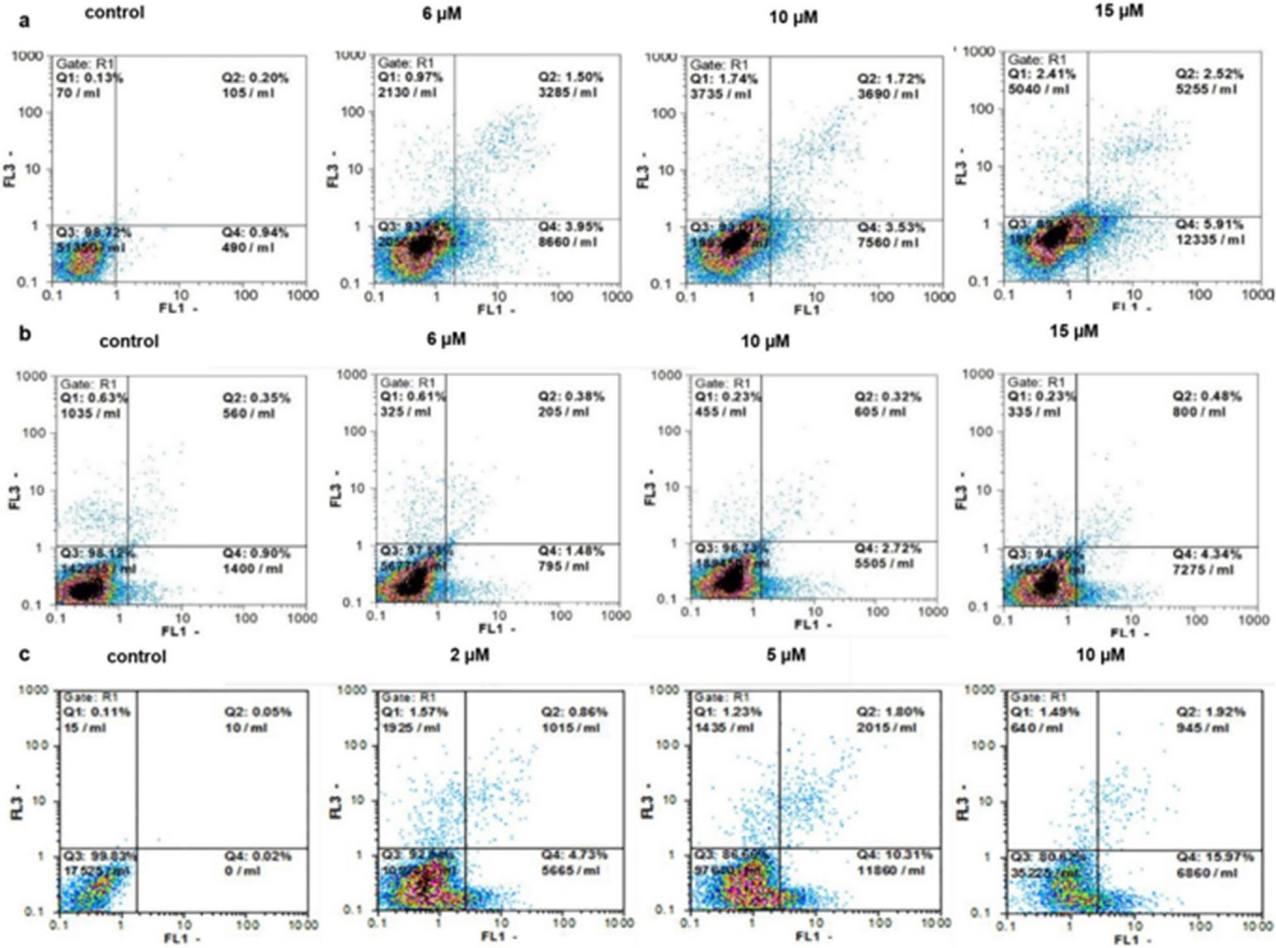
Annexin-V/PI flowcytometric analysis to quantify apoptosis induced by compound **a** **4c** and **b** **4e** in HT-29 and compound **c** **4c** in HepG2 cells for 48 h. The results shown are representatives of three independent experiments. Quadrant 4, living cells An −/PI −; Quadrant 3, early apoptotic cells An +/PI −; Quadrant 2, late apoptotic cells An +/PI +; Quadrant 1, necrotic cells An −/PI +

Apoptosis is regarded as a main approach through which most of the anticancer drugs destroy cancer cells [48, 49]. To explore whether the cell growth inhibition induced by the hybrids was apoptosis-related, HT-29 cells were treated with compounds **4c** and **4e** at different concentrations (6, 10, 15 µM) for 48 h, considering the IC_50_ values addressing the potency of the two compounds in this cell line. In addition, HepG2 cells were also incubated with 2, 5 and 10 µM of compound **4c** for 48 h, considering the higher efficiency of this compound on HepG2 relative to HT-29 cells. Apoptosis inducing activity was determined by biparametric flow cytometric analysis utilizing propidium iodide (PI) and annexin-V-FLUOS. As shown in Tables 4 and 5, compound **4c** was able to induce apoptosis at an early stage in HT-29 cells to a greater extent than was compound **4e**. In this regard, the apoptosis rate in the early stage was significantly increased from 0.99% in the control/vehicle group to 2.5 and 3.8% in the 10 and 15 µM of compound-**4e**-treated group, respectively, after 48 h of incubation of HT-29 cells. This is while a significant proportion of the cell population was in the late stage of apoptosis following the treatment of HT-29 cells with compound **4c** at 6, 10 and 15 µM. In the case of HepG2 cells, the number of early and late apoptotic cells increased gradually following treatment with compound **4c** in a concentration-dependent manner, while having no effect on the percentage of necrotic cells. The late apoptotic ratio of cells increased to approximately 1.7% after 48 h of treatment with compound **4c** at 10 μM, while the highest rate of early apoptosis was 15.3% when the cells were treated with this compound for a similar duration and concentration (Table 6). Figure 2 displays representative cytograms of HT-29 and HepG2 cells in the absence or presence of compounds **4c** and **4e** at 48 h, as determined by this assay.

**Table 4:** Percentage of HT-29 cells in each state after treatment with compound **4c** at 48 h

Concentration	Vital (%) An −/PI −	Early apoptosis (%) An +/PI −	Late apoptosis (%) An +/PI +	Necrosis (%) An −/PI +
6 µM	92.25 ± 0.79	3.49 ± 0.53***	1.52 ± 0.02****	1.19 ± 0.11
10 µM	92.20 ± 1.64	3.96 ± 0.43***	1.53 ± 0.18****	1.64 ± 0.095
15 µM	90.26 ± 0.98	5.78 ± 0.46****	2.46 ± 0.13****	1.93 ± 0.23
Control/vehicle	97.98 ± 0.1	0.08 ± 0.99	0.29 ± 0.02	0.58 ± 0.03

The data presented are the mean ± SEM of three independent experiments. ***p < 0.001, ****p < 0.001 compared with control/vehicle

**Table 5:** Percentage of HT-29 cells in each state after treatment with compound **4e** at 48 h

Concentration	Vital (%) An −/PI −	Early apoptosis (%) An +/PI −	Late apoptosis (%) An +/PI +	Necrosis (%) An −/PI +
6 µM	97.18 ± 0.22	1.64 ± 0.16	0.35 ± 0.01	0.61 ± 0.0
10 µM	96.75 ± 0.18*	2.46 ± 0.26**	0.36 ± 0.04	0.24 ± 0.01
15 µM	95.99 ± 0.56***	3.82 ± 0.51****	0.41 ± 0.03*	0.28 ± 0.05
Control/vehicle	97.98 ± 0.1	0.99 ± 0.08	0.29 ± 0.02	0.58 ± 0.03

The data presented are the mean ± SEM of three independent experiments. *p < 0.05, **p < 0.01, ***p < 0.001and ****p < 0.0001 compared with control/vehicle

**Table 6:** Percentage of HepG2 cells in each state after treatment with compound **4c** at 48 h

Concentration	Vital (%) An −/PI −	Early apoptosis (%) An +/PI −	Late apoptosis (%) An +/PI +	Necrosis (%) An −/PI +
2 µM	92.67 ± 1.94	4.93 ± 1.75	0.95 ± 0.28	1.45 ± 0.10
5 µM	85.05 ± 1.71*	11.60 ± 1.51*	1.83 ± 0. 03*	1.31 ± 0.05
10 µM	81.77 ± 0.85**	15.37 ± 0.61**	1.71 ± 0.16*	1.14 ± 0.18
Control/vehicle	93.05 ± 0.80	4.79 ± 0.88	0.86 ± 0.09	1.30 ± 0.18

The data presented are the mean ± SEM of three independent experiments. *p < 0.05, **p < 0.01 compared with control/vehicle

### Investigating cell cycle distribution following treatment with compounds 4c and 4e

Cell proliferation and apoptosis are associated with cell cycle progression [50]. To investigate the effect of compounds **4c** and **4e** on the distribution of HT-29 and HepG2 cells in different phases of the cell cycle, flow cytometry was applied. The data on cell cycle distribution, presented in Tables 7 and 8, indicate that the ratio of HT-29 cells in various phases remained constant in both the control and the cells treated with either compound at different concentrations. In contrast, when HepG2 cells were treated with 10 µM concentration of compound **4c**, a significant increase in the sub-G1 phase cell population at the expense of reduced G1, S and G2/M phases populations was observed compared to the control (Table 9). Together, these results along with the annexin/PI findings confirm an apoptosis induction by compound **4c** in HepG2 cells with no cell cycle arrest.

**Table 7:** Effect of compound **4c** at different concentrations on HT-29 cell cycle progression after 48 h of incubation

Concentration	HepG2			
	Sub-G1	G_0_/G_1_	S	G_2_/M
6 µM	47.79 ± 0.75	29.23 ± 2.18	21.67 ± 0.32	1.65 ± 0.11
10 µM	48.46 ± 2.16	31.65 ± 2.78	20.63 ± 0.45	4.56 ± 1.12
15 µM	47.50 ± 1.48	27.50 ± 1.63	22.3 ± 0.27	1.31 ± 0.02
Control/vehicle	42.65 ± 1.61	31.16 ± 0.89	21.77 ± 0.39	4.40 ± 0.36

The data presented are the mean ± SE of three independent experiments

**Table 8:** Effect of compound **4e** at different concentrations on HT-29 cell cycle progression after 48 h of incubation

Concentration	HepG2			
	Sub-G1	G_0_/G_1_	S	G_2_/M
2 µM	45.77 ± 2.45	30.68 ± 1.89	20.93 ± 0.18	4.40 ± 0.64
5 µM	47.10 ± 2.12	29.00 ± 0.41	21.10 ± 0.94	4.47 ± 0.44
10 µM	47.17 ± 2.40	32.16 ± 1.55	18.7 ± 0.12	3.98 ± 0.36
Control/vehicle	42.65 ± 1.61	31.16 ± 0.89	21.77 ± 0.39	4.40 ± 0.36

The data presented are the mean ± SE of three independent experiments

**Table 9:** Effect of compound **4c** at different concentrations on HepG2 cell cycle progression after 48 h of incubation

Concentration	HepG2			
	Sub-G1	G_0_/G_1_	S	G_2_/M
2 µM	29.58 ± 1.66	31.00 ± 1.74	26.75 ± 0.94	9.75 ± 0.77
5 µM	39.55 ± 1.28**	27.77 ± 1.06	25.04 ± 0.43	8.9 ± 1.15
10 µM	42.99 ± 1.09***	24.69 ± 1.14**	24.13 ± 0.66*	8.23 ± 0.89*
Control/vehicle	32.79 ± 0.66	32.00 ± 1.01	27.49 ± 0.58	10.98 ± 0.18

The data presented are the mean ± SEM of three independent experiments *p < 0.05, **p < 0.01, ***p < 0.001 compared with control/vehicle

### Influence of compound 4c on cell morphology alteration and caspase-3 cleavage

**Fig. 3:**
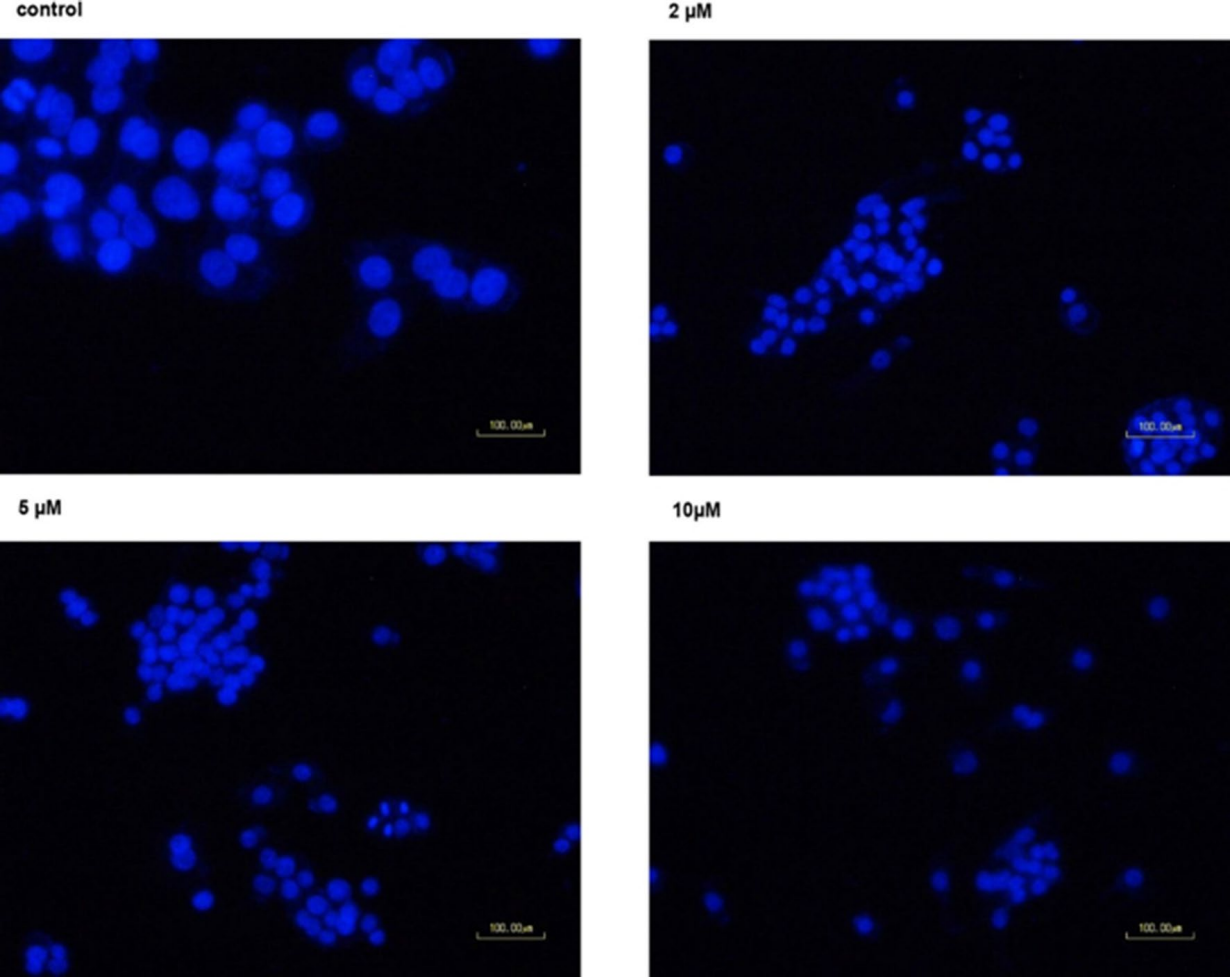
DAPI staining was used to stain nuclei in HepG2 cells treated with compound **4c** at different concentrations for 48 h. The morphological changes were observed using a fluorescent microscope (20X)

Based on the annexin/PI and cell cycle findings, apoptosis induction by compound **4c** in HepG2 cells was further verified by DAPI staining. To do this, HepG2 cells were treated with different concentrations (2, 5, 10 µM) of compound **4c**. As shown in Fig. 3, 5 and 10 µM of compound **4c** treatment resulted in nuclei with bright, condensed, horse-shoe shaped and fragmented appearance, whereas the nuclear structure of control cells and cells treated with 2 µM of the compound remained intact displaying a homogenous weak blue stain.

**Fig. 4:**
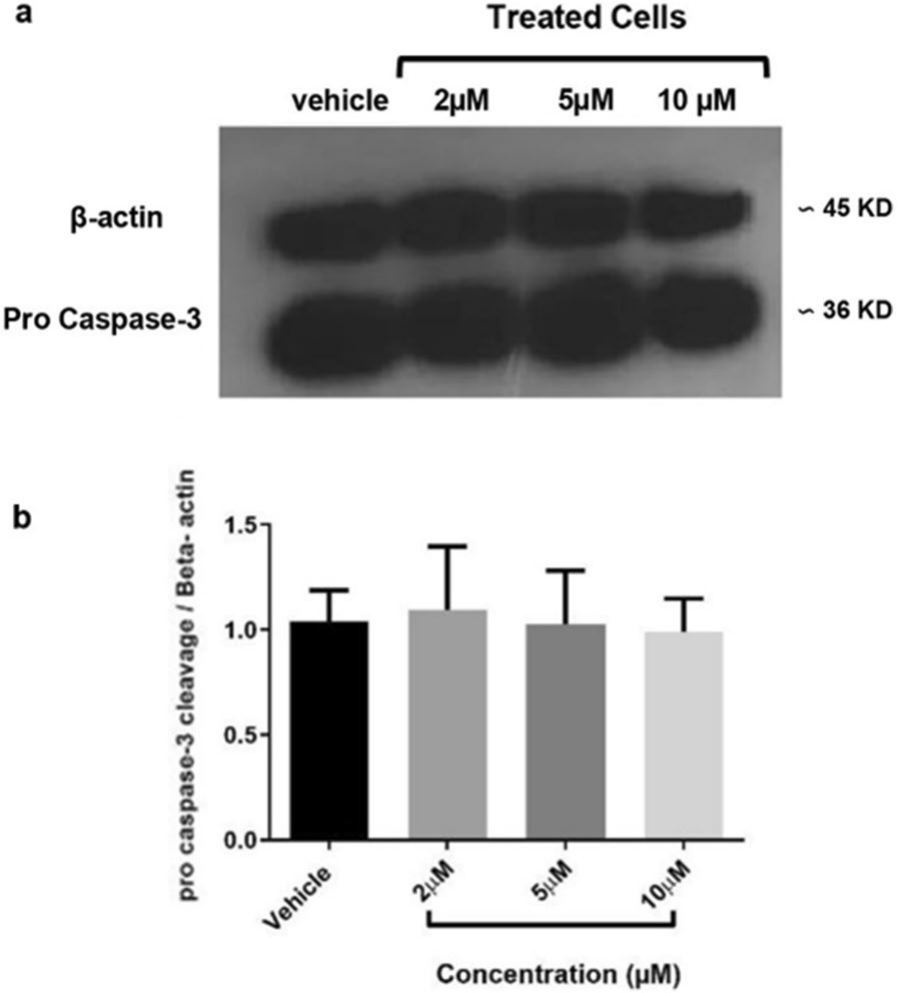
Procaspase-3 expression in HepG2 cells treated with DMSO or 2, 5 and 10 μM concentrations of compound **4c** for 48 h. β-actin was used as internal control. **a** The corresponding protein levels in the cropped gel were assessed using densitometry. Samples derived from the same experiment were run in two different gels simultaneously, and were processed in parallel. **b** Relative expression of procaspase-3 to β-actin was measured. Each value represents the mean ± SEM of three independent experiments. One-way ANOVA analysis with Tukey post test was performed

Considering the important role played by members of the caspase family in certain apoptotic processes, changes in their expression was also investigated. Among them, caspase-3 is known for catalyzing specific cleavage of the key cellular proteins [51]. Herein, the cleavage of procaspase-3 was examined by Western blot analysis. There was no difference in terms of procaspase-3 expression between control and the HepG2-treated groups suggesting a caspase-3 independent apoptosis induction (Fig. 4).

## Discussion

Sorafenib with bis-aryl urea structure has been widely used alone or in combination with other drugs in treating certain types of cancer including hepatocellular carcinoma as it induces apoptosis and inhibits angiogenesis. However, toxicity of sorafenib can severely influence the well-being of patients [52, 53]. In addition, in the HT-29 colon tumors, the anti-angiogenic and anti-proliferative properties of sorafenib have also been demonstrated [54, 55]. On the other hand, hydrazide-hydrazones have shown a remarkable antiproliferative activity against a panel of cancer cells including liver and colon cancers [56]. These findings motivated us to direct our efforts towards developing potent anti-cancer compounds by hybridization of the two pharmacophores urea and hydrazide.

Structures of the synthesized hybrid molecules were determined by spectral analysis data and the purity of the derivatives was confirmed according to the CHN analysis results. In the FT-IR spectra, the NH moiety was detected as one or two signals between 3360 and 3220 cm^−1^, while the C=O moiety showed up in the range of 1620–1690 cm^−1^. In proton NMR, the imine bond formation was confirmed following the detection of a peak close to 8.38–8.85 ppm and the chemical shift of the mentioned imine hydrogen verified the formation of only the *E* isomer. The NH proton is exchangeable and the related signal is usually broad, though it may disappear in certain situations including at low concentrations. Missing protons detected in ^1^HNMR spectra of the compounds were assigned to NH, although NH absorbance was detected in FT-IR spectra.

In the carbon 13 spectra, two carbonyl groups and an imine carbon were defined over 160, 150 and 145 ppm. The number of signals for compounds **4a**, **4f**, and **4 g** was less than the predicted peaks; for instance, of the expected 17 signals for compound **4a**, only 16 were observed, which is likely due to the rare overlap of the two different carbons or the exclusion of weak signals at low concentrations. For **4a,** the chemical shifts of C_2_ and C_6_ or C_3′_, C_5′_ and C_2″_, C_6″_ were close to each other, respectively, and overlapping may have occurred. In **4f**, this similarity exists between C_2_ and C_3′_, _5′_. However, the same chemical shifts of C_2_ and C_3′_, C_5′_ or C_5_ and C_2′_, C_6′_ would likely be expected for 4** g**. Since the molecular ion of certain compounds is intrinsically unstable and quickly breaks into fragments, it cannot be detected in mass spectrometry. Similarly, compounds with urea moieties showed no significant peaks [57]. In the present study, the low abundance of the molecular ions may have also been due to their instability; however, the fragmentation pattern is acceptable and the structures were confirmed by FT-IR, ^1^HNMR and ^13^CNMR.

The synthesized compounds were screened for their cytotoxic activity against the two human cancer cell lines HT-29 (human colorectal adenocarcinoma) and HepG2 (human hepatocellular carcinoma) using the MTT assay at 72 h. Among the tested compounds, cytotoxicity of **4c** and **4e** was pronounced against both cell lines after 72 h of incubation. Compound **4e** was cytotoxic against both HT-29 and HepG2 cell lines following 72 h of treatment with IC_50_ values of 3.40 and 4.82 µM, respectively. Compound **4c,** however**,** showed a more desirable cytotoxic effect than **4e** in particular against HepG2 cells at 72 h with an IC_50_ value of 2.22 µM. To further verify the critical role of *p*-chlorophenyl urea in the anti-proliferative activity of the structures, several compounds bearing phenyl urea (**4a**), phenyl thiourea (**4b**), *p*-chlorophenyl urea (**4c**), and *p*-toluene sulfonyl urea (**4d**) were synthesized. The cytotoxicity screening identified compound **4c,** bearing *p*-chlorophenyl urea, as being more potent than others. Furthermore, the urea moiety was superior to thiourea in its cytotoxic effect. Moreover, in line with other studies showing the beneficial effect of electron withdrawing groups at the *meta* position of the phenyl ring close to hydrazone on anticancer activity [42, 43], a comparative analysis of compounds **4a** and **4f** was also performed. The analysis revealed that by replacing the *nitro* moiety with a *Cl* group on the phenyl ring of hydrazone, the change in cytotoxic activity becomes insignificant. Finally, the positional effect of the *chloro* substituent, one of several electron withdrawing substituents on the hydrazone phenyl ring, was also investigated. Our results suggested that the spatial position of the *Cl* group has a great impact on the properties, as exemplified by that of compound **4c** versus **4h**. Given these data, compounds **4c** and **4e** were selected for further analysis. It was also interesting to note that the latter two compounds displayed minimal growth inhibitory effect on normal cells. The IC_50_ values revealed that the selectivity for compound **4c** is greater than compound **4e** towards liver cancer cells over normal cells.

Considering the IC_50_ values determined for compounds **4c** and **4e** on the tested cell lines, the next step was to determine the underlying mechanisms affected by the two compounds in HT-29 and by compound **4c** in HepG2 cells. With apoptosis being a crucial mechanism of action of anticancer agents [4], several approaches were taken and demonstrated that the mechanism of colon and liver cancer cell growth inhibition is indeed through apoptosis. Following the addition of Annexin V, surface exposure of phosphatidylserine by apoptotic cells was determined. PI has been applied to recognize dead or late apoptotic cells due to the permeability of the membranes of the damaged and dead cells to PI [58]. We noticed that treatment of HT-29 cells with compound **4e** could significantly increase apoptosis at higher concentrations. However, compound **4c** was stronger in terms of apoptosis induction in this cell line after 48 h of incubation. Similarly, the results indicated that the percentage of apoptotic HepG2 cells treated with compound **4c** at 48 h was much higher than that of HT-29 cells, indicating that HepG2 cells might respond to compound **4c** differently. Likewise, the accumulation of the sub G1 population in the cell cycle phase analysis is regarded as the best marker of apoptosis due to a reduced DNA content in apoptotic cells [59]. Besides, loss of S phase population is another important indicator of apoptosis [60]. In this context, our findings implied an increase in the sub G1 population and a decrease in the S phase population of the HepG2 cells upon treatment with compound **4c** at 5 and 10 µM, further confirming a greater ability of this compound in apoptosis induction in cancerous hepatocytes.

In addition to the phosphatidylserine translocation, programmed cell death (apoptosis) is characterized by certain morphological features including chromatin condensation and nuclear deformation, which were observed following treatment with 5,10 µM of compound **4c** in HepG2 cells. Moreover, activation of caspase-3, an enzyme involved in apoptosis, has also been reported following treatment with several anticancer agents [61, 62]. Indeed, activation of caspase-3 is the most frequently observed feature in several cell types undergoing apoptosis. In this regard, compound **4c** at three different concentrations did not alter the cleavage of caspase-3 in the treated cells relative to the untreated cells, suggesting the involvement of a caspase-3 independent pathway. It has been reported that caspase-independent cell death is likely through the activation of AIF (apoptosis inducing factor) and ROS, providing further options to fight cancer cells [63, 64].

## Conclusions

In summary, compounds **4c** and **4e** as hybrids of ureido and hydrazone pharmacophores were synthesized, characterized and investigated for the first time in relation to human colon adenocarcinoma and hepatocellular carcinoma. Notably, these synthesized compounds displayed a concentration-dependent cytotoxicity towards the two tested cancer cells, making it likely to be an efficacious chemotherapeutic agent. Moreover, our data showed that compound **4c** was able to significantly inhibit cancer cell growth via apoptosis induction which is not mediated through cell cycle arrest. Taken together, the current work presented compound **4c** as a potential lead compound in developing future hepatocellular carcinoma chemotherapy drugs.

## Materials and methods

### Chemicals and reagents

Chemicals were purchased from Merck chemical company (Darmstadt, Germany). TLC was applied to detect end of the reactions of the synthesized compounds. Melting points were determined in open capillary tubes and presented uncorrected. Structures of the target compounds (**4a–4i**) were confirmed by FT-IR, ^1^H-NMR, ^13^C-NMR and Mass spectra. FT-IR spectra were recorded on Shimadzu FT-IR-8400 instrument in KBr disk. ^1^H-NMR spectra were recorded on Bruker AC-500 MHz FT-NMR using DMSO-d6. Mass spectra were recorded on Jeol-D300 spectrometer. Elemental analyses were carried out with a Perkin-Elmer Model 240-c apparatus (Perkin Elmer, Norwalk, CT, USA), and the results of the elemental analyses (C, H, N) were obtained within ± 0.4% of the calculated amounts.

### Chemistry

The target compounds (**4a–4i**) were synthesized according to Scheme 2. In details, reaction of ester **1** with hydrazine hydrate gave hydrazide **2** as described previously [57, 65]. Hydrazones **3a**–**3d** were obtained through acid- catalyzed condensation of **2** with the corresponding aromatic aldehydes [66–70]. The final compounds **4a**–**4i** were prepared by treatment of **3a**–**3d** with different aromatic isocyanates or phenyl isothiocyanate [57]. Synthesis and anti-bacterial activity of compound **4b** have been reported earlier [66].

### General procedure for synthesis of the target compounds (4a–4i)

A mixture of hydrazones **3a**–**3d** (0.75 mmol) and the corresponding isocyanates and isothiocyanates (3 mmol) in acetone (5 ml) along with a few drops of DMF was stirred under reflux for 1 h. End of the reaction was observed by TLC, water was then added to the mixture and cooled to 0 °C. The precipitate was dissolved in ethyl acetate and washed with water. The organic phase was then dried by anhydrous sodium sulfate, evaporated and the precipitate was finally recrystallized from isopropanol.

#### 1-(4-(2-(3-chlorobenzylidene)hydrazine-1-carbonyl)phenyl)-3-phenylurea (4a)

Yield: 75%; mp: 251–252 °C; IR (KBr) ν cm^−1^: 3327, 3284 (NH), 1648 (C=O); ^1^H NMR (500 MHz, DMSO-d_6_): *δ* 8.65 (s, 1H, CH=N), 7.55 (d, *J* = 8.0 Hz, 2H, H_2′_, H_6′_), 7.46 (d, *J* = 8.0 Hz, 2H, H_3′_, H_5′_), 7.28 (q, *J* = 8.1 Hz,4H, H_2_, H_6_, H_2″_, H_6″_), 7.14–7.09 (m, 4H, H_3″_, H_5″_, H_4_, H_5_), 6.97 (t, *J* = 7.9 Hz, 1H, H_4″_); ^13^C NMR (125 MHz, DMSO-d_6_): *δ* 164.89, 154.14, 146.37, 140.91, 139.17, 134.36, 133.65, 131.20, 129.70, 129.13, 128.92, 127.92, 125.70, 123.00, 119.11, 118.63; Mass: m/z (%): 394 (M^+^ + 2, 2), 392 (M^+^, 6), 391.1 (16), 169.1 (23), 146.1 (47), 120.1 (64.5), 91.1 (44), 77 (28.7), 43.1 (100); Anal. Calcd. for C_21_H_17_ClN_4_O_2_: C, 64.21; H, 4.36 N, 14.26. Found: C, 63.97; H, 4.29; N, 14.12.

#### 1-(4-(2-(3-chlorobenzylidene)hydrazine-1-carbonyl)phenyl)-3-phenylthiourea (4b)

Yield: 75%; mp: 266–267 °C; IR (KBr) ν cm^−1^: 3320 (NH), 1648 (C=O); ^1^H NMR (500 MHz, DMSO-d_6_): *δ* 8.85 (bs, 1H, NH), 8.57 (s, 1H, CH=N), 8.01 (bs, 1H, NH), 8.66 (d, *J* = 8.5 Hz, 2H, H_2′_, H_6′_), 7.70–7.78 (m, 6H, H_2_, H_4_, H_5_, H_6_, H_3′_, H_5′_), 7,52–7.54 (m, 2H, H_3″_, H_5″_), 7.43–7.46 (m, 3H, H_2″_, H_4″_, H_6″_); ^13^C NMR (125 MHz, DMSO-d_6_): *δ* 179.41, 165.36, 146.52, 140.28, 138.49, 134.44, 133.71, 132.10, 131.02, 129.73, 128.92, 128.39, 127.79, 125.60, 124.18, 123.70, 122.10; Mass: m/z (%): 408 (M^+^, 8), 120 (92%), 97 (100%), 75 (57%), 65 (60%); Anal. Calcd. for C_21_H_17_ClN_4_OS: C, 61.68; H, 4.19; N, 13.70. Found: C, 61.49; H, 4.09; N, 13.87.

#### 1-(4-(2-(3-chlorobenzylidene)hydrazine-1-carbonyl)phenyl)-3-(4-chlorophenyl)urea (4c)

Yield: 35%; mp: 255.5–257 °C; IR (KBr) ν cm^−1^: 3298 (NH), 1633 (C=O); ^1^H NMR (500 MHz, DMSO-d_6_): *δ* 8.79 (s, 1H, CH=N), 8.03 (d, *J* = 9.2 Hz, 2H, H_2′_, H_6′_), 7.96 (d, *J* = 8.6 Hz, 2H, H_3′_, H_5′_), 7.81 (s, 1H, H_2_), 7.75 (t, *J* = 6.9 Hz, 3H, H_4_, H_5_, H_6_), 7.56 (d, *J* = 8.4 Hz, 2H, H_2″_, H_6″_), 7.28 (d, *J* = 8.4 Hz, 2H, H_3″_, H_5″_); ^13^C NMR (125 MHz, DMSO-d_6_): *δ* 165.39, 154.06, 146.55, 141.13, 139.79, 135.52, 134.44, 133.91, 131.08, 129.24, 129.37, 128.92, 127.77, 127.70, 125.26, 120.33, 118.62; Mass: m/z (%):427 (M^+^ + 1, 7.9), 126 (22.65), 107 (76.56), 97 (100), 77 (52.35), 53 (54.7); Anal. Calcd. for C_21_H_16_Cl_2_N_4_O_2_: C, 59.03; H, 3.77; N, 13.11. Found: C, 59.32; H, 3.68; N, 13.19.

#### N-((4-(2-(3-chlorobenzylidene)hydrazine-1-carbonyl)phenyl)carbamoyl)-4-methylbenzenesulfonamide (4d)

Yield: 90%; mp: 227–229 °C; IR (KBr) ν cm^−1^: 3221 (NH), 1632 (C=O), 1340, 1147 (SO_2_); ^1^H NMR (500 MHz, DMSO-d_6_): *δ* 8.38 (s, 1H, CH=N), 7.83–7.79 (m, 3H, H_2,_ H_2′_, H_6′_), 7.70–7.66 (m, 4H, H_5_, H_6,_ H_3′_, H_5′_),7.47 (d, *J* = 8.5 Hz, 1H, H_4_), 7.43 (d, *J* = 8.2 Hz, 2H, H_2″_, H_6″_), 7.40 (d, *J* = 8.2 Hz, 2H, H_3″_, H_5″_), 2.35 (s, 3H, CH_3_); ^13^C NMR (125 MHz, DMSO-d_6_): *δ* 165.33, 150.26, 146.00, 142.86, 141.28, 137.20, 134.52, 133.26, 131.11, 130.98, 129.97, 129.47, 129.21, 128.91, 127.54, 125.46, 118.75, 21.54; Mass: m/z (%): 470 (M^+^, 7), 197 (50), 171 (48), 155 (80), 91 (100); Anal. Calcd. for C_22_H_19_ClN_4_O_4_S: C, 56.11; H, 4.07; N, 11.90. Found: C, 56.43; H, 4.14; N, 11.84.

#### 1-(4-chlorophenyl)-3-(4-(2-(3-nitrobenzylidene)hydrazine-1-carbonyl)phenyl)urea (4e)

Yield: 65%; mp: 247.5–249 °C; IR (KBr) ν cm^−1^: 3293 (NH), 1660 (C=O), 1525, 1346 (NO_2_); ^1^H NMR (500 MHz, DMSO-d_6_): *δ* 8.85 (s, 1H, CH=N), 8.30 (bs, 2H, H_2_, H_4_), 8.07–8.25 (m, 3H, H_2′_, H_6′_, H_6_), 7.46–7.49 (m, 4H, H_2″_, H_6″_, H_3′_, H_5′_), 7.32–7.34 (m, 3H, H_5_, H_3″_, H_5″_); ^13^C NMR (125 MHz, DMSO-d_6_): *δ*; 165.35, 154.20, 146.40, 144.46, 141.13, 139.79, 137.65, 134.46, 131.08, 129.92, 129.55, 128.96, 126.63, 124.22, 122.17, 120.27, 118.69; Mass: m/z (%): 437.4 (M^+^, 7.3), 280.1 (43), 153.1 (25), 127.2 (100), 111.5 (19), 99.1 (35), 43.2 (17); Anal. Calcd. for C_21_H_16_ Cl N_5_O_4_: C, 57.61; H, 3.68; N, 16.00. Found: C, 57.92; H, 3.52; N, 16.12.

#### 1-(4-(2-(3-nitrobenzylidene)hydrazine-1-carbonyl)phenyl)-3-phenylurea (4f)

Yield: 87%; mp: 258–259 °C; IR (KBr) ν cm^−1^: 3320, 3289 (NH), 1649 (C = O), 1511, 1313 (NO_2_); ^1^H NMR (500 MHz, DMSO-d_6_): *δ* 8.67 (s, 1H, CH = N), 8.09 (m, 2H, H_2_, H_4_), 7.75 (t, *J* = 9.7 Hz, 3H, H_6_, H_2′_, H_6′_), 7.49 (t, *J* = 7.3 Hz, *J* = 8.1 Hz, 3H, H_5_, H_3′_, H_5′_), 7.34 (t, *J* = 7.5 Hz, 4H, H_2″_, H_3″_, H_5″_, H_6″_), 7.13 (t, *J* = 7.3 Hz, 1H, H_4″_); ^13^C NMR (125 MHz, DMSO-d_6_): 164.87, 154.15, 145.73, 144.37, 141.06, 139.26, 134.20, 131.76, 129.66, 129.58, 129.14, 128.36, 123.02, 122.65, 119.02, 118.84; Mass: m/z (%): 403 (M^+^, 5.7), 228 (17.9), 135 (100), 93 (99.1), 77 (69.1); Anal. Calcd. for C_21_H_17_N_5_O_4_: C, 62.53; H, 4.25; N, 17.36. Found: C, 62.31; H, 4.29; N, 17.22.

#### 4-methyl-N-((4-(2-(3-nitrobenzylidene)hydrazine-1-carbonyl)phenyl)carbamoyl)benzenesulfonamide (4 g)

Yield: 90%; mp: 245–247 °C; IR (KBr) ν cm^−1^: 3357, 3260 (NH), 1623 (C=O), 1494, 1339 (NO_2_), 1295, 1148 (SO_2_); ^1^H NMR (500 MHz, DMSO-d_6_): *δ* 8.85 (s, 1H, CH=N), 8.19 (d, *J* = 8.9 Hz, 2H, H_2_, H_4_), 7.85 (d, *J* = 5.8 Hz, 3H, H_6_, H_2′_, H_6′_), 7.71 (d, *J* = 8.2 Hz, 3H, H_5_, H_3′_, H_5′_), 7.64 (d, *J* = 8.2 Hz, 2H, H_2″′_, H_6″_), 7.32 (d, *J* = 8.1 Hz, 2H, H_3″_, H_5″_), 2.36 (s, 1H, CH_3_); ^13^C NMR (125 MHz, DMSO-d_6_) 165.37, 150.26, 146.35, 144.38, 142.68, 141.28, 137.10, 134.61, 129.95, 129.65, 129.21, 128.53, 127.68, 122.15, 118.74, 21.38; Mass: m/z (%): 481.6 (M^+^, 5), 226.2 (46), 150.1 (38), 120.2 (38), 91.2 (100), 71.2 (92); Anal. Calcd. for C_22_H_19_N_5_O_6_S: C, 54.88; H, 3.98; N, 14.55. Found: C, 54.72; H, 3.86; N, 14.73.

#### 1-(4-(2-(4-chlorobenzylidene)hydrazine-1-carbonyl)phenyl)-3-(4-chlorophenyl)urea (4 h)

Yield: 84%; mp: 256.5–257 °C; IR (KBr) ν  cm^−1^: 3290 (NH), 1628 (C=O), ^1^H NMR (500 MHz, DMSO-d_6_): *δ* 8.67 (s, 1H, CH=N), 8.22 (d, *J* = 7.5 Hz, 2H, H_2′_, H_6′_), 7.91 (d, *J* = 8.5 Hz, 2H, H_3′_, H_5′_), 7.80 (d, *J* = 7.6 Hz, 2H, H_2,_ H_6_), 7.55 (d, *J* = 8.1 Hz, 2H, H_2″_, H_6″_), 7.45 (d, J = 8.1 Hz, 2H, H_3_, H_5_), 7.28 (d, *J* = 8.3 Hz, 2H, H_3″_, H_5″_); ^13^C NMR (125 MHz, DMSO-d_6_): *δ* 164.88, 154.19, 146.03, 140.91, 139.17, 137.74, 132.71, 131.88, 129.77, 129.12, 128.78, 127.26, 125.97, 119.35, 118.63; Mass: m/z (%): 426.5 (M^+^, 10), 280.1 (25%), 243.2 (12%), 222 (9%), 195.1 (20%), 154 (15%), 127.1 (100%), 111 (11%), 103 (21%); Anal. Calcd. for C_21_H_16_Cl_2_N_4_O_2_: C, 64.21; H, 4.36; N, 14.26. Found: C, 64.01; H, 4.29; N, 14.34.

#### 4-methyl-N-((4-(2-(pyridin-4-ylmethylene)hydrazine-1-carbonyl)phenyl)carbamoyl)benzenesulfonamide (4i)

Yield: 76%; mp: 259–261 °C; IR (KBr) ν cm^−1^: 3275 (NH), 1689 (C=O), 1301, 1150 (SO_2_); ^1^H NMR (500 MHz, DMSO-d_6_): *δ* 8.79 (s, 1H, CH=N), 8.66 (d, *J* = 5.5 Hz, 2H, H3, H5), 8.58 (d, *J* = 5.5 Hz, 2H, H_2_, H_6_), 8.02–7.92(m, 4H, H_2′_, H_3′_, H_5′_, H_6′_), 7.77 (d, *J* = 8.2 Hz, 2H, H_2″_, H_6″_), 7.26 (d, *J* = 8.0 Hz, 2H, H_3″_, H_5″_), 2.31(S, 3H, CH_3_); ^13^C NMR (125 MHz, DMSO-d_6_): *δ* 163.32*,* 150.28, 149.79, 148.13, 146.27, 142.63, 141.22, 137.20, 132.24, 131.04, 129.67, 127.65, 123.84, 118.71, 21.52; Mass: m/z (%): 437.2 (M^+^, 7.1), 339.4 (32.9), 313.4 (66.4), 262.3 (77.1), 239 (45), 135 (25), 109 (46.4), 83 (78.5), 57 (100); Anal. Calcd. for C_21_H_19_N_5_O_4_S: C, 57.66; H, 4.38; N, 16.01. Found: C, 57.34; H, 4.47; N, 16.12.

### Cell lines and reagents

Human colorectal adenocarcinoma cell line HT-29, human hepatocarcinoma cell line HepG2, and mouse embryonic fibroblasts (NIH3T3) were provided from the National Cell Bank of Pasture Institute of Iran (NCBI) and Iranian Biological Resource Center (IBRC). Dulbecco's modified eagle’s medium (DMEM), DMED/F12, FBS (Fetal Bovine Serum), trypsin–EDTA, and penicillin G/streptomycin were purchased from Gibco TM (Gibco-BRL, Rockville, IN, USA). The 3-(4,5-dimethylthiazol-2-yl) 2,5-diphenyltetrazolium (MTT) was obtained from Sigma‑Aldrich (Saint Louis, Missouri, USA). Other chemicals were supplied by Merck (Darmstadt, Germany) and Sigma-Aldrich (St Louis, MO, USA).

### MTT assay

Synthesized compounds (**4a**–**4i**) were dissolved in DMSO (0.5%), and the cells were treated with different concentrations (1–50 μM) of the compounds. MTT assay was carried out based on the protocol described previously [71]. A total of 3–7 × 10^3^ cells/well for HT-29 and 4–8 × 10^3^cells/well for HepG2 and 1 × 10^3^ cells/well for NIH3T3 were cultured in 96-well plates and kept to be attached overnight. For a preliminary screening, cytotoxic activity of the compounds was calculated at a unique concentration (10 μM) at 72 h, and the IC_50_ values were measured for the selected compounds at 24, 48 and 72 h towards HT-29 and HepG2 cells.

### Annexin V/PI assay

The amount of apoptotic and necrotic cells were determined using an Annexin V-FLUOS apoptosis detection kit with PI (Roche Applied Science, Indianapolis, IN, USA) according to the instruction provided with the kit. A total of 3 × 10^4^ of HT-29 cells/well were seeded in 6-well plates and treated with 6, 10 and 15 µM of compounds **4c** and **4e** for 48 h, whereas HepG2 cells were treated with 2, 5 and 10 µM concentrations of compound **4c**. Cells were then incubated in the dark at 37 °C, upon addition of 5 μl of annexin V-FLUOS and PI (propidium iodide) reagents to the suspended cells. The tubes were analyzed by a flow cytometer (PARTEC GmbH, Munster, Germany) [25].

### Analysis of cell cycle progression

To investigate the influence of compounds **4c** and **4e** on cell cycle progression, PI staining was used. HT-29 cells with a density of about 3 × 10^4^ cells/well were subjected to compounds **4c** and **4e** at 6, 10, 15 µM, while HepG2 was incubated with compound **4c** at 2, 5, 10 µM for 48 h. Cells were then fixed using 70% ethanol for 24 h at − 20 °C and stained whit PI (1 mg/ml) for 15 min at 37 °C. The percentage of cell populations in G0/G1, S, and G2/M phase of the cell cycle was determined with a PARTEC flow cytometer (PARTEC GmbH, Munster, Germany) using the FlowJo software [71].

### DAPI staining

Morphological alterations in the nucleus of the treated and control cells were evaluated by DAPI staining. To do this, HepG2 cells (5 × 10^3^) were seeded into 24-well plates containing culture media overnight. Cells were then treated with 2,5 and 10 µM concentrations of compound **4c** for 48 h. Afterwards, the cells were stained by DAPI for 15 min, and the morphological changes were investigated under a fluorescence microscope (Nikone eclipse TS100).

### Western blot analysis

To determine the expression of the key protein involved in the apoptotic pathway, Western blotting was applied. HepG2 cells (3 × 10^4^/well) were incubated with 2,5 and 10 µM of compound **4c** for 48 h, and the cells were lysed using the lysis buffer [25]. The amount of protein in the cell extracts was quantified by the BCA assay [72]. Equal amounts of proteins were loaded onto a 12% sodium SDS-PAGE (dodecyl sulfate polyacrylamide gel electrophoresis), separated electrophoretically and transferred onto a PVDF membrane (GE Health Care Life Sciences, Buckinghamshire, UK) using the standard protocol. The membranes were appropriately cut considering the molecular weight of the target protein and then probed with the antibody targeting caspase-3 at 1:1000 dilution (Merck-Millipore company, Cat.No.235412) in order to save antibody. After washing the membranes, they were incubated with the secondary antibody conjugated with horseradish peroxidase at 1:8000 dilution for two hours at room temperature. β-actin was used to confirm equal loading in the wells. The blots were visualized with the ECL advance Western blotting detection kit (GE Health Care Life Sciences, Buckinghamshire, UK) to compare the amount of proteins in each lane. Protein levels were determined with ImageJ software.

### Statistical analysis

Experimental data were analyzed statistically using Graph Pad Prism 6 Software. The values were expressed as mean ± SEM of at least triplicates. Data were assessed using one-way ANOVA followed by Tukey’s multiple comparison test. A *p*-value of < 0.05 was considered to be statistically significant.

## Supplementary Information

**Additional file 1. **Spectral data including FT-IR,^1^HNMR, ^13^CNMR and Mass for compounds (**4a**–**4i**).

**Additional file 2: Table S1.** Cytotoxicity screening (%) for compounds 4a–4i following treatment at 10 μM for 72 h, towards human cancer cell lines. **Table S2.** IC50 values for cytotoxic activity of doxorubicin towards cancer cells at 72 h.

## Data Availability

All data generated or analyzed during the study are included in this manuscript and additional files. Any further data required are available from the corresponding author on reasonable request.

## Abbreviations

DAPI: 4′,6-Diamidino-2-phenylindole
DMEM: Dulbecco’s modified eagle’s medium
DMF: Dimethyl formamide
DMSO: Dimethyl sulfoxide
ECL: Enhanced chemiluminescence
EDTA: Ethylenediamine tetraacetic acid
FBS: Fetal bovine serum
FDA: Food and drug administration
FT-IR: Fourier transform infrared
IBRC: Iranian biological resource center
IC_50_: The half maximal inhibitory concentration
IR: Infrared
mp: Melting point
MTT: 3-(4,5-Dimethylthiazol-2-yl) 2,5-diphenyltetrazolium
NCBI: National cell bank of Pasture Institute of Iran
NMR: Nuclear magnetic resonance
PI: Propidium iodide
SDS-PAGE: Dodecyl sulfate polyacrylamide gel electrophoresis
SEM: Standard error of the mean
SI: Selectivity index
TLC: Thin layer chromatography
