# Supplementary material for: Design and synthesis of novel ureido and thioureido conjugated hydrazone derivatives with potent anticancer activity
Source: BMC Chem. 2022 Nov 1;16(1):81. doi: 10.1186/s13065-022-00873-3 (PMC9624014; doi:10.1186/s13065-022-00873-3)
Supplement: Supplementary file 1 — Additional file 1. Spectral data including FT-IR,1HNMR, 13CNMR and Mass for compounds (4a–4i). [file 13065_2022_873_MOESM1_ESM.doc]

**Spectral data including IR,1HNMR, 13CNMR and Mass for compounds (4a-4i)**

**(4a)**

1-(4-(2-(3-chlorobenzylidene)hydrazine-1-carbonyl)phenyl)-3-phenylurea

*
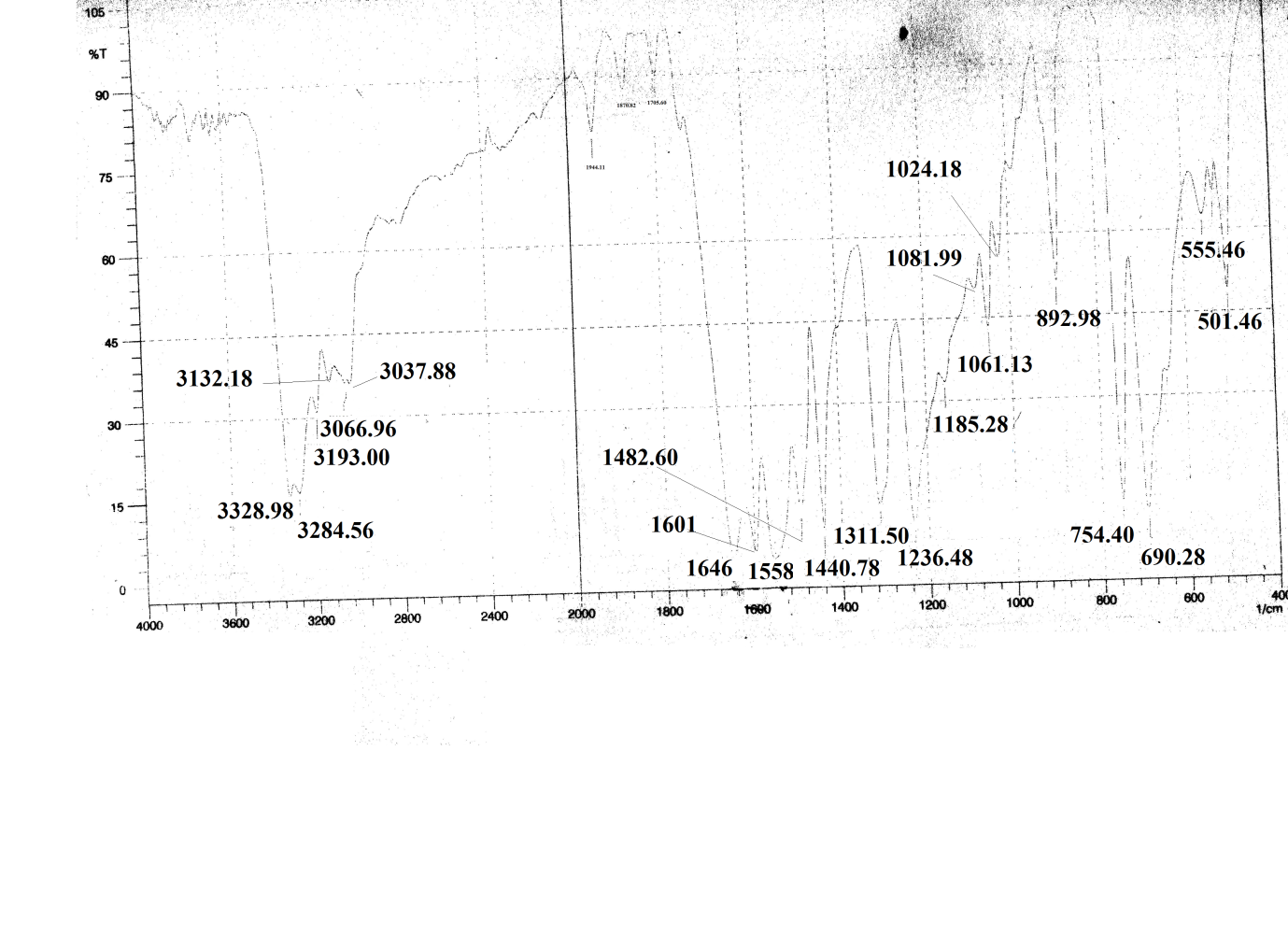
*

*
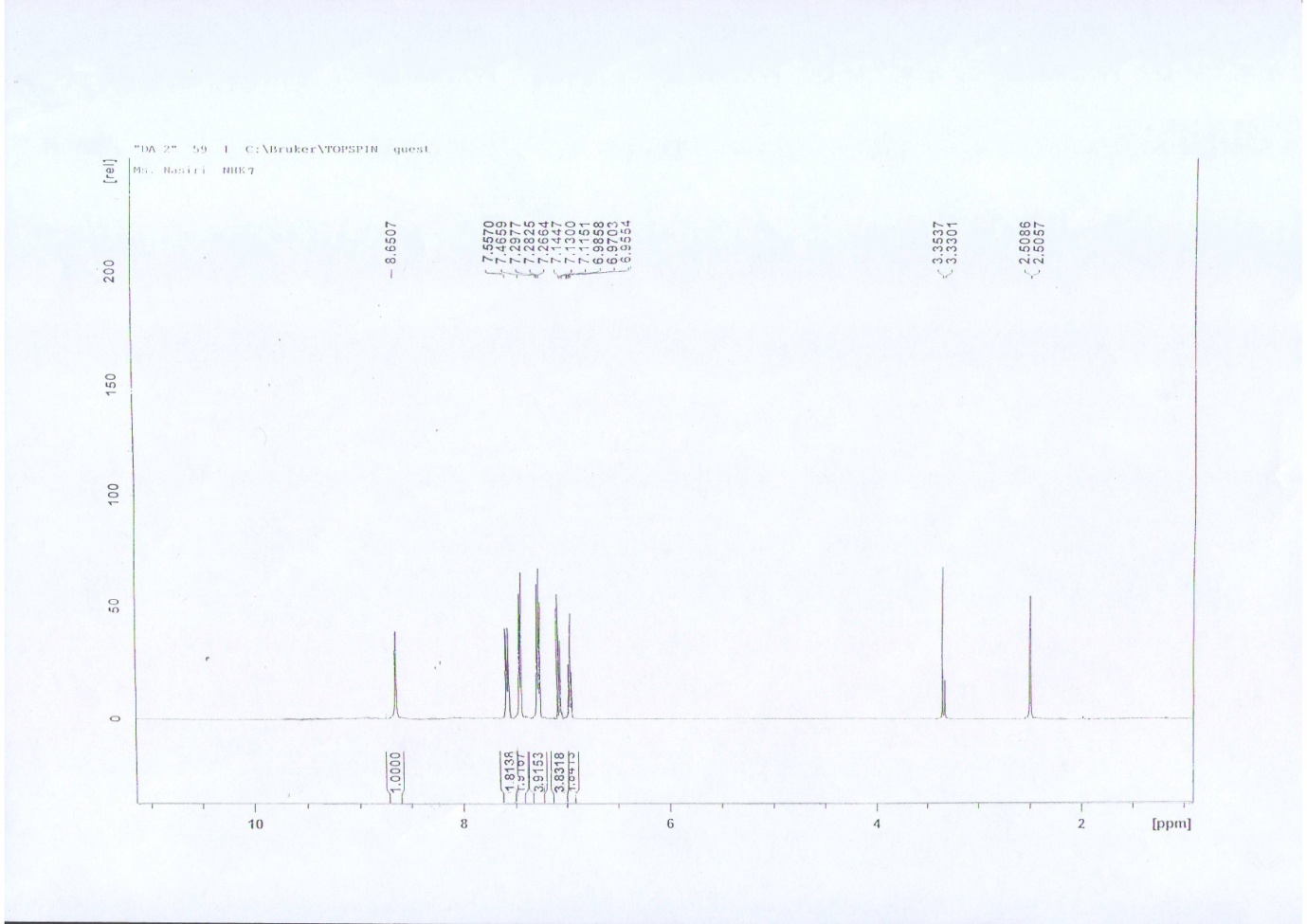
*

*
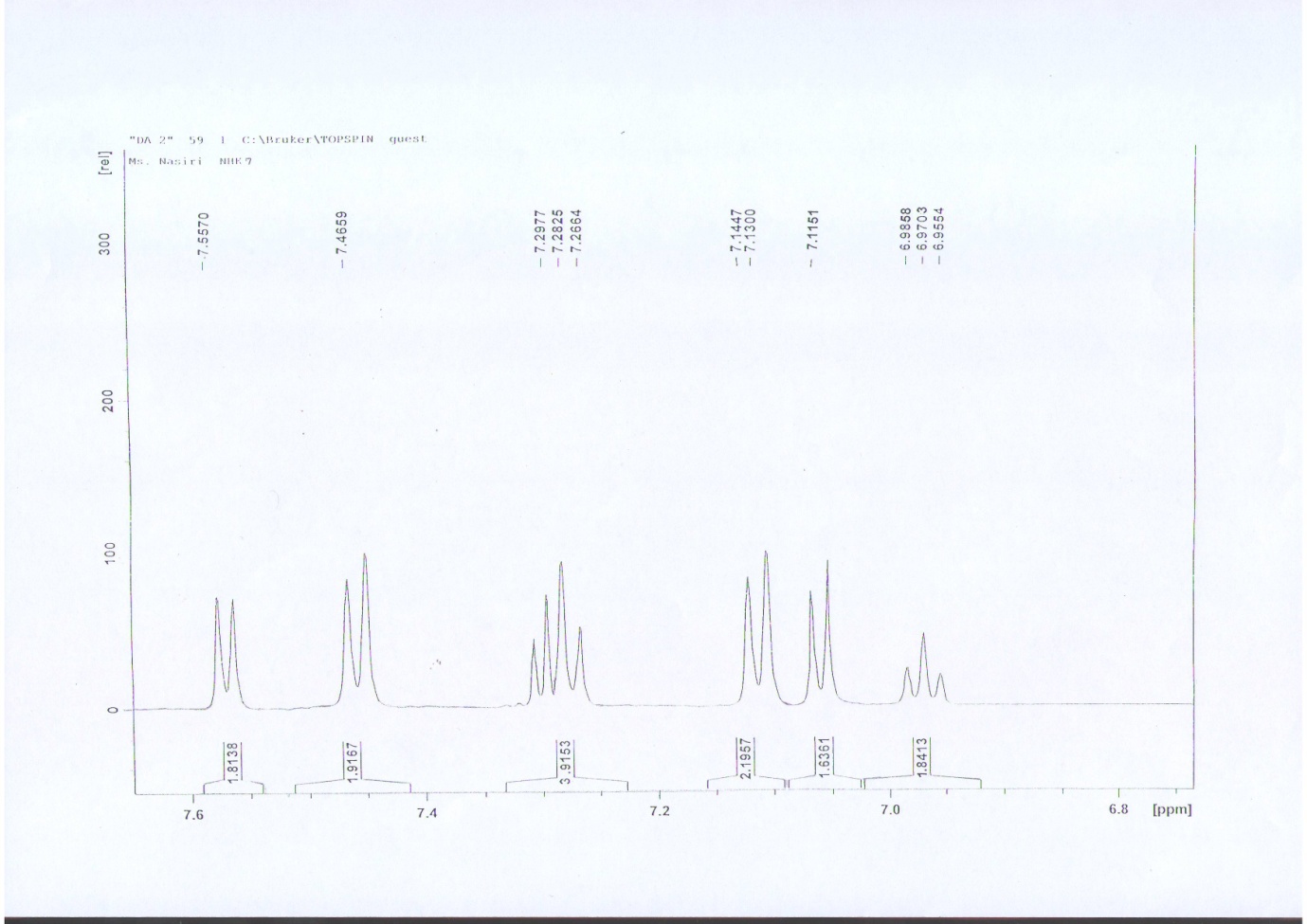
*


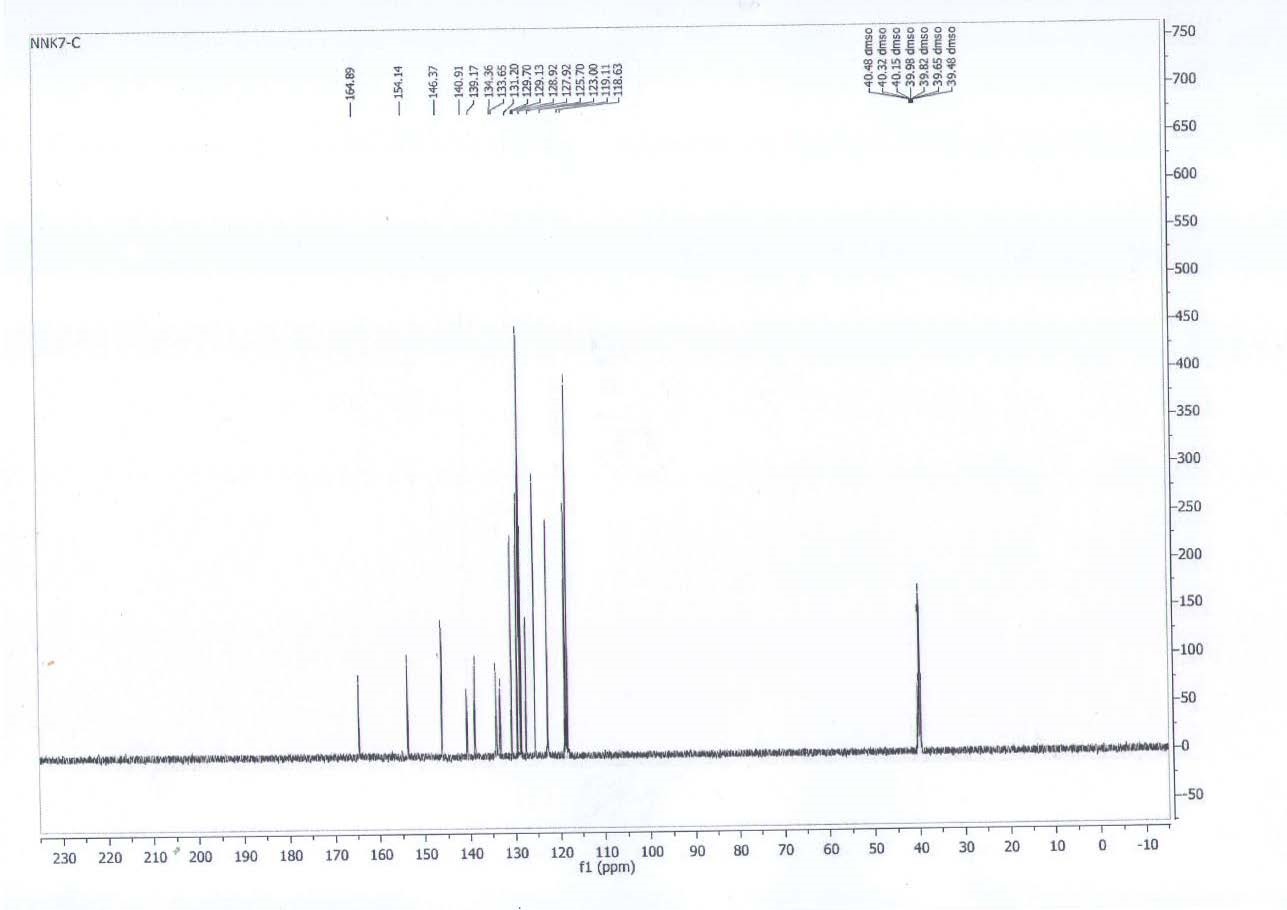


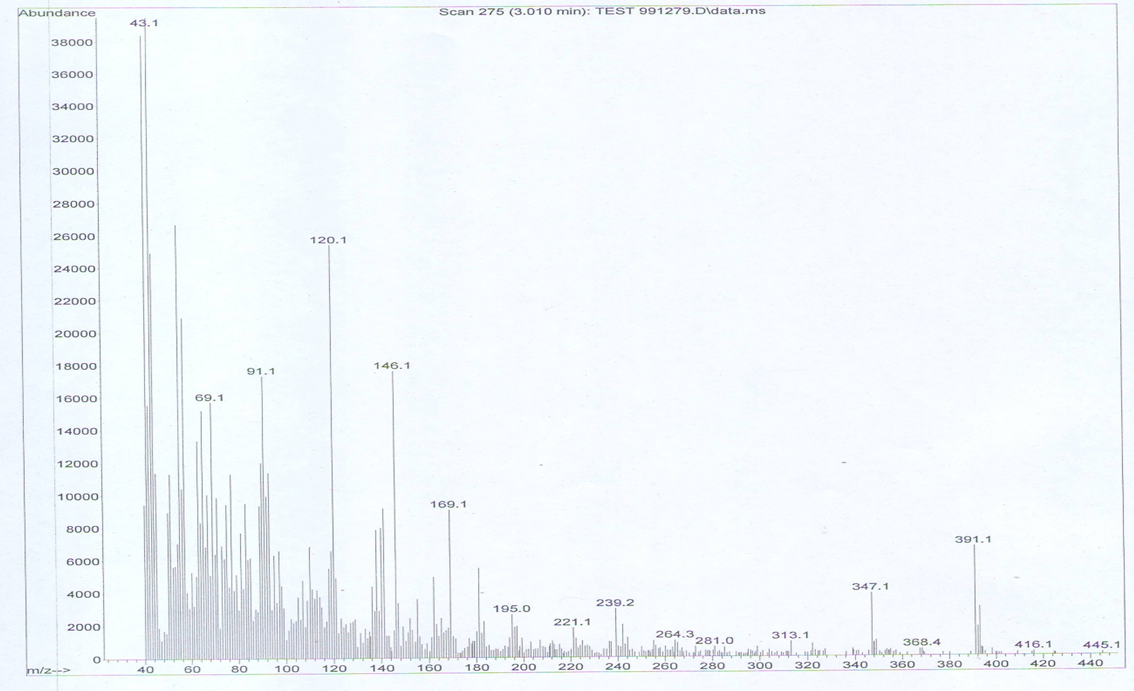


**(4b)**

1-(4-(2-(3-chlorobenzylidene)hydrazine-1-carbonyl)phenyl)-3-phenylthiourea

*
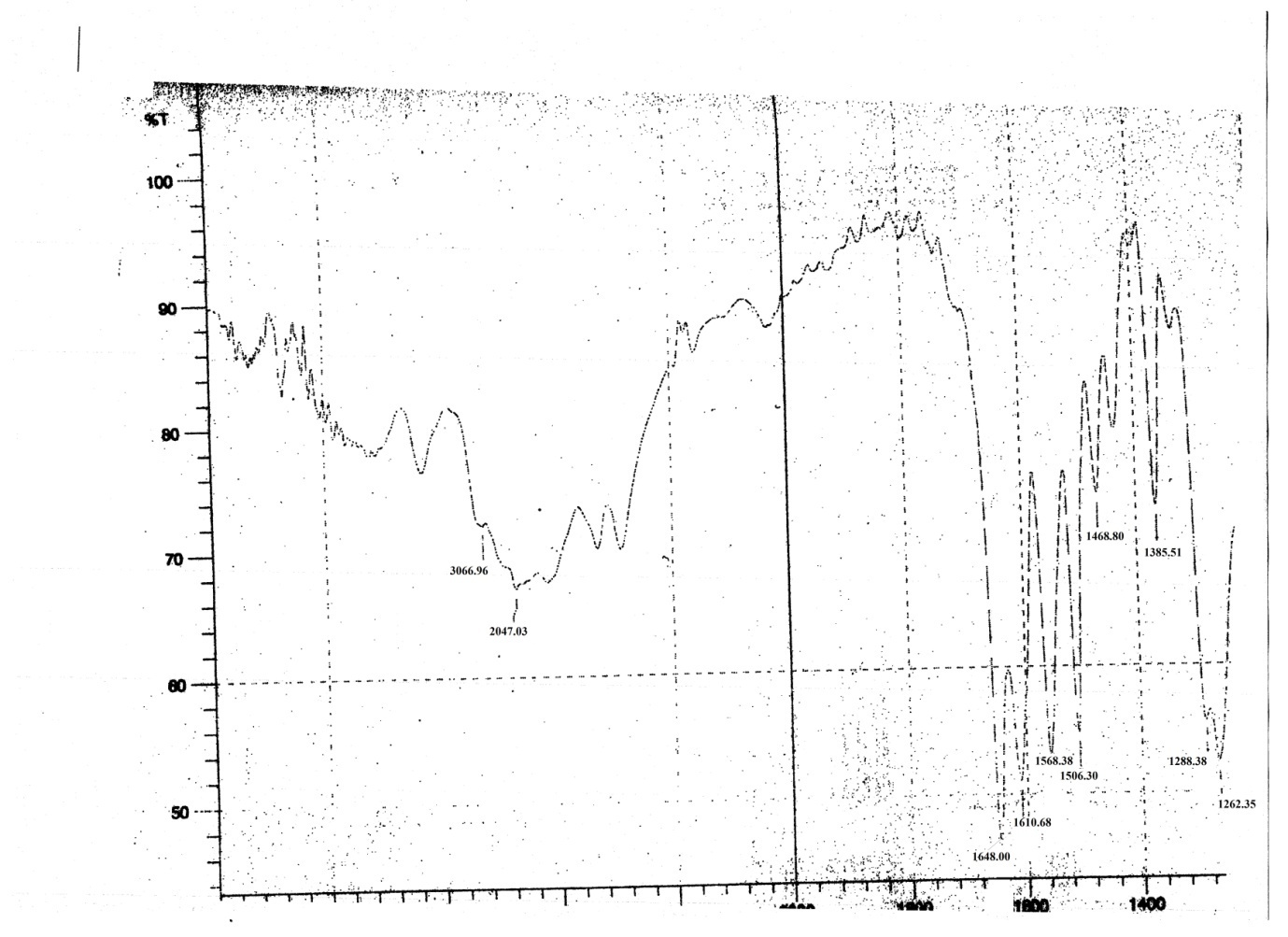
*

*
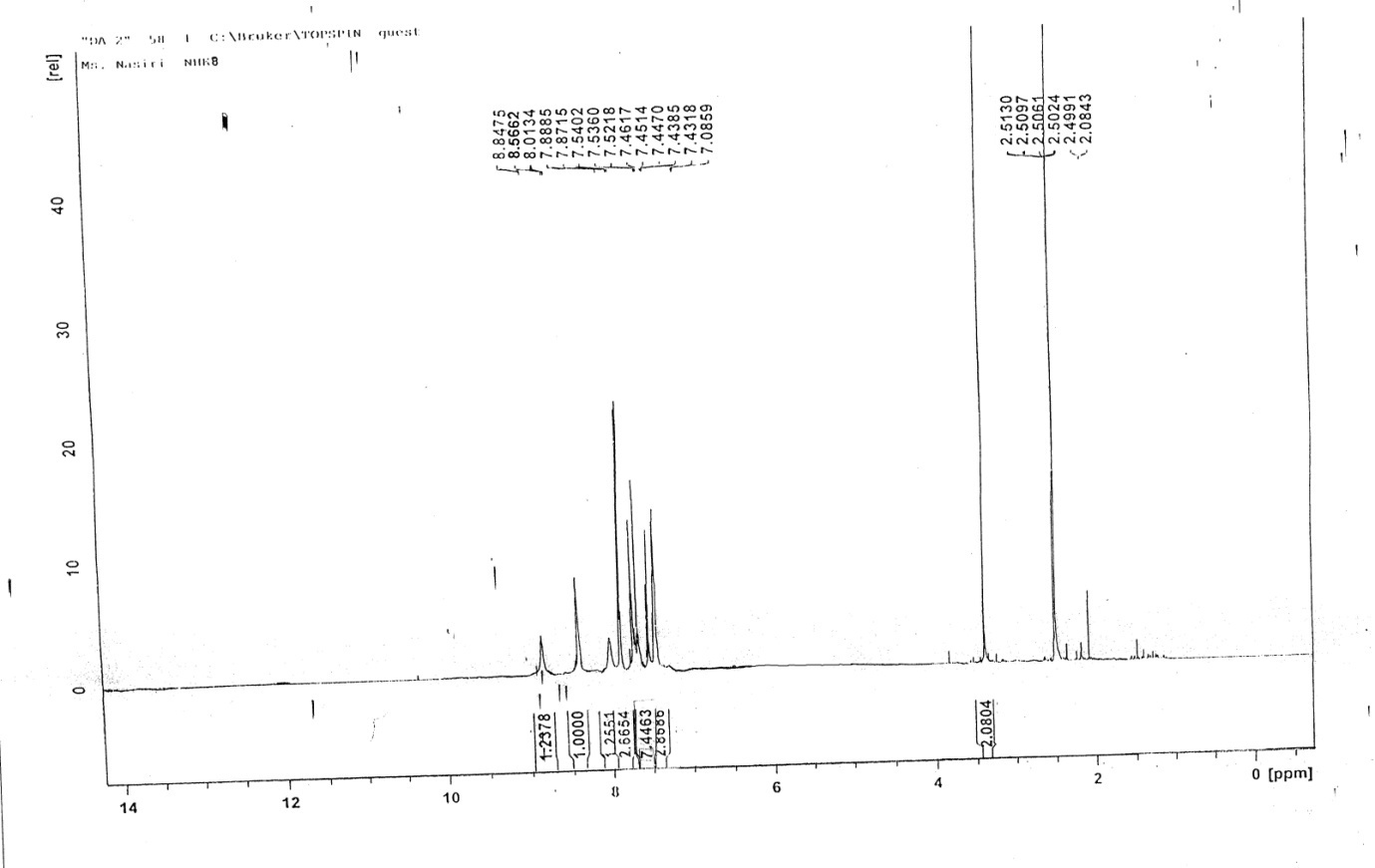
*

*
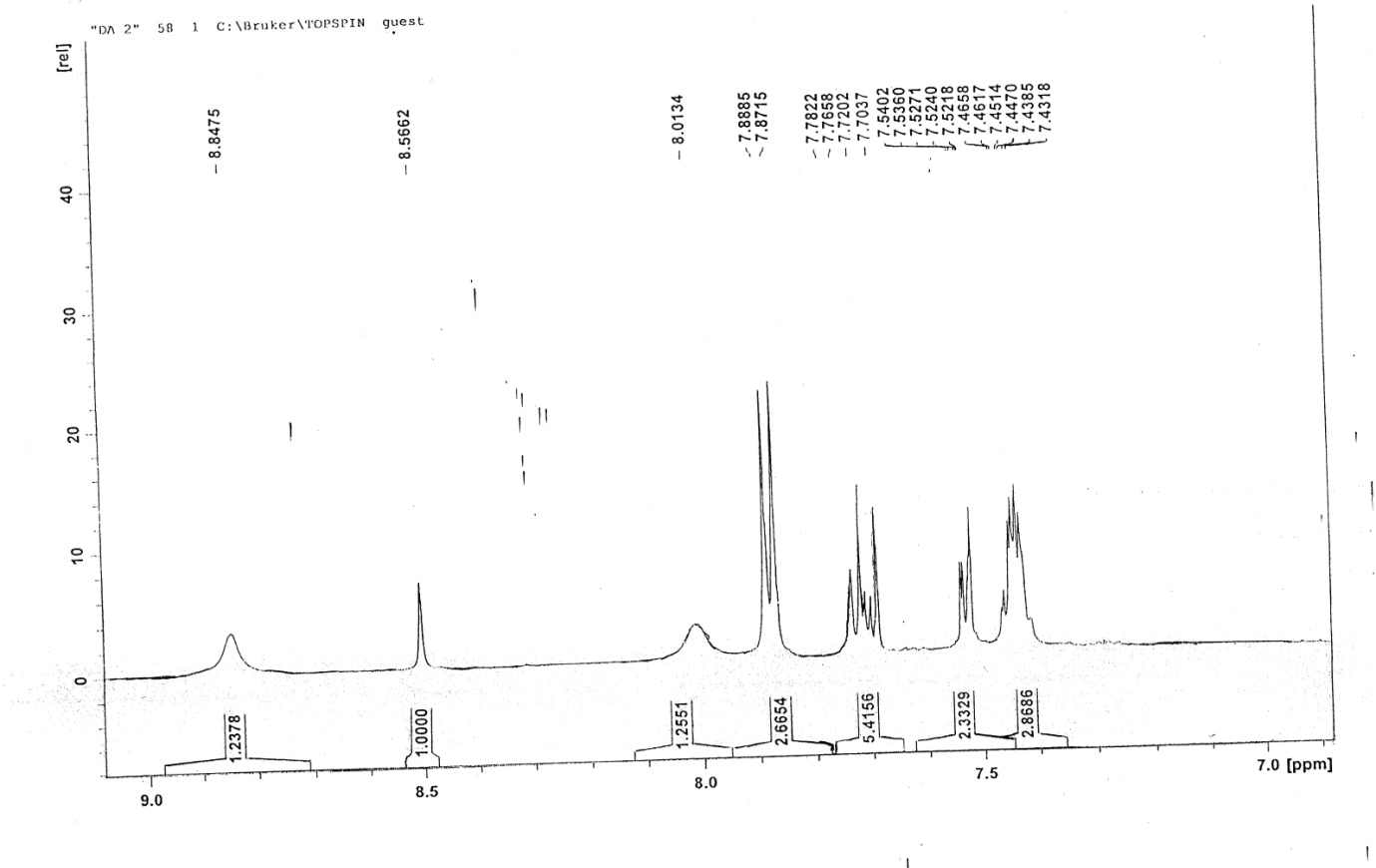
*


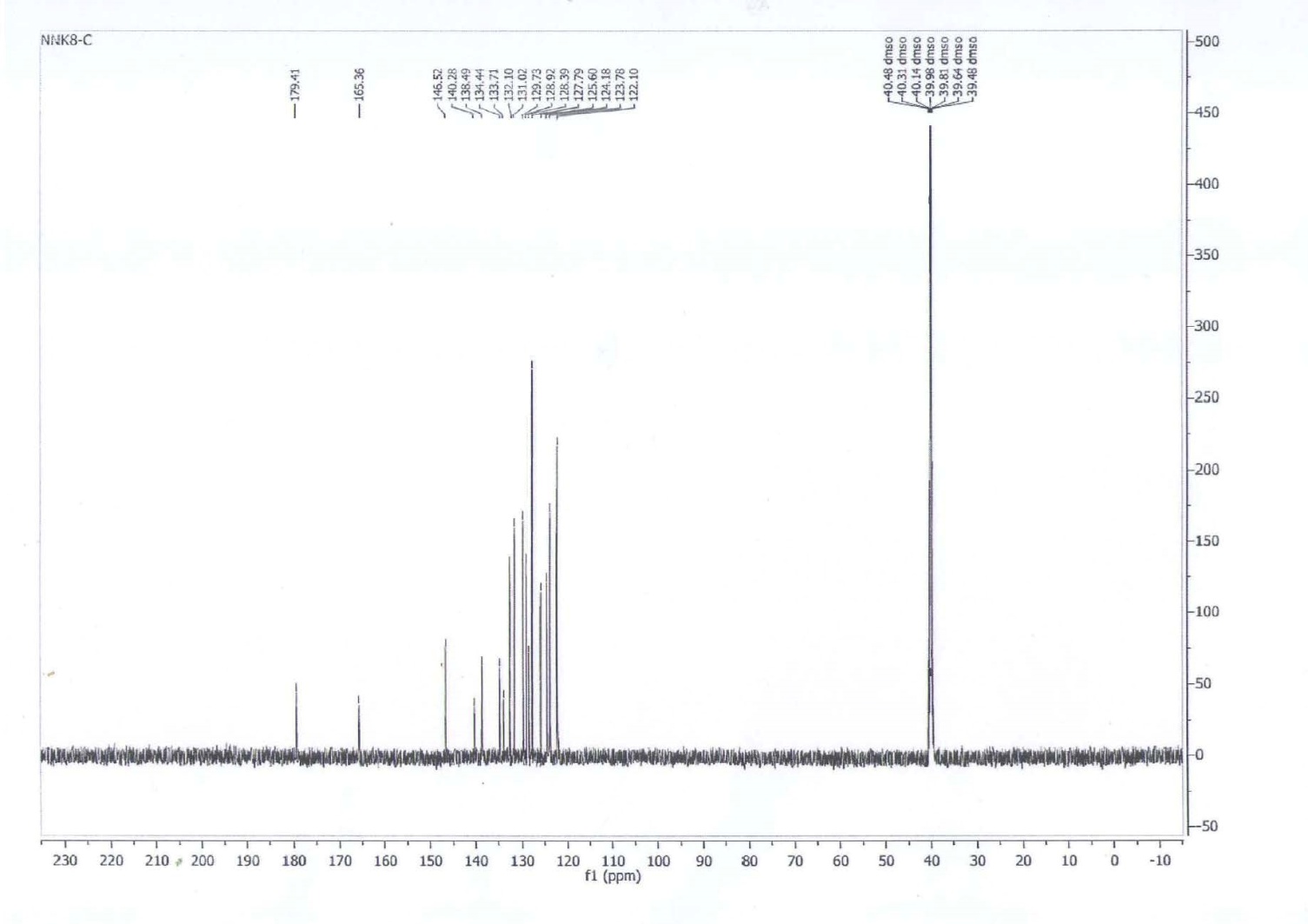


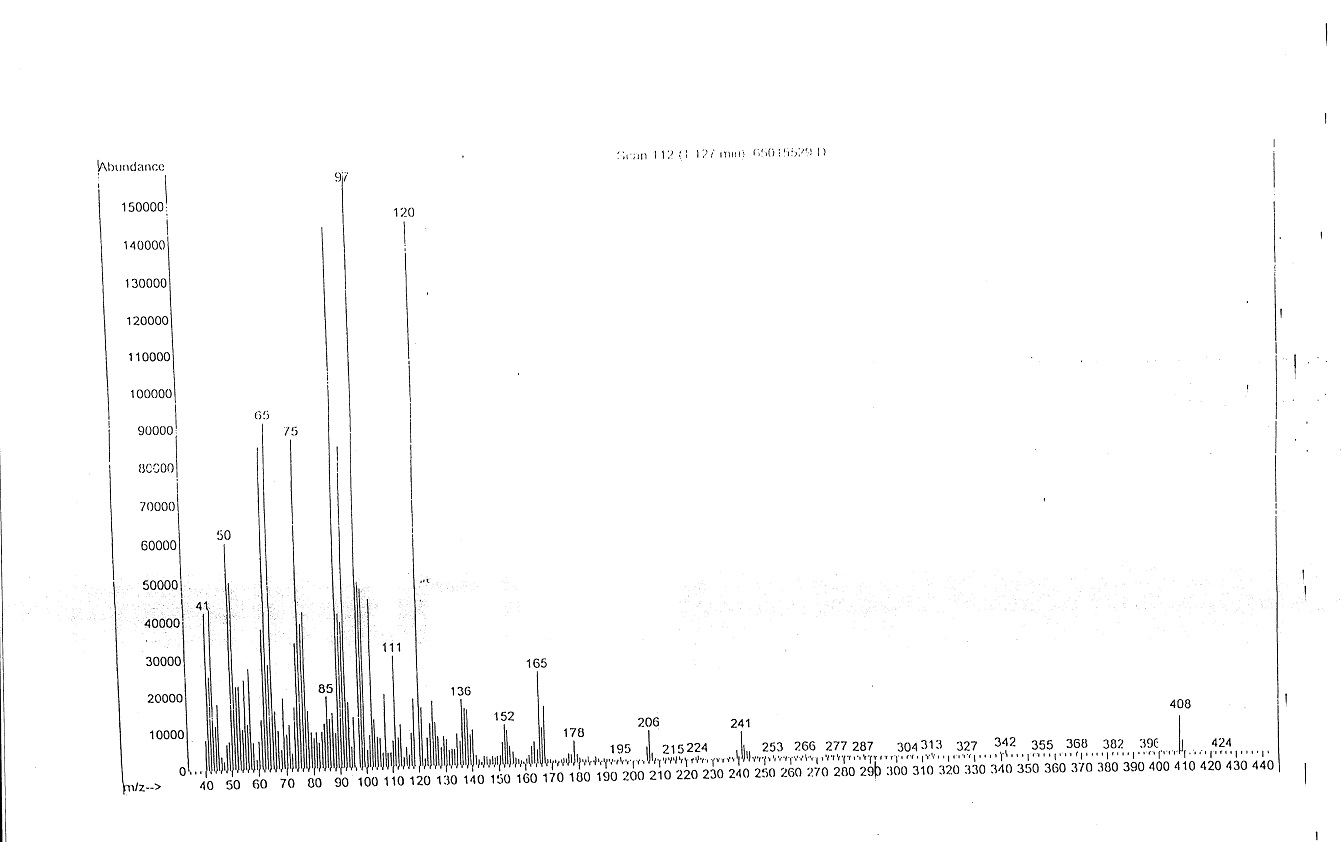


**(4c)**

1-(4-(2-(3-chlorobenzylidene)hydrazine-1-carbonyl)phenyl)-3-(4-chlorophenyl)urea


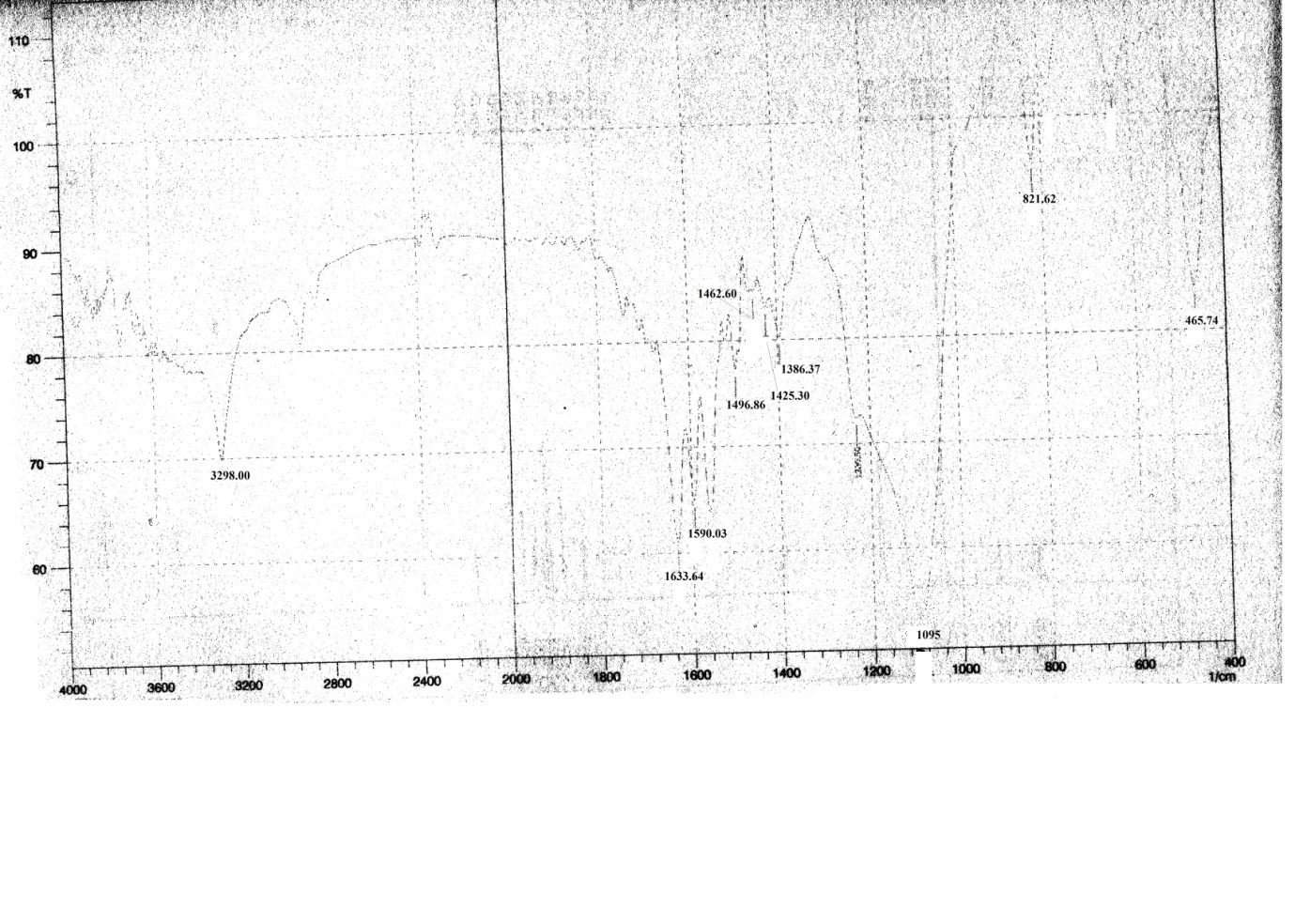


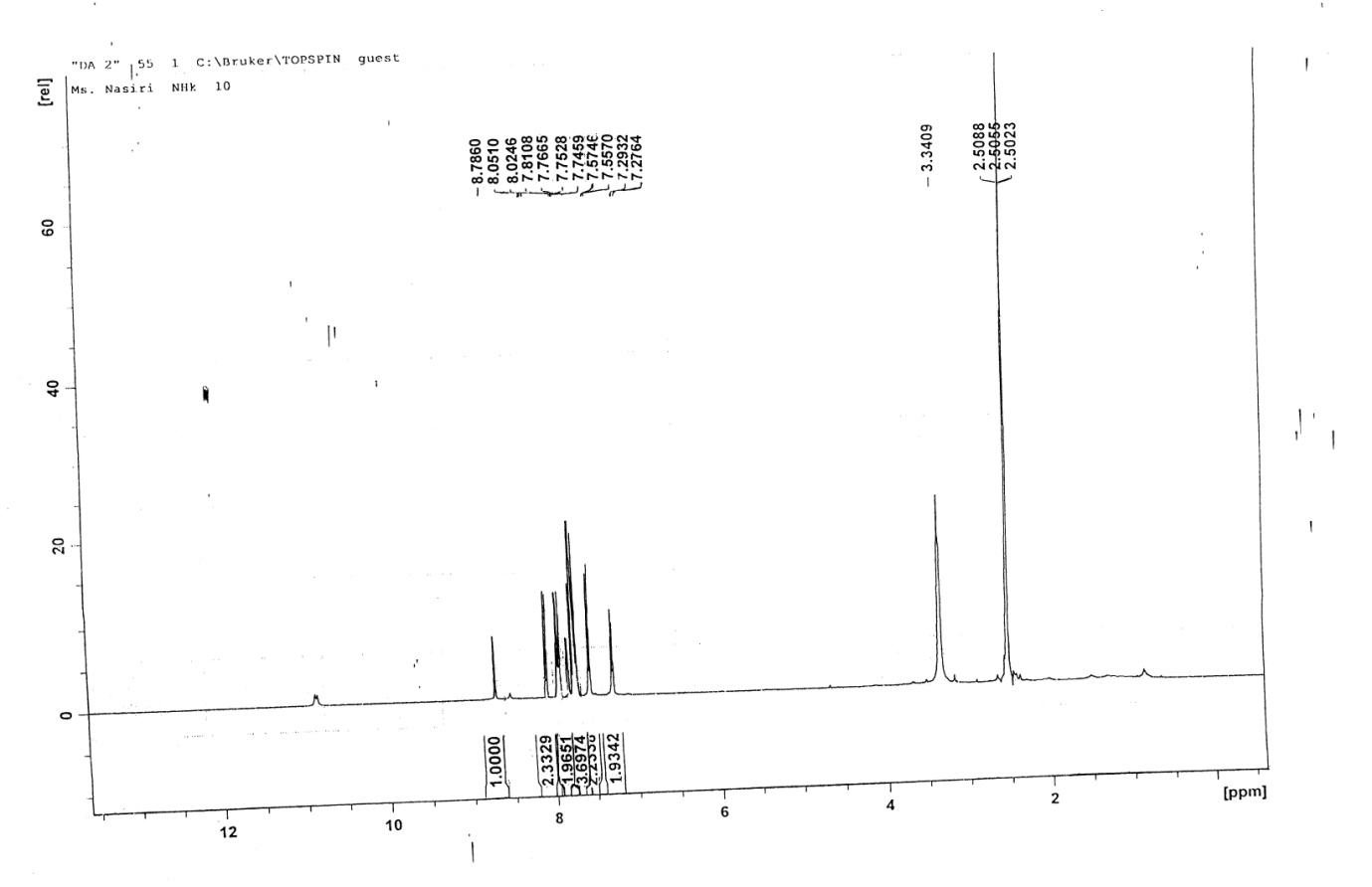


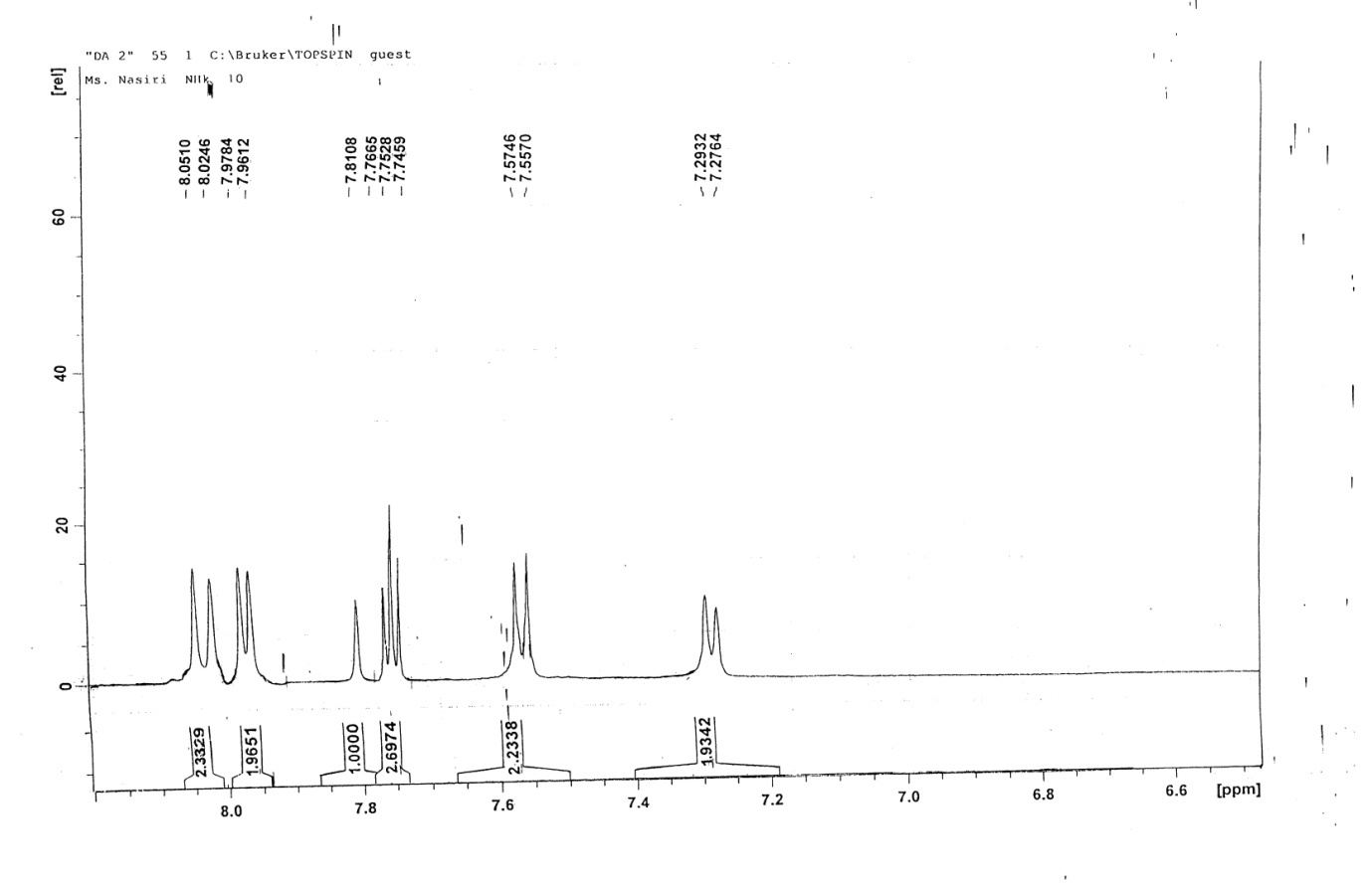


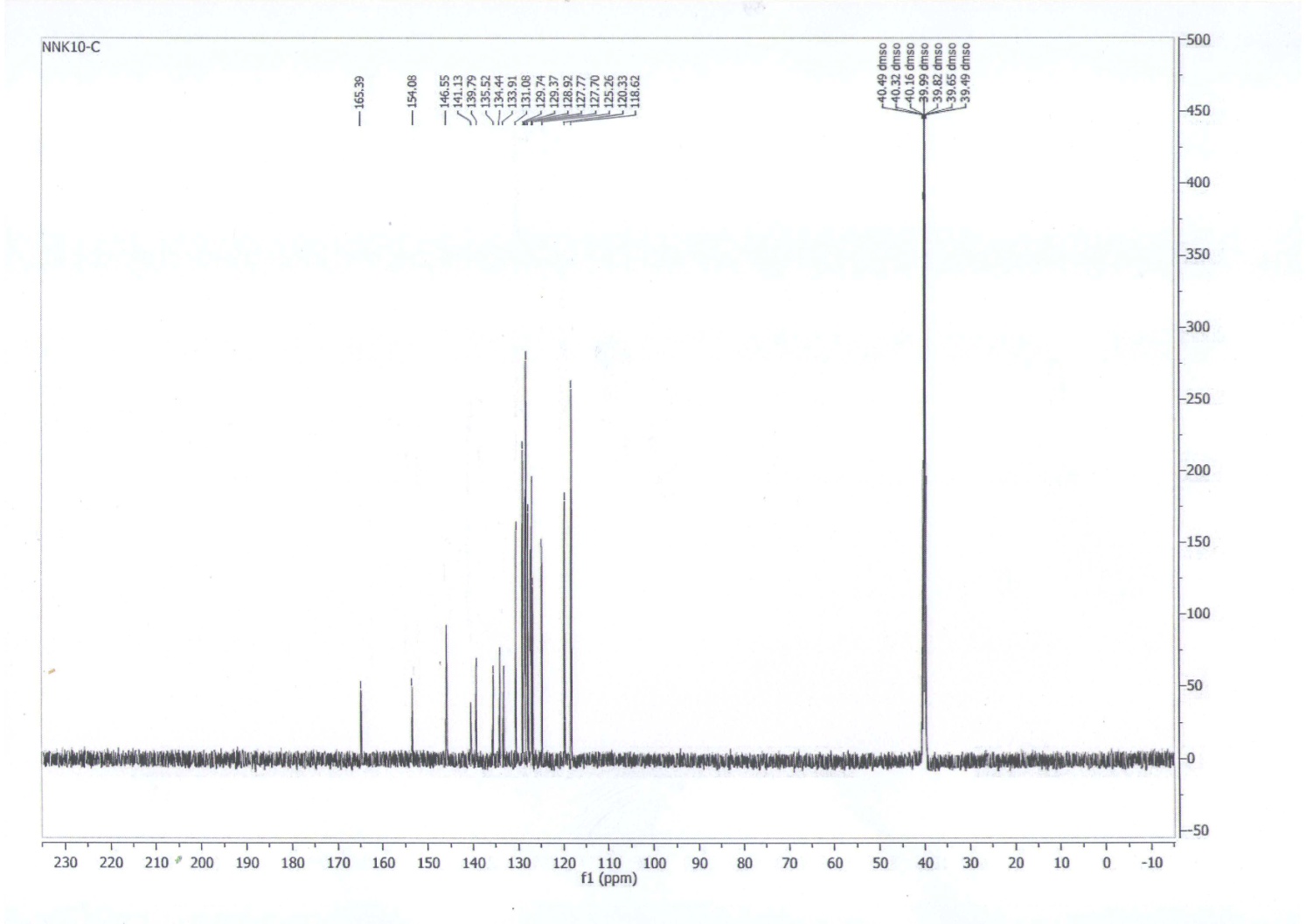


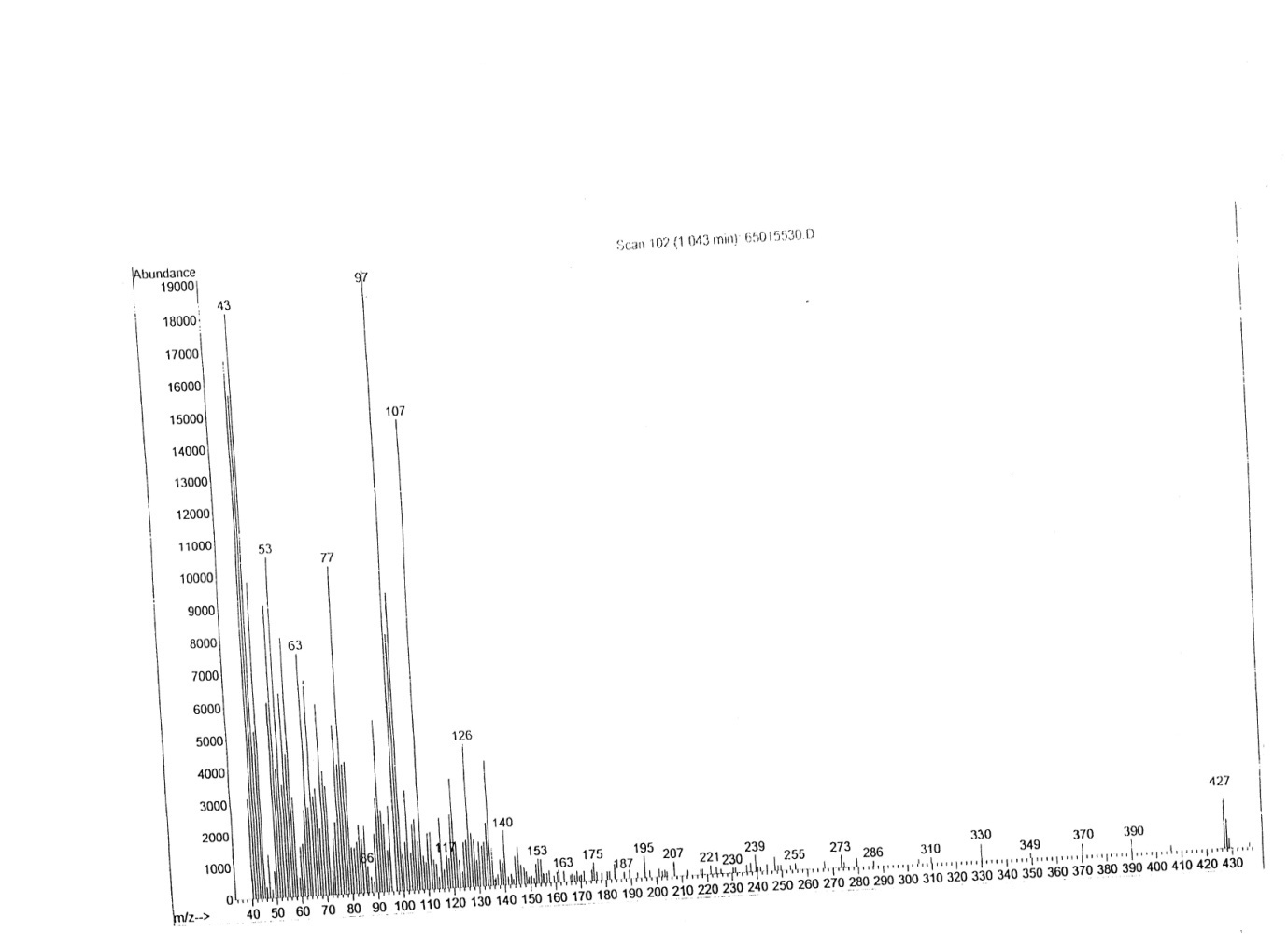


**(4d)**

N-((4-(2-(3-chlorobenzylidene)hydrazine-1-carbonyl)phenyl)carbamoyl)-4-methylbenzenesulfonamide

*
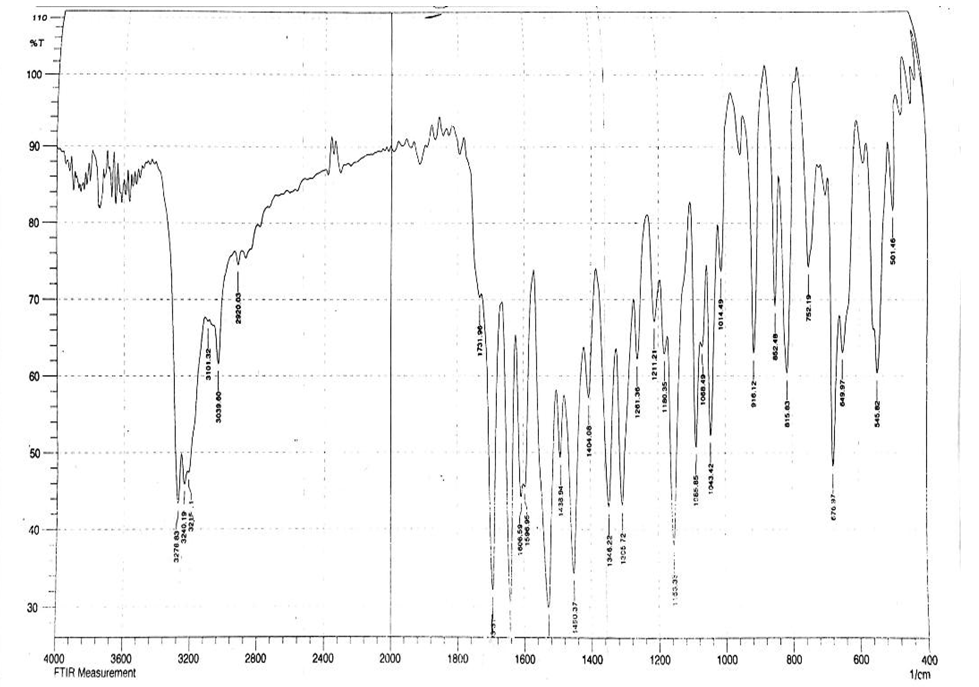
*


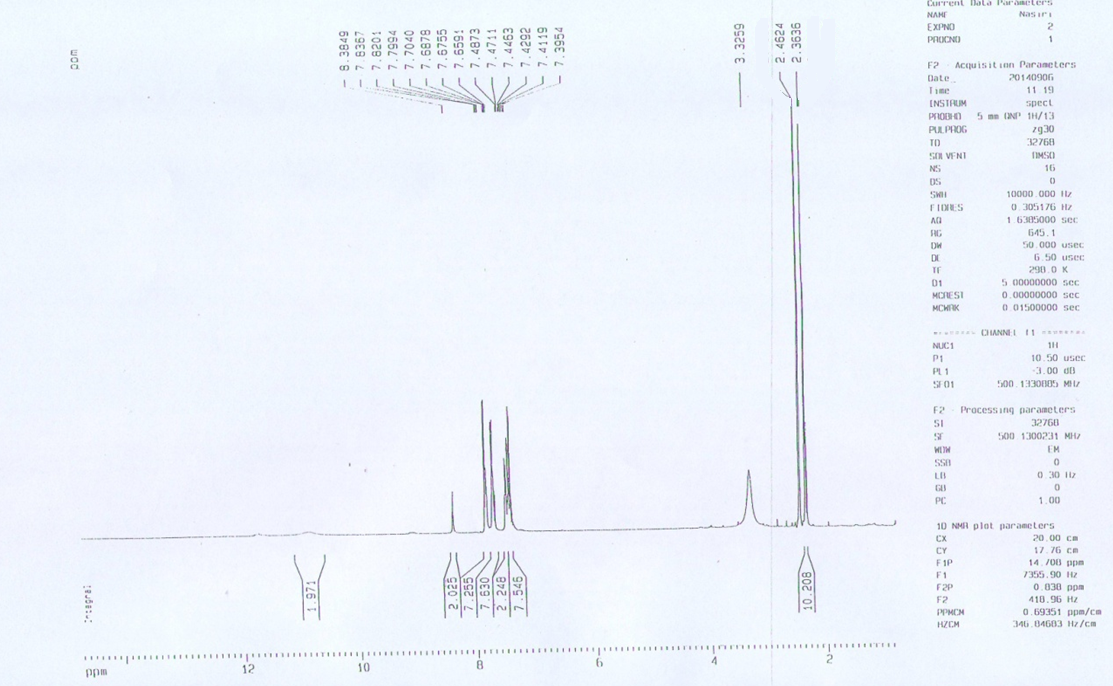


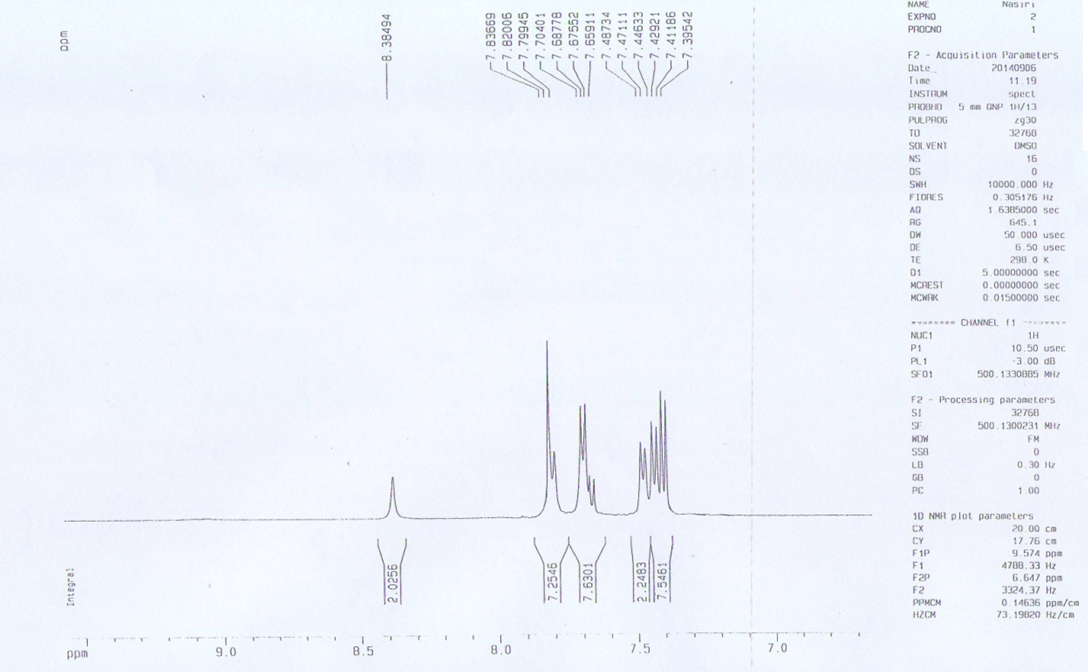


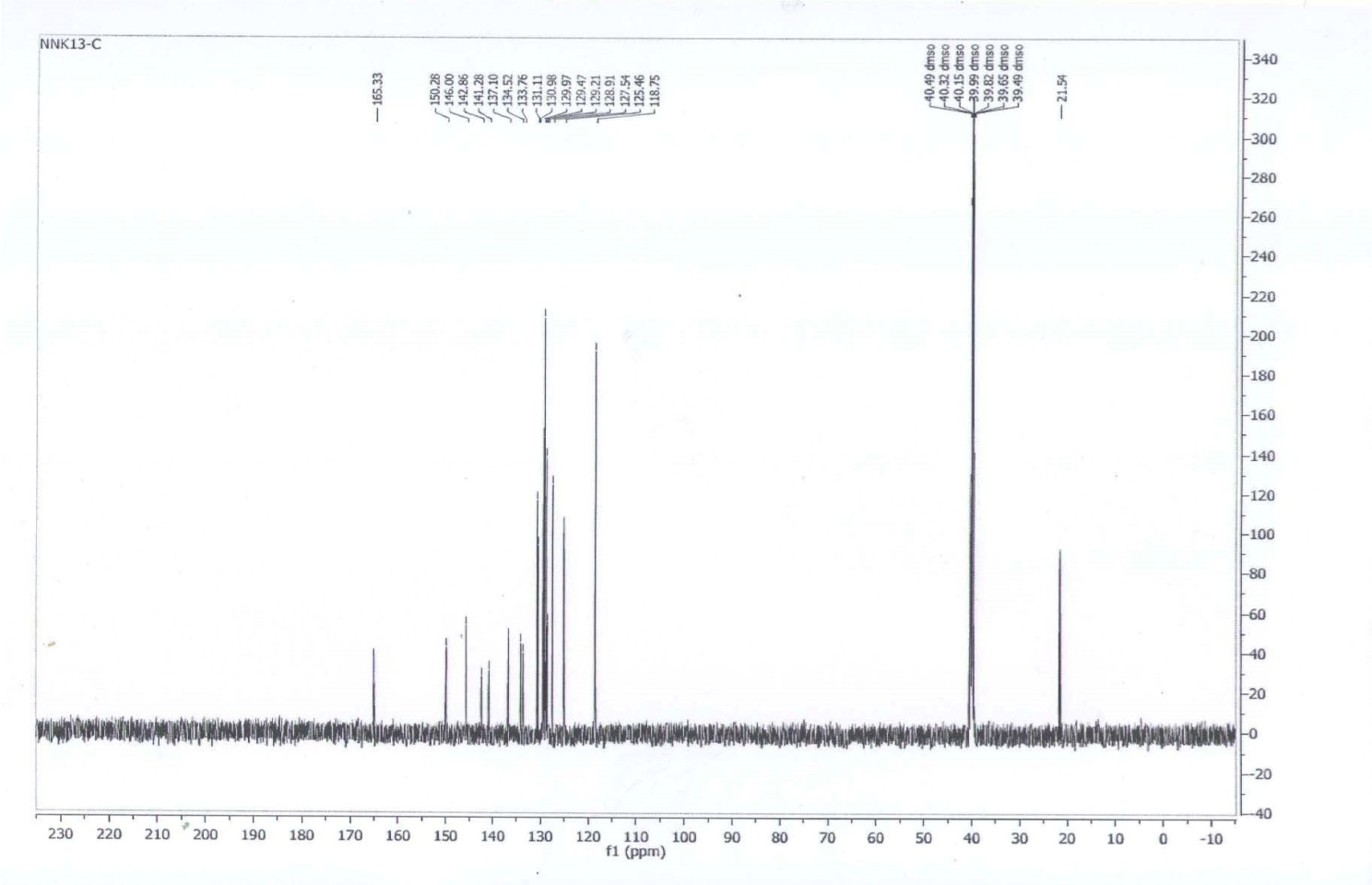


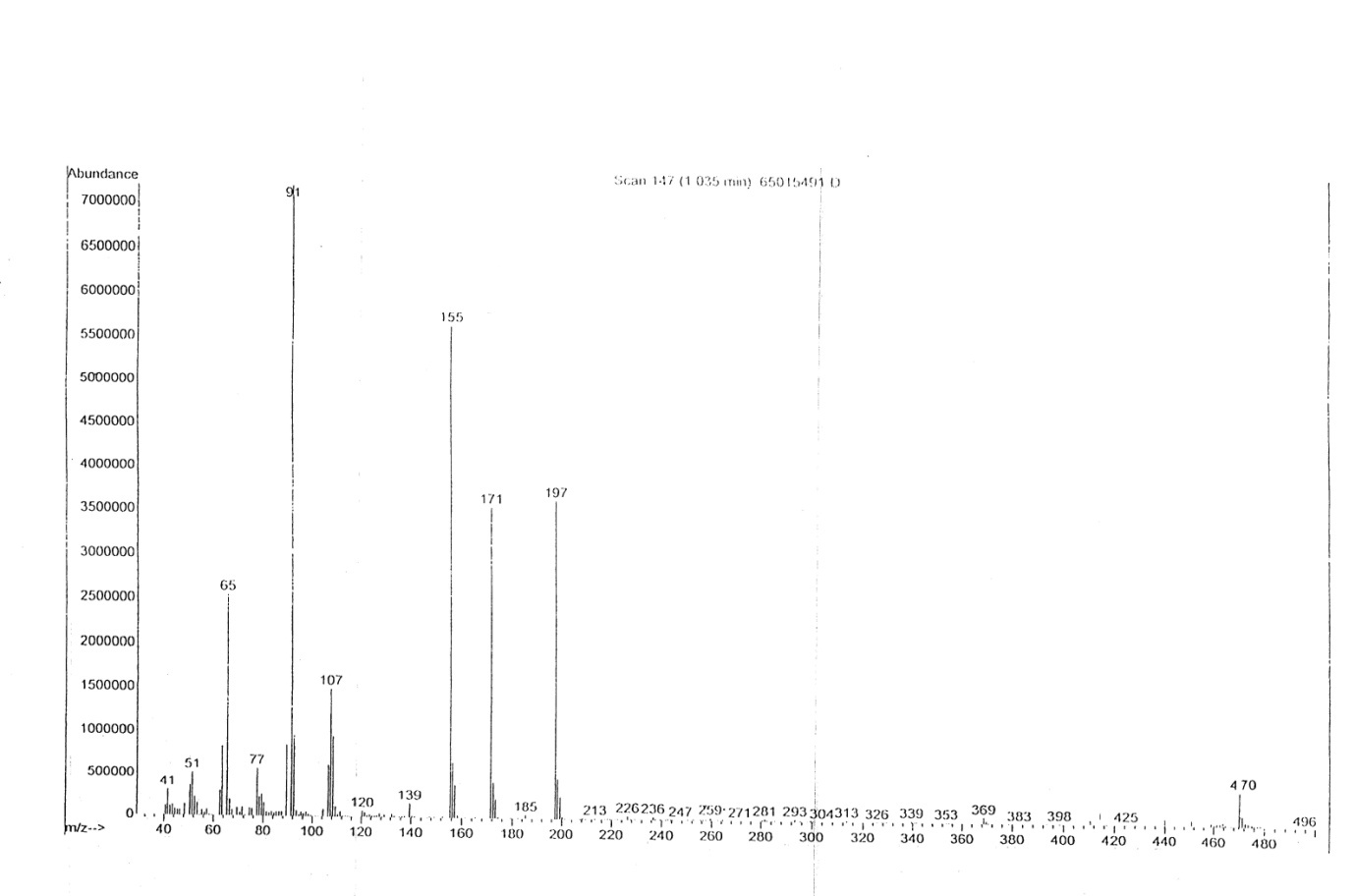


**(4e)**

1-(4-chlorophenyl)-3-(4-(2-(3-nitrobenzylidene)hydrazine-1-carbonyl)phenyl)urea

*
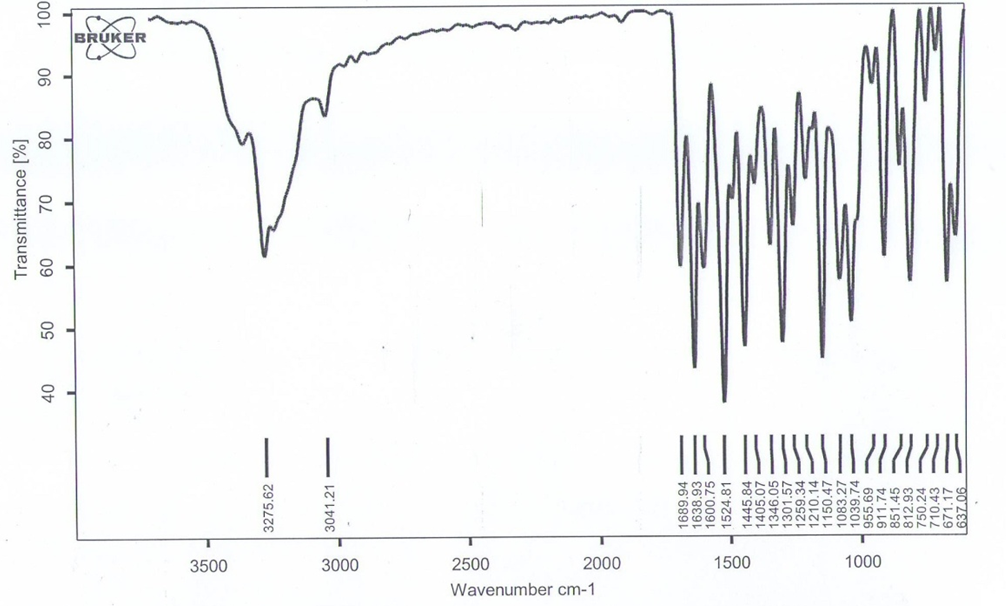
*

*
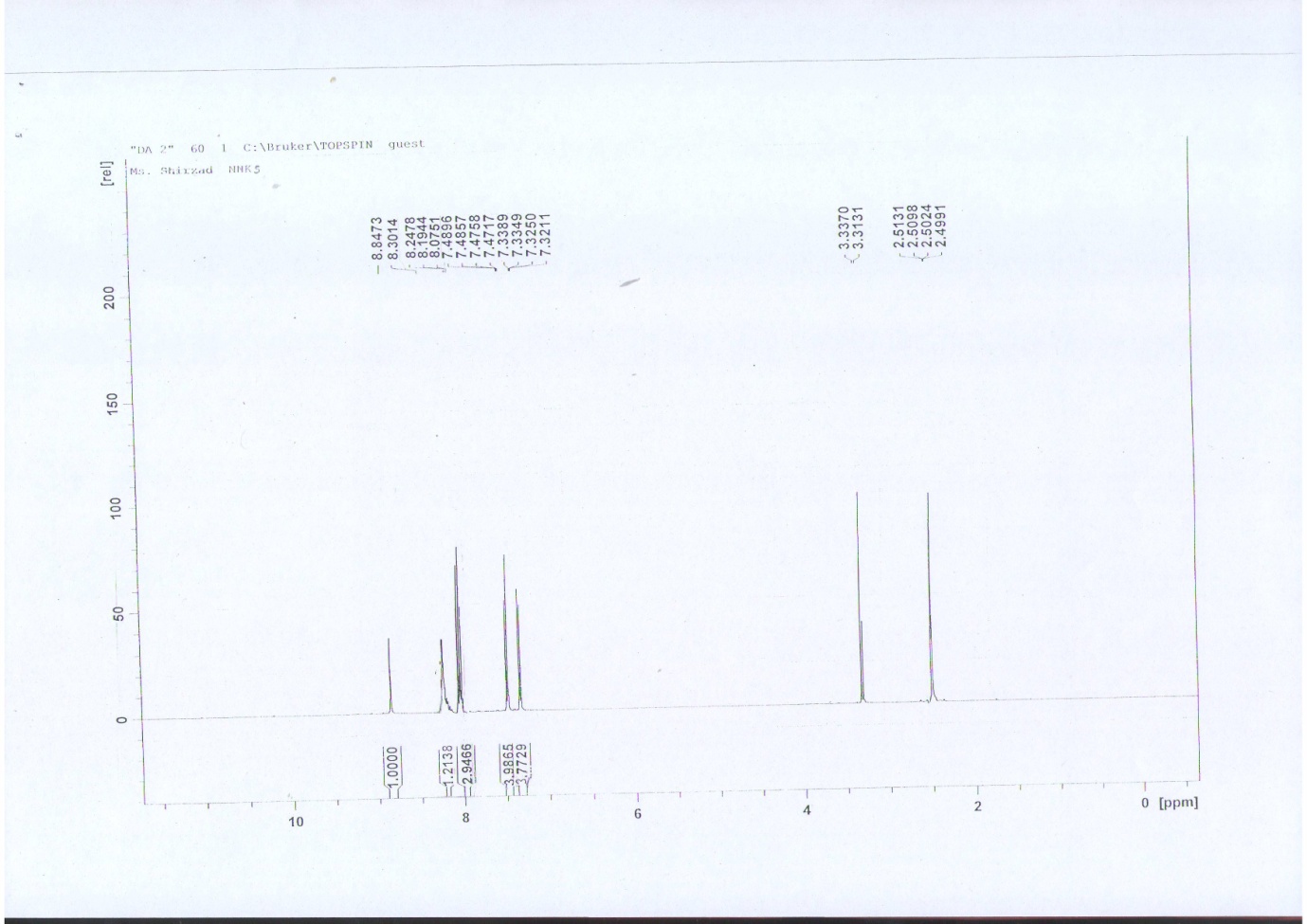
*

*
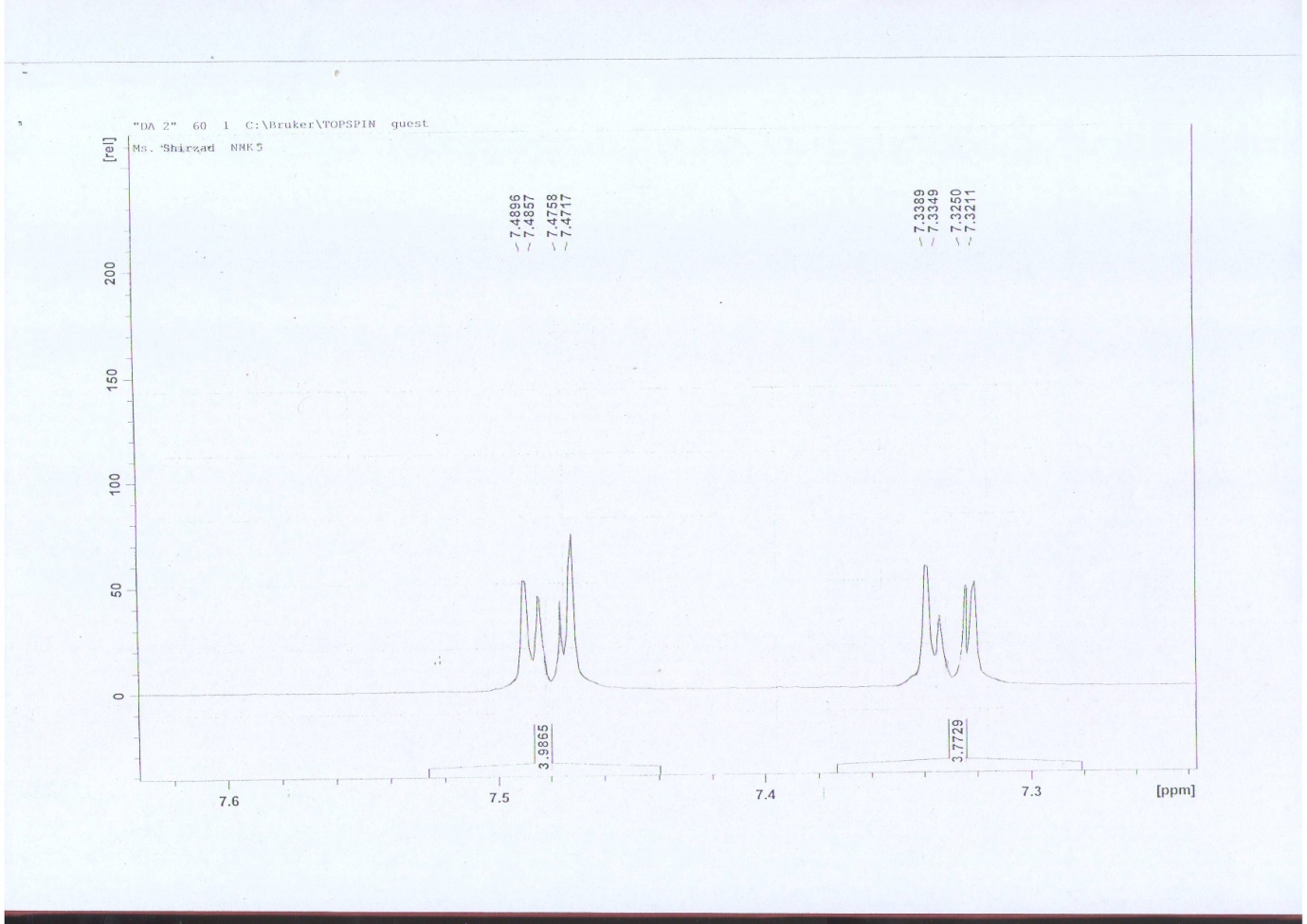
*

*
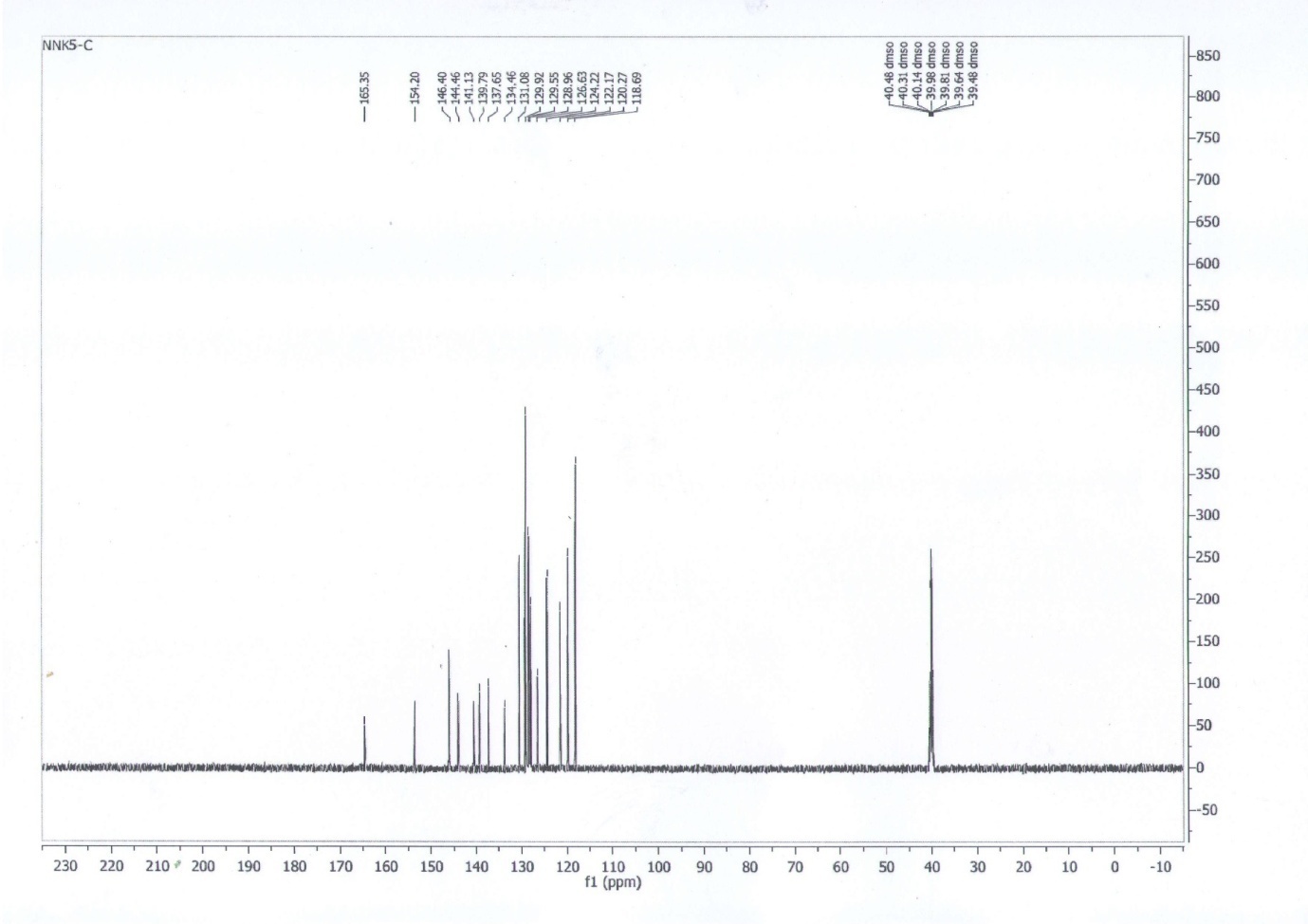
*


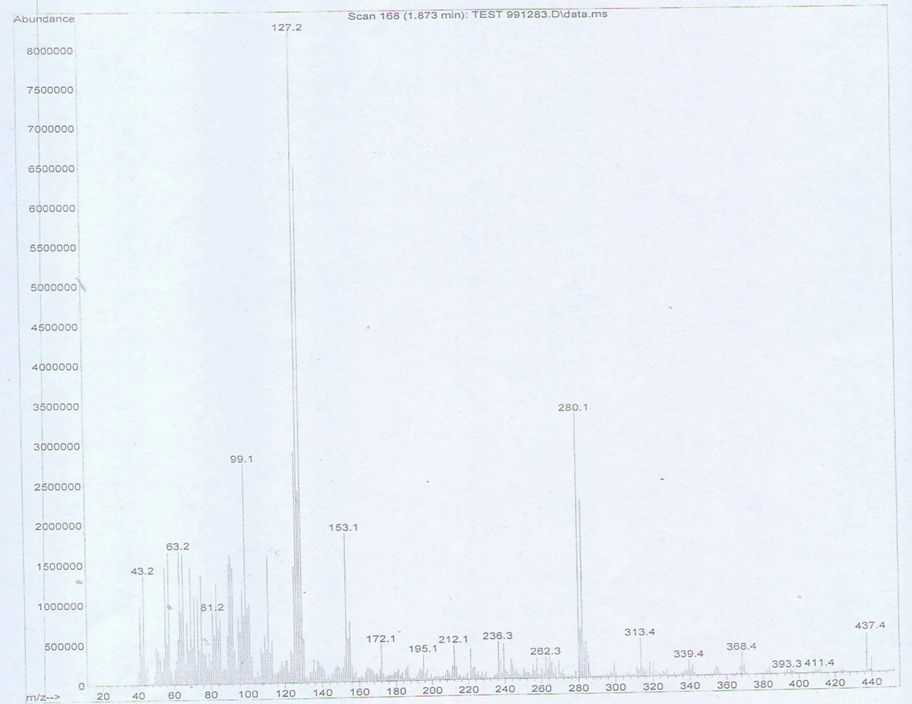


**(4f)**

1-(4-(2-(3-nitrobenzylidene)hydrazine-1-carbonyl)phenyl)-3-phenylurea

*
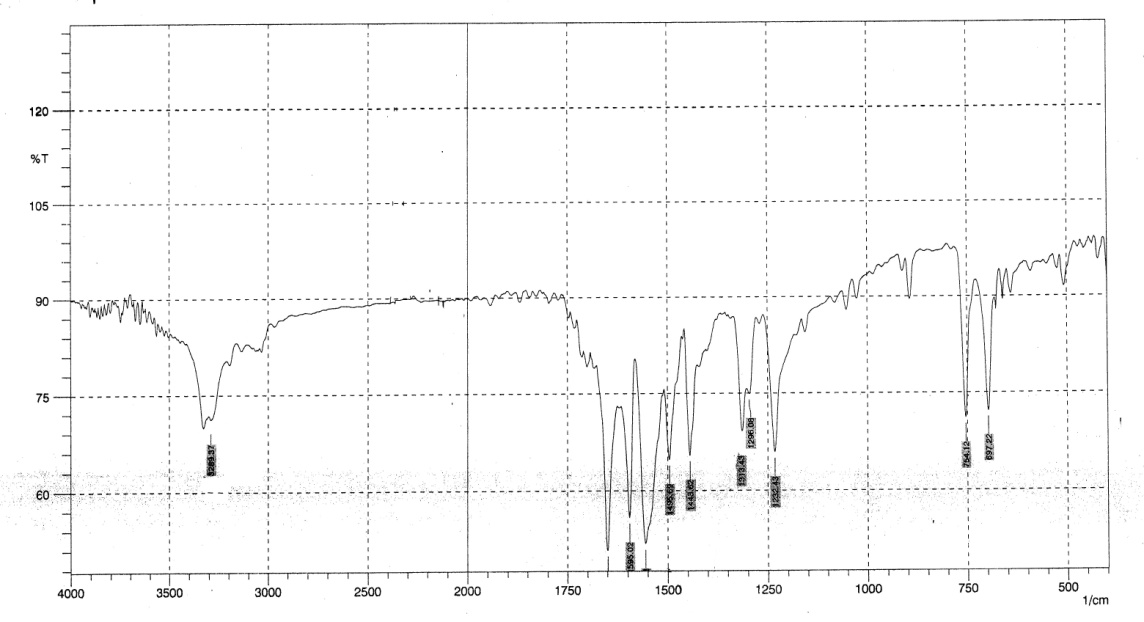
*


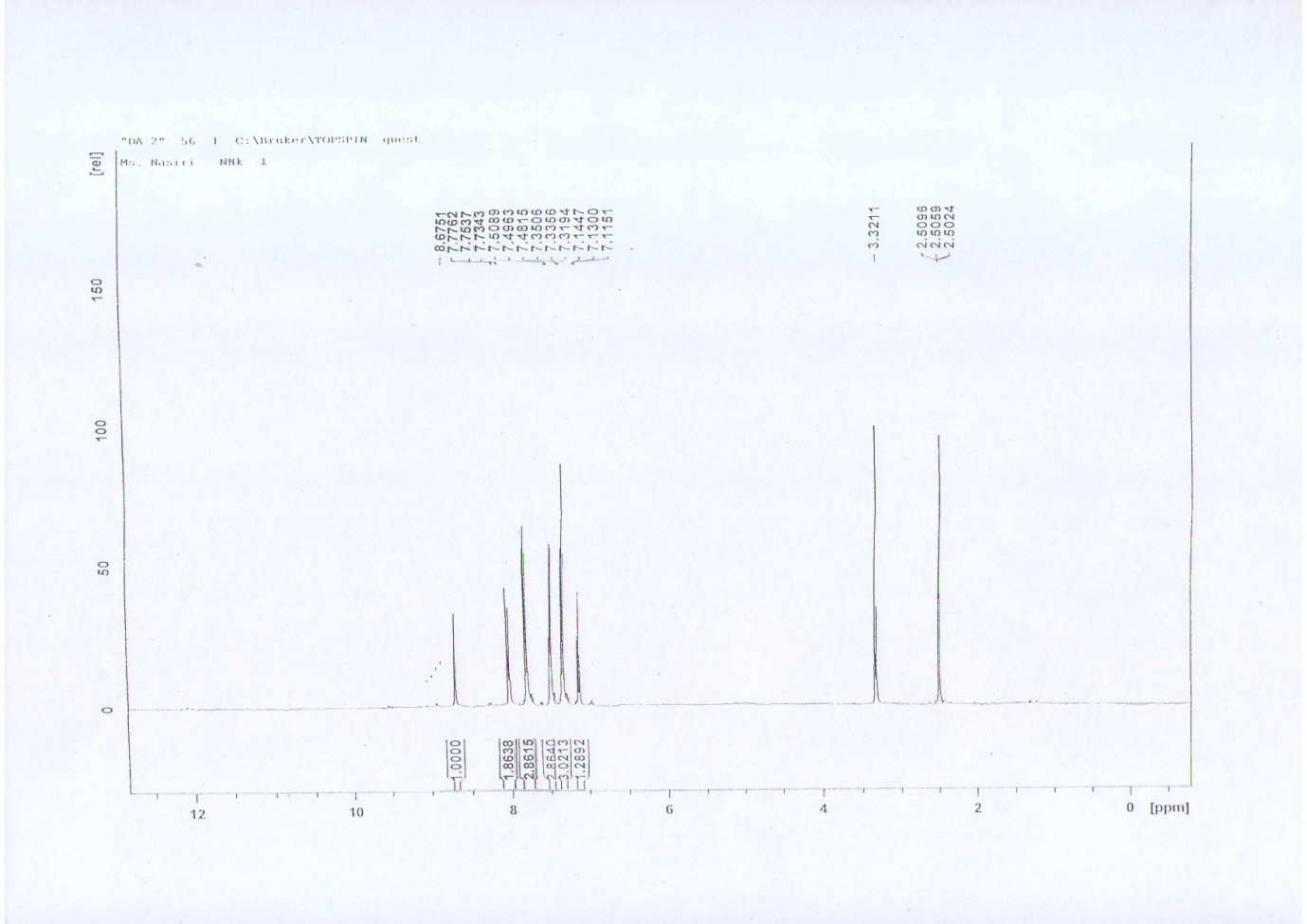


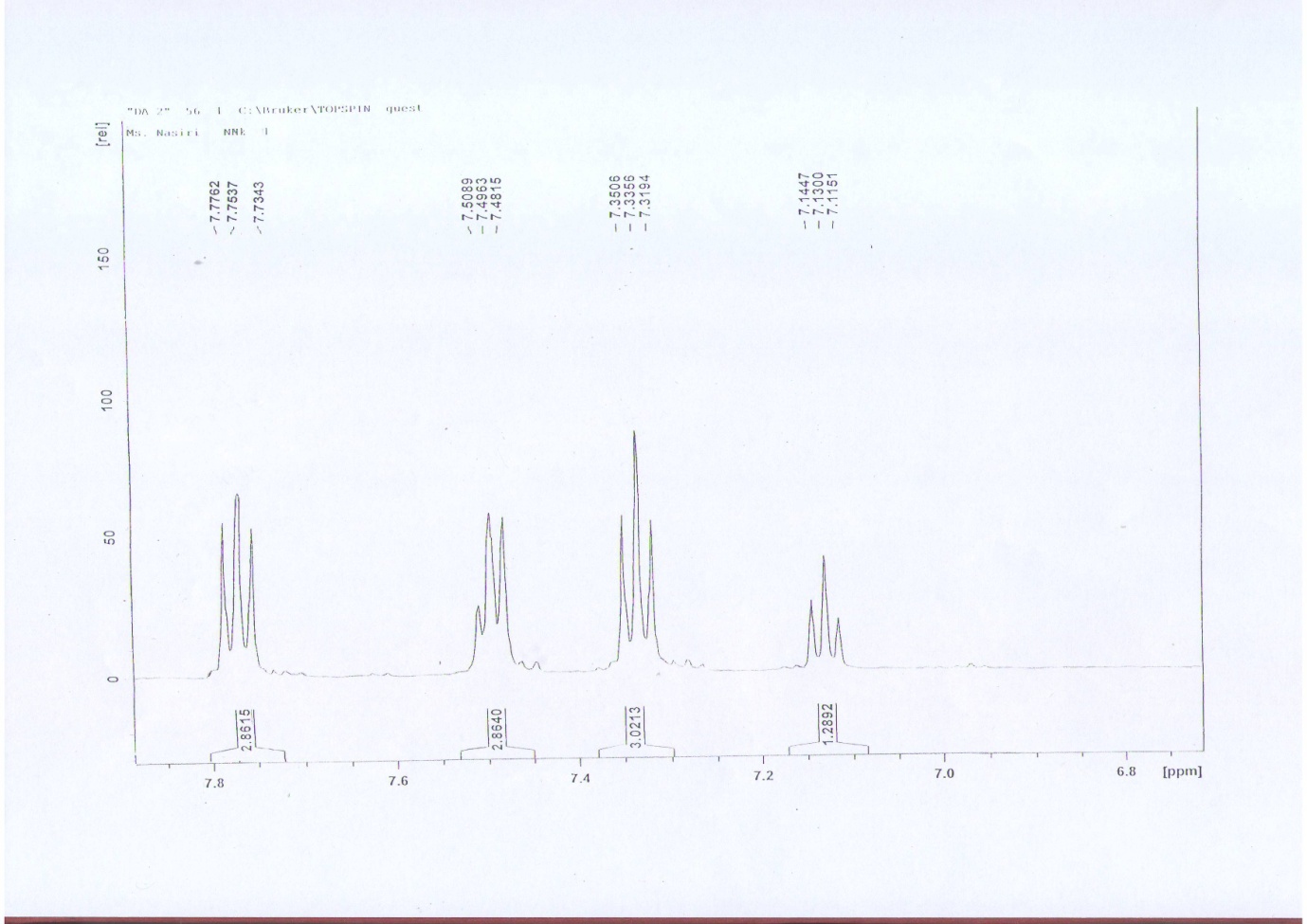


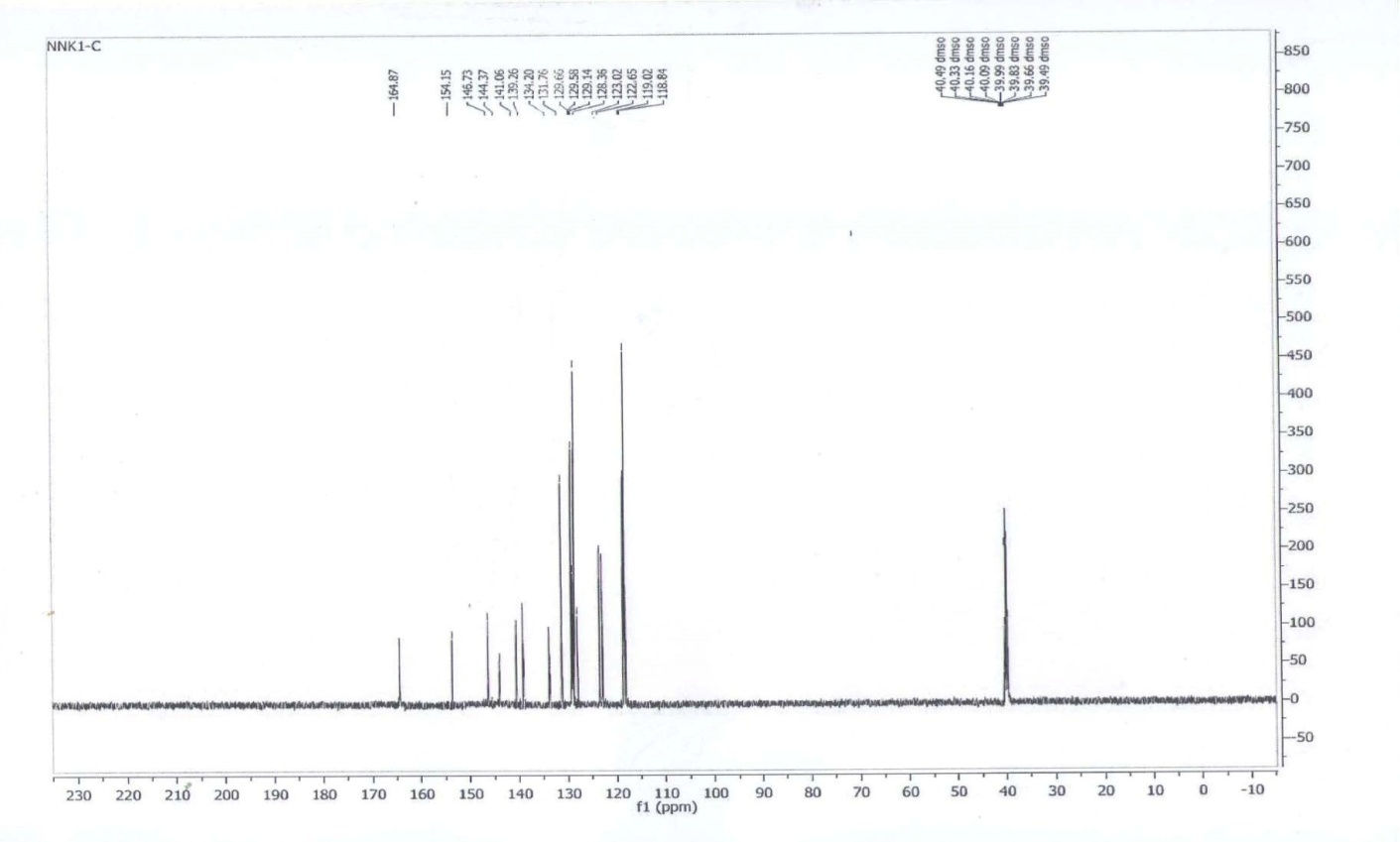


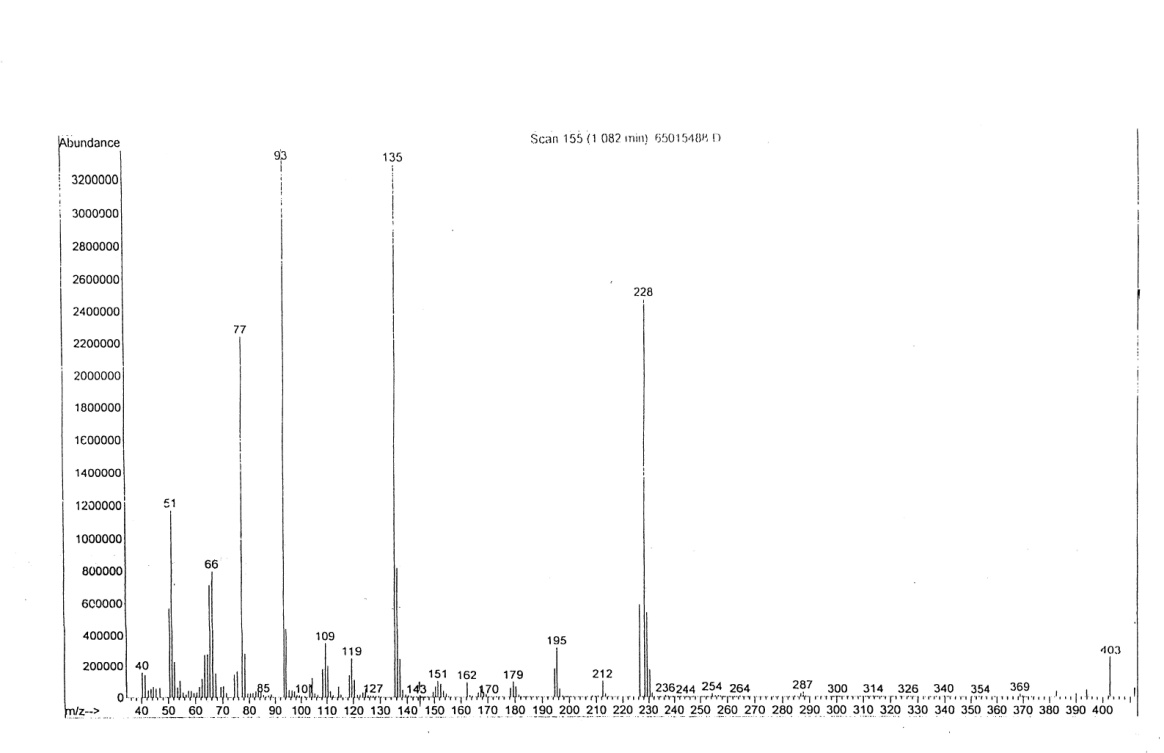


**(4g)**

4-methyl-N-((4-(2-(3-nitrobenzylidene)hydrazine-1-carbonyl)phenyl)carbamoyl) benzenesulfonamide


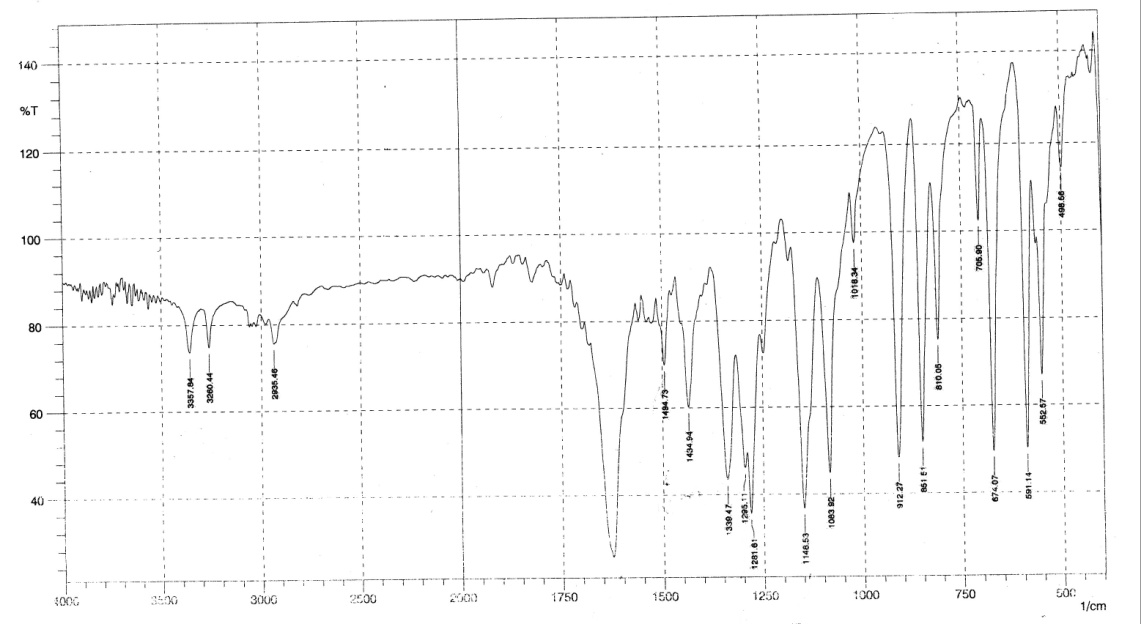


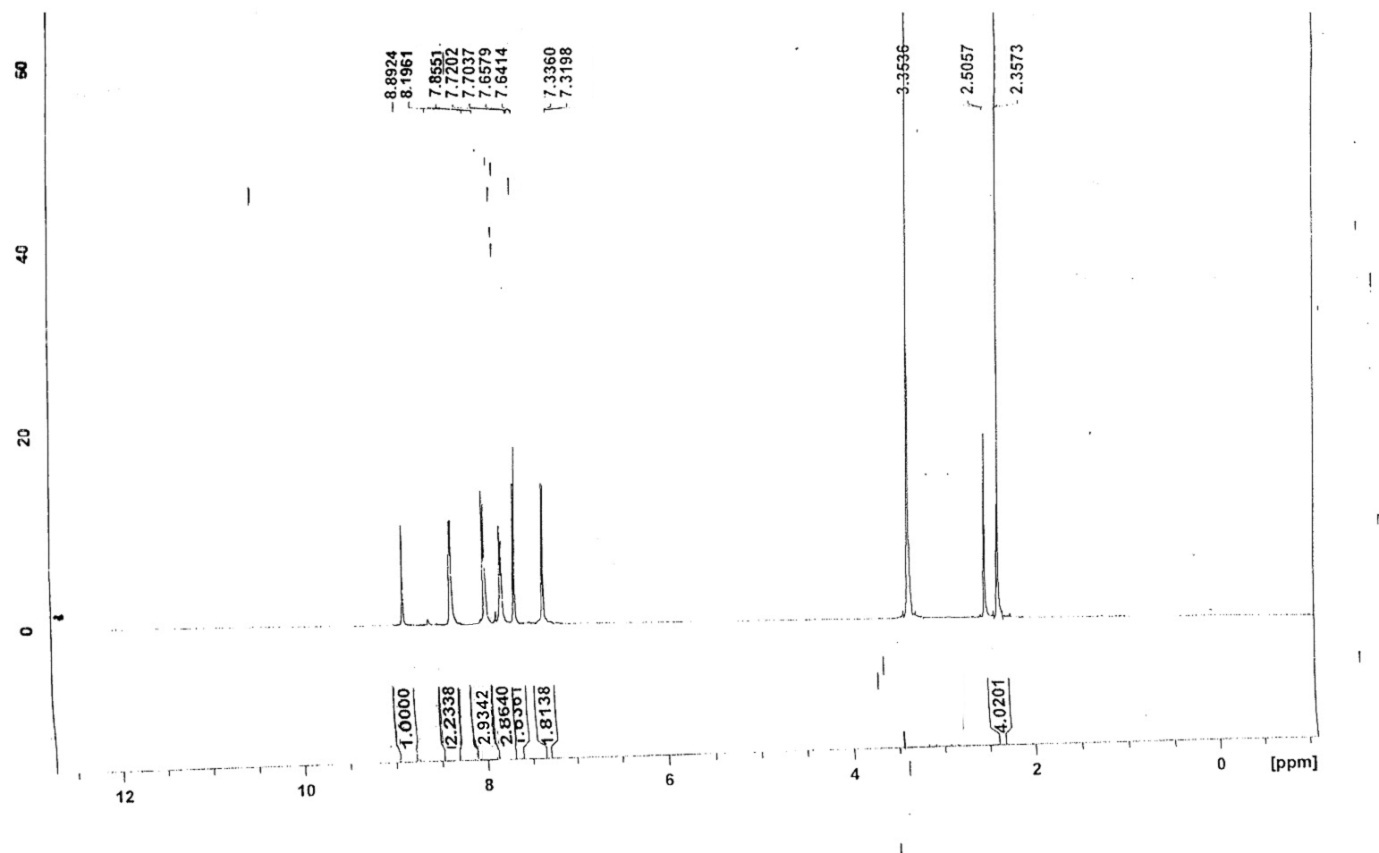


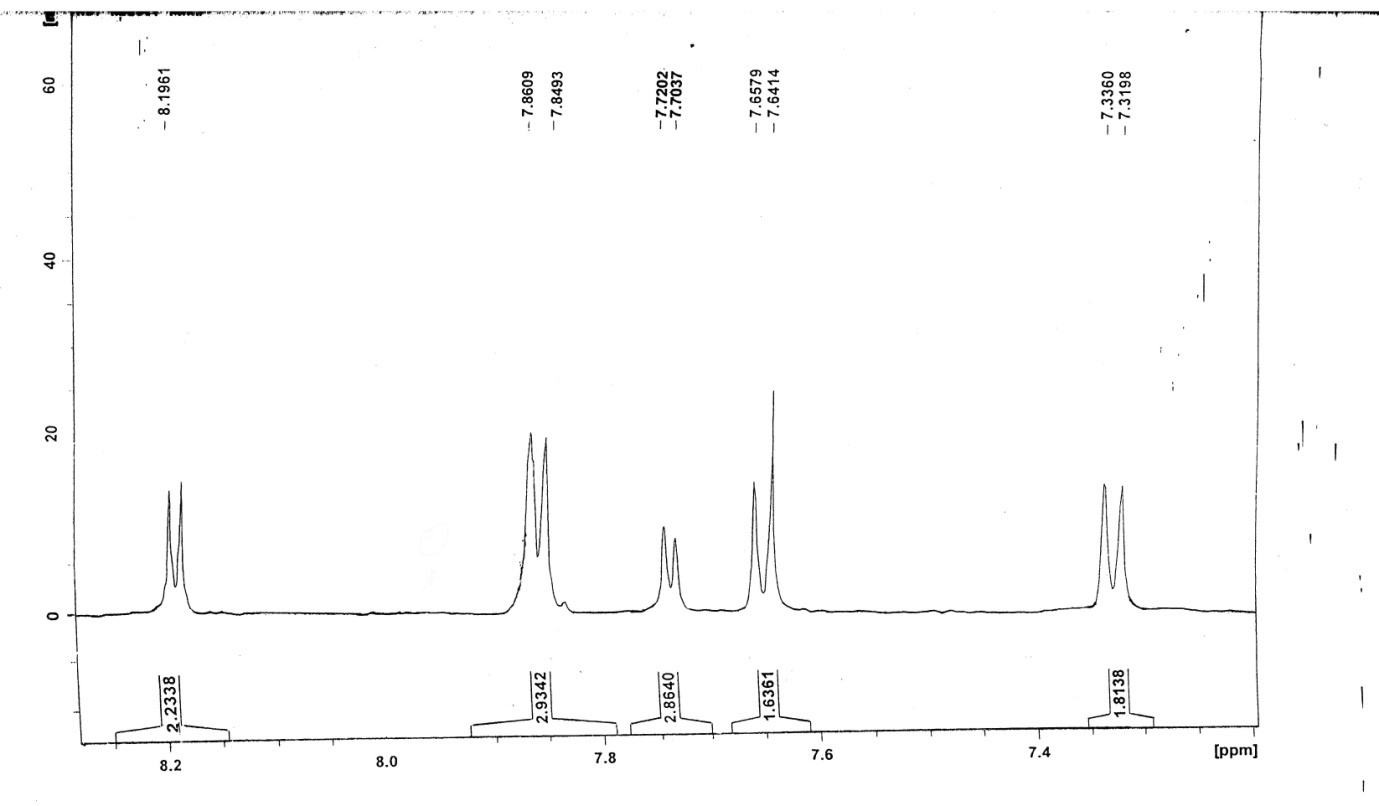


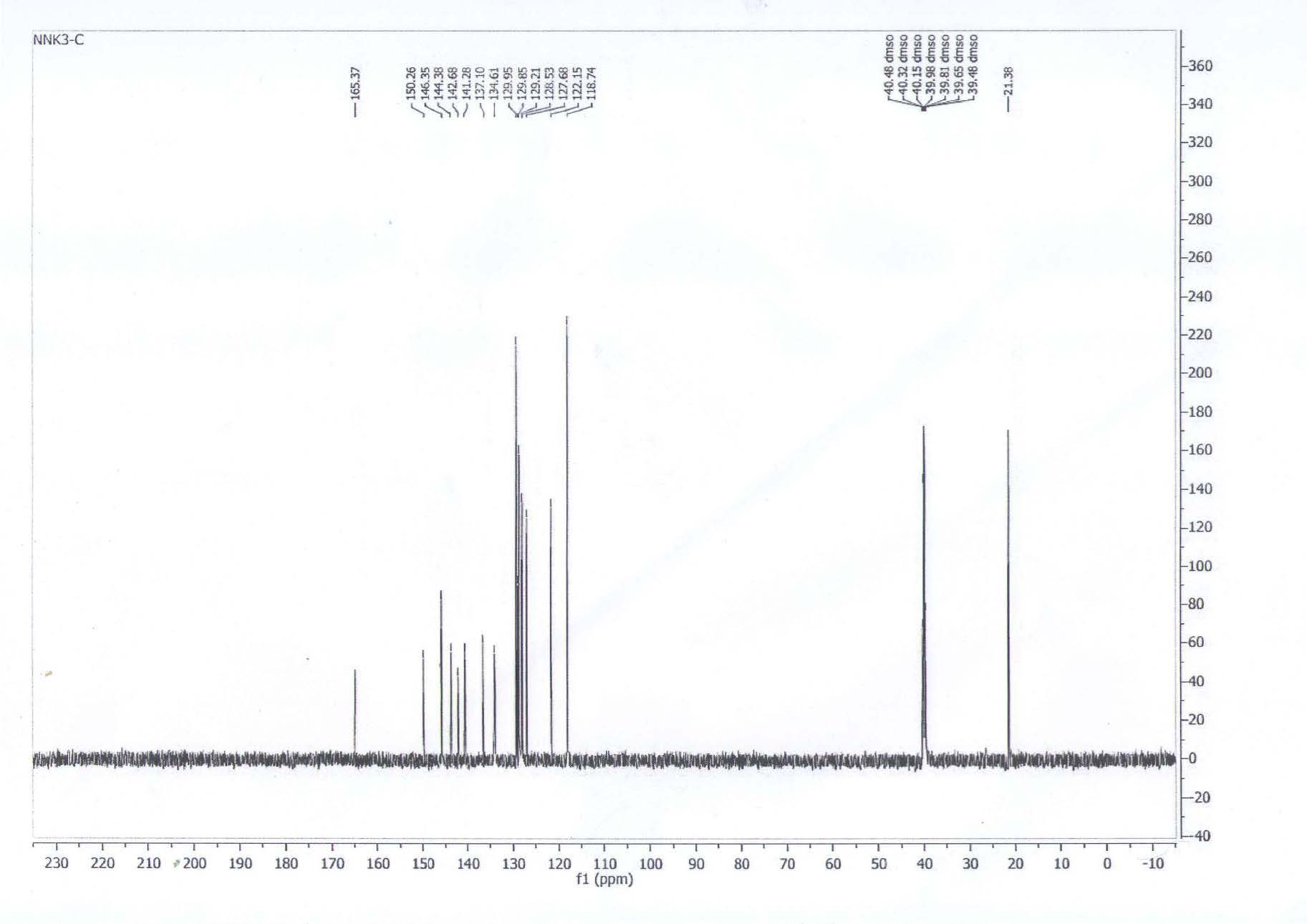


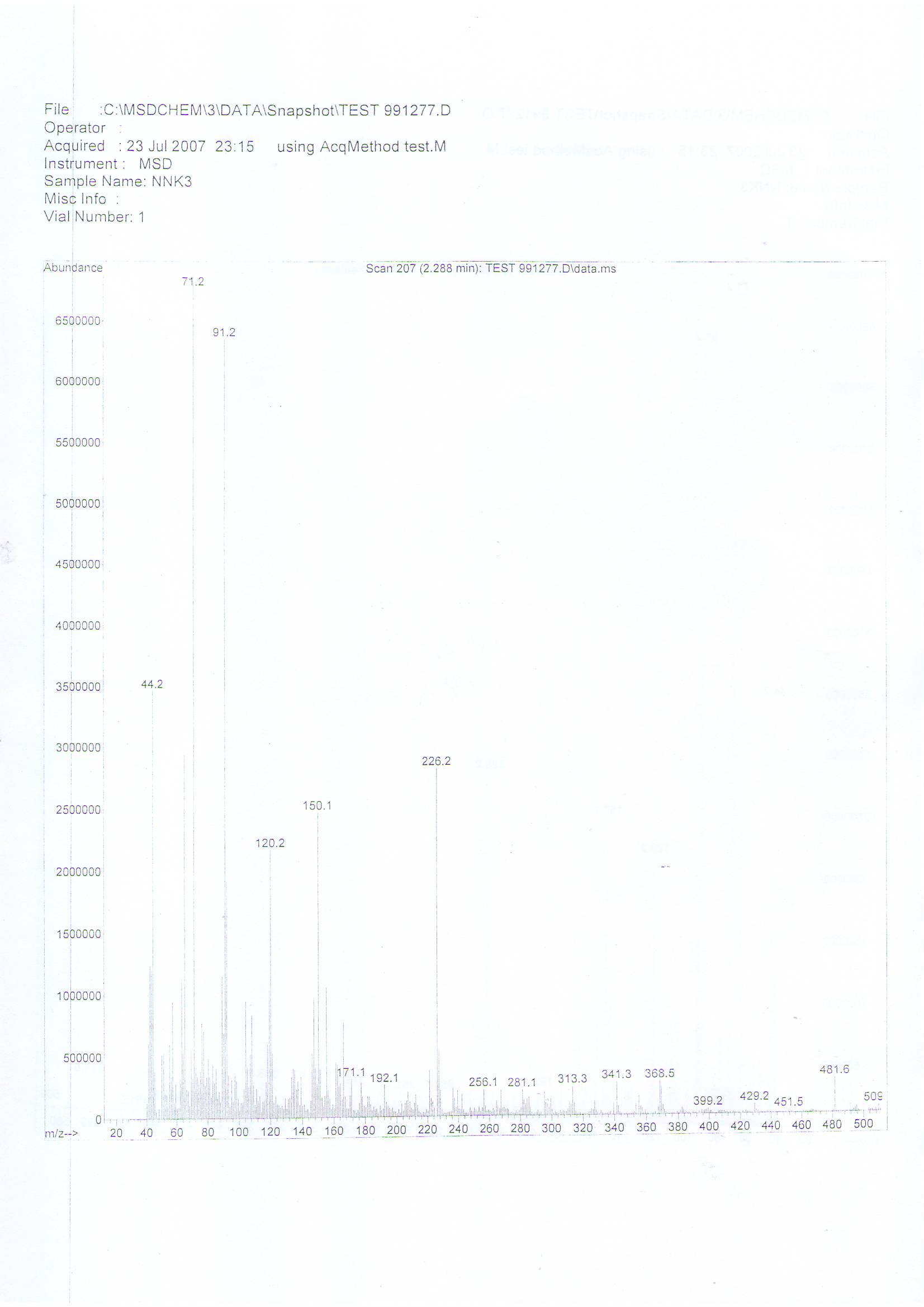


**(4h)**

1-(4-(2-(4-chlorobenzylidene)hydrazine-1-carbonyl)phenyl)-3-(4-chlorophenyl)urea


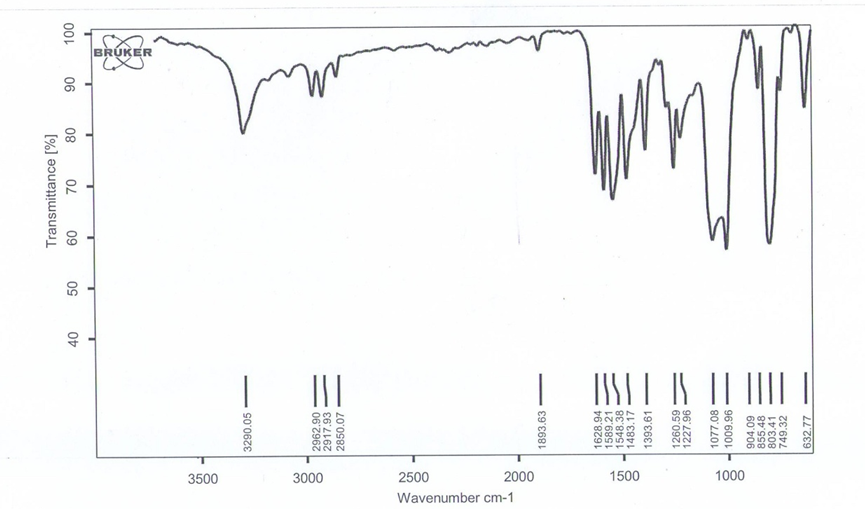


7.8886-7.8715


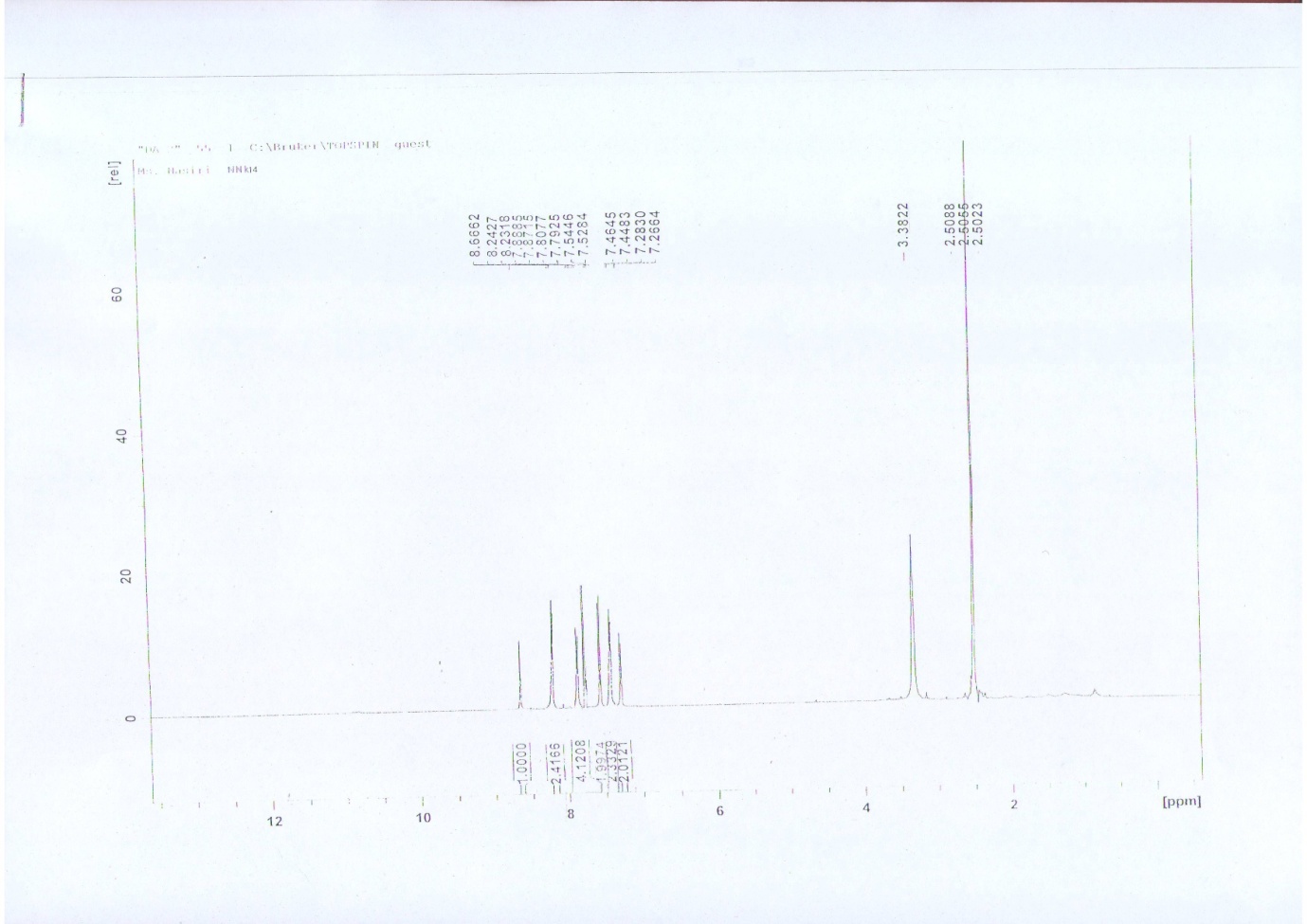


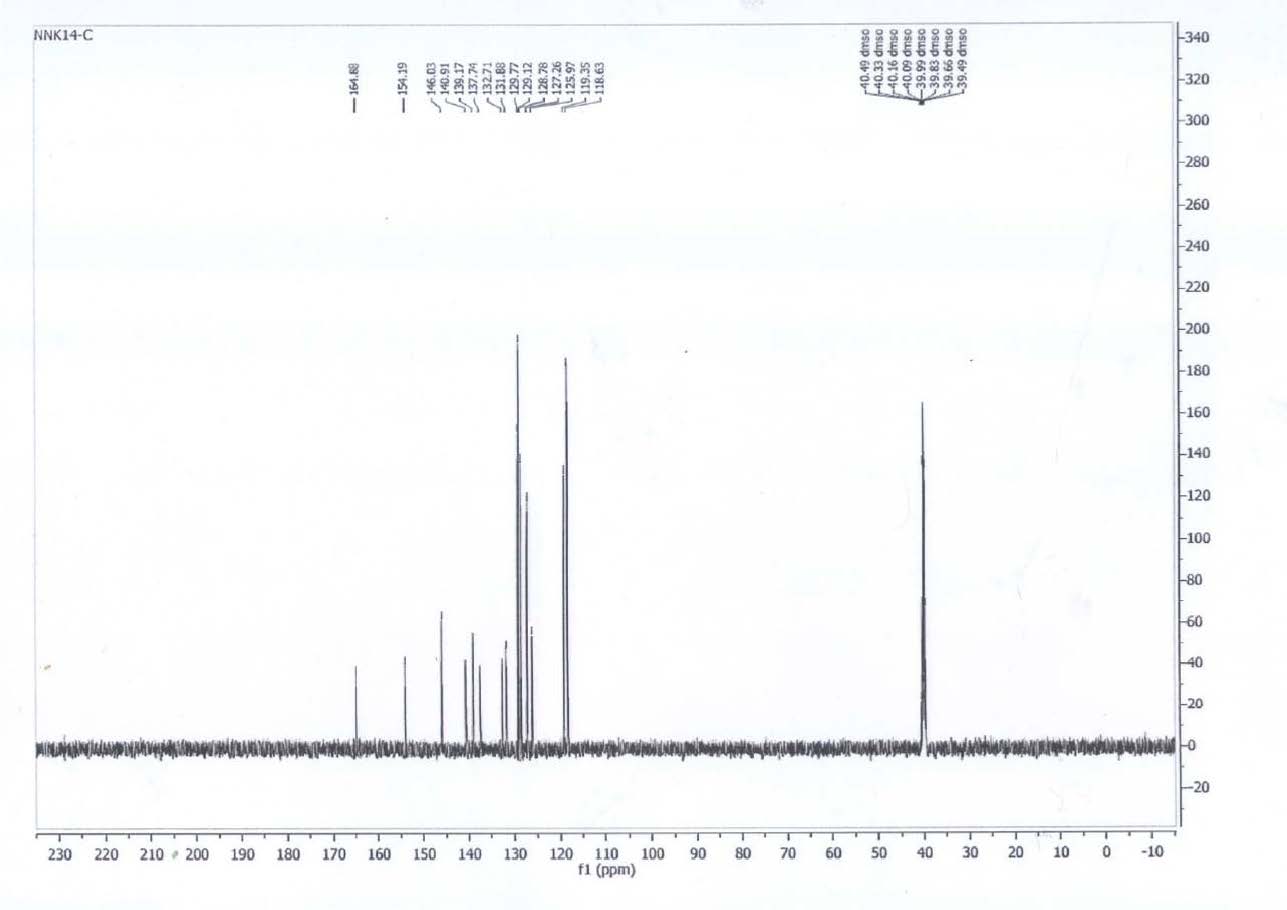


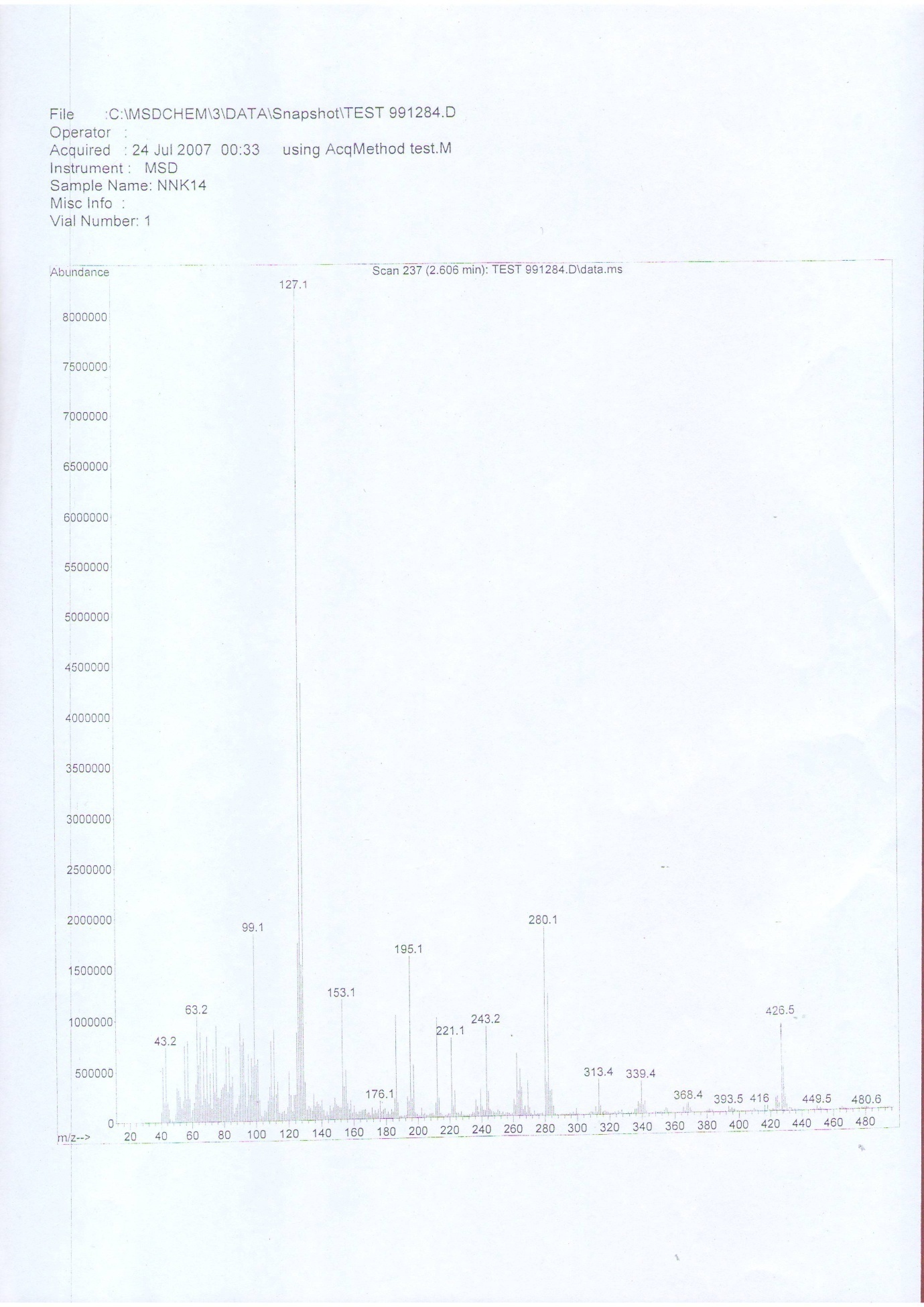


**(4i)**

4-methyl-N-((4-(2-(pyridin-4-ylmethylene)hydrazine-1-carbonyl)phenyl)carbamoyl) benzenesulfonamide

*
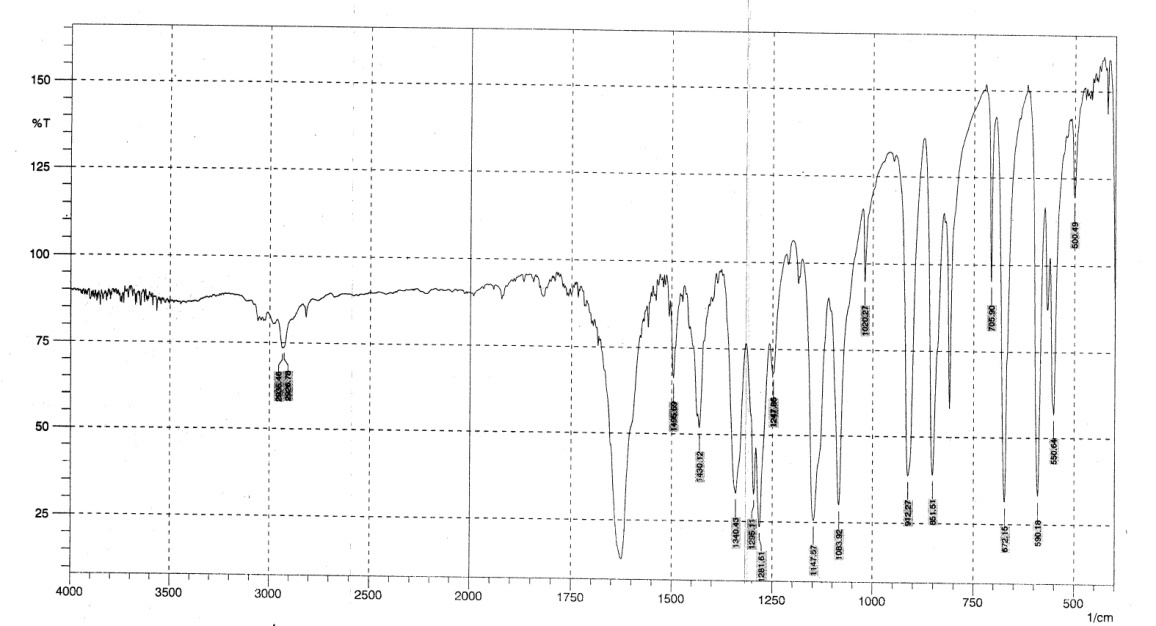
*


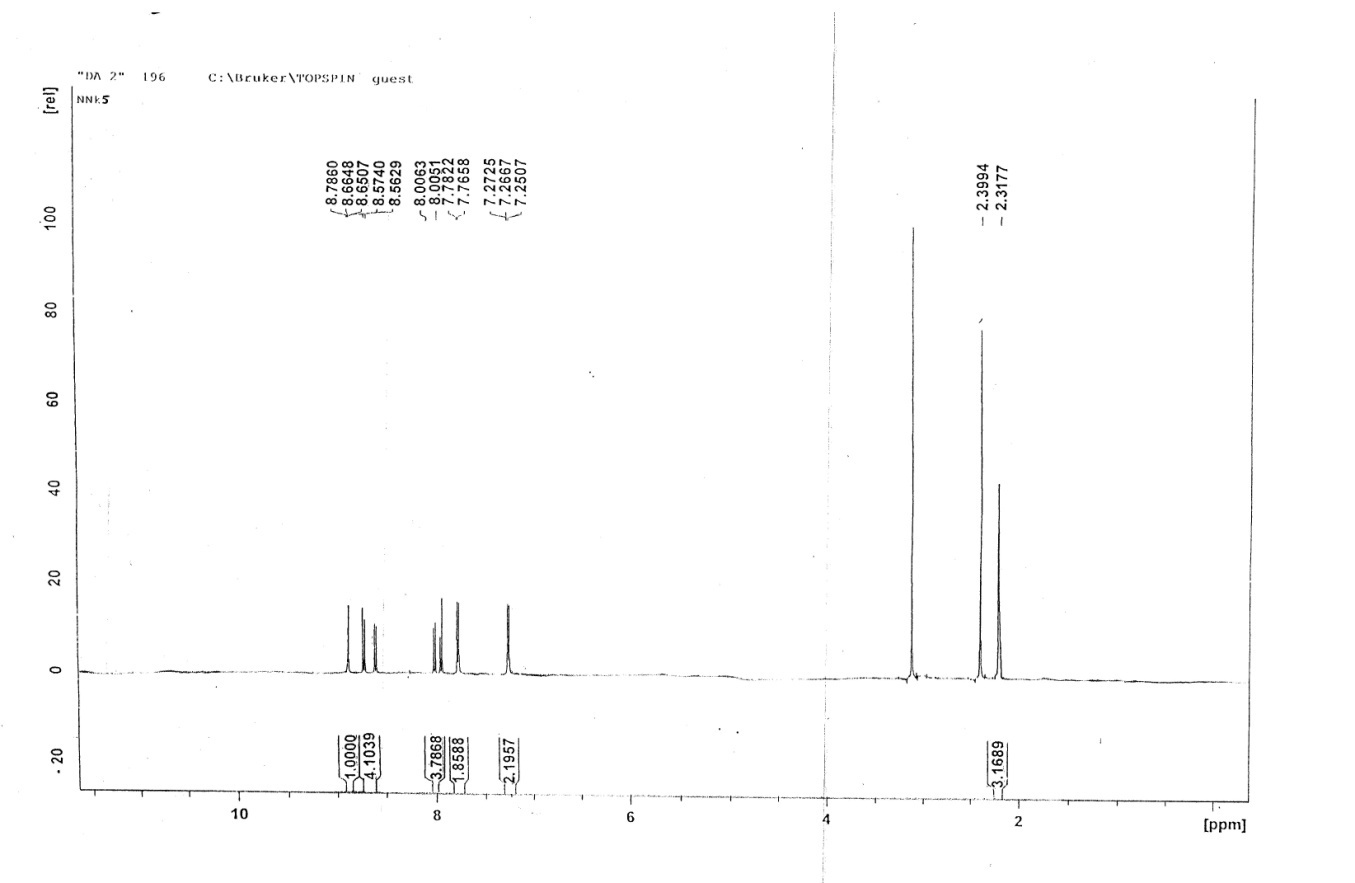


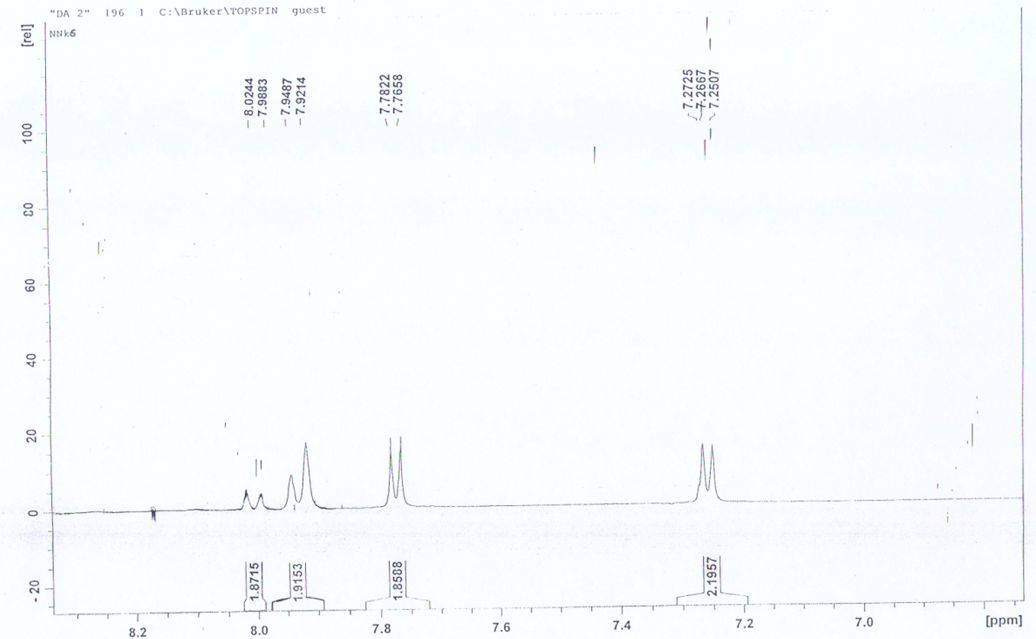


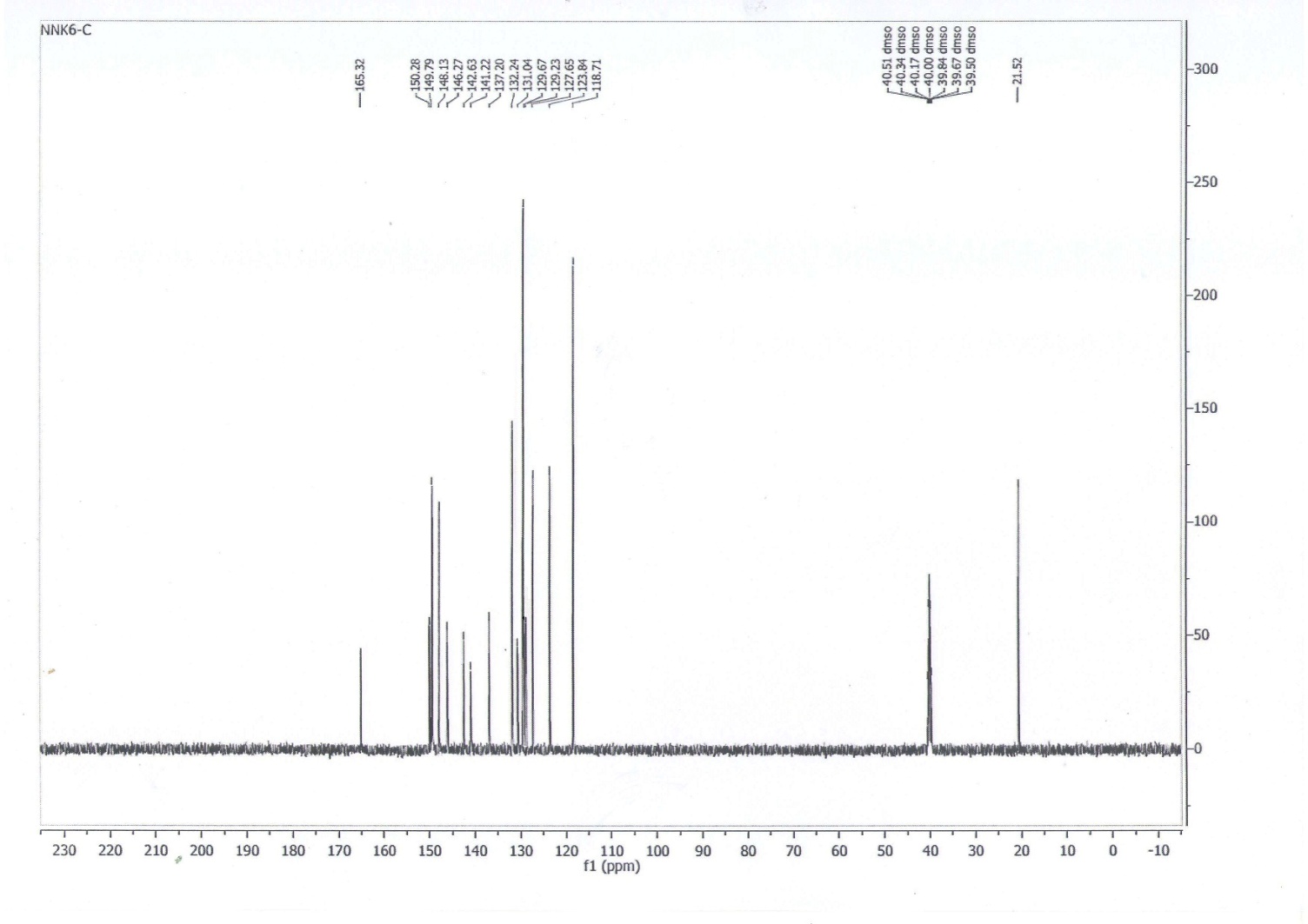


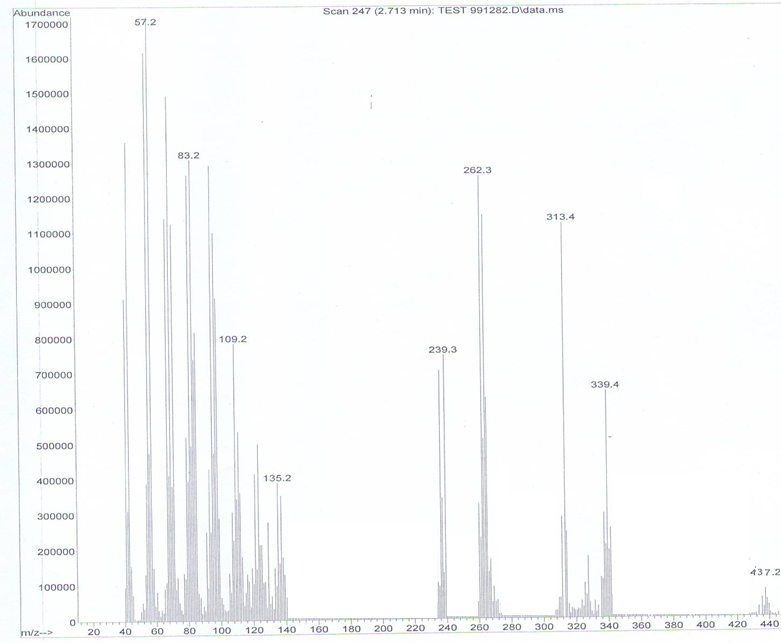


***Correspondence to:**

Dr Ali Almasirad, Department of Medicinal Chemistry, Faculty of Pharmacy,Tehran Medical Sciences, Islamic Azad University,P.O. Box1941933111, Tehran, Iran.

E-mail: almasirad.a@iaups.ac.ir.

Dr. Mona Salimi, Department of Physiology and Pharmacology, Pasteur Institute of Iran, P.O. Box 1316943551, Tehran, Iran.

E-mail: salimimona@pasteur.ac.ir
