# Supplementary material for: Design and synthesis of novel ureido and thioureido conjugated hydrazone derivatives with potent anticancer activity
Source: BMC Chem. 2022 Nov 1;16(1):81. doi: 10.1186/s13065-022-00873-3 (PMC9624014; doi:10.1186/s13065-022-00873-3)
Supplement: Supplementary file 2 — Additional file 2: Table S1. Cytotoxicity screening (%) for compounds 4a–4i following treatment at 10 μM for 72 h, towards human cancer cell lines. Table S2. IC50 values for cytotoxic activity of doxorubicin towards cancer cells at 72 h. [file 13065_2022_873_MOESM2_ESM.doc]

# Table S1. Cytotoxicity screening (%) for compounds 4a-4i following treatment at 10 μM for 72 h, towards human cancer cell lines.^a^

|  |  | **Compound**      **Cell Line** |
| --- | --- | --- |
| **HT-29** | **HepG2** |
| 45.86±13.41 | 37.37±3.46 | **4a** |
| 28.75±3.65 | 28.32±3.78 | **4b** |
| 64.37±15.08 | 67.06±2.53 | **4c** |
| 26.05±3.39 | 38.60±1.11 | **4d** |
| 62.02±2.38 | 60.69±0,87 | **4e** |
| 33.57±5.57 | 49.28±3.95 | **4f** |
| 42.69±0.68 | 29.73±1.99 | **4g** |
| 36.08±4.62 | 33.22±2.53 | **4h** |
| 13.68±5.74 | 26.86±3.11 | **4i** |

^a^ Values were determined at least three independent experiments each performed in triplicate and expressed as mean ± SEM.

**Table S2** IC_50_ values for cytotoxic activity of doxorubicin

towards cancer cells at 72 h^a^

| **Compound IC_50_ (µM)** | | |
| --- | --- | --- |
| HT-29 | HepG2 |  |
| 2.5±0.2 | 1.8±0.4 | **Doxorubicin** |

***Correspondence to:**

Dr Ali Almasirad, Department of Medicinal Chemistry, Faculty of Pharmacy,Tehran Medical Sciences, Islamic Azad University,P.O. Box1941933111, Tehran, Iran.

E-mail: almasirad.a@iaups.ac.ir.

Dr. Mona Salimi, Department of Physiology and Pharmacology, Pasteur Institute of Iran, P.O. Box 1316943551, Tehran, Iran.

E-mail: salimimona@pasteur.ac.ir
